# Metabolomic Insights into Smoking-Induced Metabolic Dysfunctions: A Comprehensive Analysis of Lipid and Amino Acid Metabolomes

**DOI:** 10.3390/metabo15020096

**Published:** 2025-02-04

**Authors:** Muhammad Amtiaz Aslam, Hajra Iqbal, Kainat Ilyas, Kanwal Rehman, Amjad Hussain, Muhammad Sajid Hamid Akash, Mudassar Shahid, Shuqing Chen

**Affiliations:** 1 Department of Pharmaceutical Chemistry, Government College University, Faisalabad 38000, Pakistan; 2 Department of Pharmacy, The Women University, Multan 60000, Pakistan; 3 Department of Chemistry, University of Okara, Okara 56300, Pakistan; 4 Department of Pharmaceutics, College of Pharmacy, King Saud University, Riyadh 11451, Saudi Arabia; 5 Institute of Drug Metabolism and Drug Analysis, College of Pharmaceutical Sciences, Zhejiang University, Hangzhou 310058, China

**Keywords:** nicotine exposure, lipid metabolism, amino acid profiling, oxidative stress, metabolomic analysis

## Abstract

Background: Cigarette smoking is a leading cause of preventable mortality, largely due to the absence of effective, non-invasive biomarkers for early disease detection. Profiling serum metabolomics to identify metabolic changes holds the potential to accelerate the detection process and identify individuals at risk of developing smoking-related diseases. Objectives: This study investigated the biochemical and metabolomic changes induced by nicotine exposure, with a focus on disruptions in amino acid, lipid, and carbohydrate metabolism. Methods: Liquid chromatography–tandem mass spectrometry (LC-MS/MS) was employed to observe significant disruptions in lipid and amino acid metabolism, along with alterations in key metabolic pathways. A total of 400 smokers and 100 non-smokers were included to evaluate the biomarkers related to insulin resistance, blood lipid profile, inflammation, and kidney and liver function. Results: The results demonstrated significantly elevated (*p* < 0.05) levels of glycemic markers in smokers, including fasting blood glucose; glycated hemoglobin (HbA1c); and inflammatory markers such as interleukin-6 (IL-6) and C-reactive protein (CRP). Smokers also exhibited dyslipidemia, with increased total cholesterol (154.888 ± 35.565) and LDL levels (117.545 ± 24.138). Impaired liver and kidney function was evident, with significantly higher levels (*p* < 0.05) of AST, ALP, ALT, blood urea nitrogen, and creatinine in smokers. A total of 930 metabolites were identified, of which 343 exhibited significant alterations (*p* < 0.05) in smokers compared to non-smokers. Among these, 116 metabolites were upregulated, and 127 were downregulated. Metabolomic pathway analysis revealed eight significant pathways. The study also identified three lipid metabolites specific to smokers and seven unique to non-smokers. Through LC-MS/MS, fragments of phenylalanine, tryptophan, valine, histidine, carnitine, and sphinganine were detected. Several lipidomic changes associated with insulin resistance and cardiovascular complications were observed. Cadmium (Cd) levels were higher in smokers than non-smokers (1.264 ppb vs. 0.624 ppb) and showed a strong negative correlation (R^2^ = 0.8061, *p*-value = 0.015) with serum zinc (Zn), likely due to Cd displacing Zn in proteins and causing nephrotoxicity through accumulation. Conclusions: This study highlights the distinct metabolic disruptions caused by smoking that could serve as potential biomarkers for the early detection of metabolic diseases. It emphasizes the importance of metabolomics in identifying systemic indicators of smoking-related health issues, providing new opportunities for preventive and therapeutic interventions.

## 1. Introduction

Nicotine, the addictive component of tobacco, plays a crucial role in sustaining smoking behavior. It stimulates the release of neurotransmitters such as dopamine, inducing pleasurable sensations and reinforcing the habit [1]. Quitting smoking is notoriously challenging due to nicotine addiction, often requiring a combination of behavioral interventions and pharmacotherapy to improve the chances of success. Public health efforts to reduce smoking rates have included awareness campaigns; smoking cessation programs; and legislative measures, such as smoking bans and increased taxation [2]. Although these initiatives have led to a reduction in smoking rates in many countries, smoking remains a significant public health challenge. The 3-hydroxycotinine/nicotine ratio has been investigated as a potential predictor of pharmacotherapy success [3]. Despite extensive research into nicotine and its toxic metabolites, the precise mechanisms underlying its harmful effects remain poorly understood [4,5].

Research indicates that smoking induces significant changes in lipid metabolism, particularly affecting phospholipids and branched-chain amino acids (BCAAs). These alterations activate inflammatory pathways and oxidative stress, contributing to various health complications, especially in patients with chronic obstructive pulmonary disease. Smoking has also been shown to alter levels of ceramide and sphingosine-1-phosphate [6]. Additionally, smoking is associated with changes in cholesterol and triglyceride levels, increasing the risk of cardiovascular diseases. Cigarette smoking rapidly elevates the flux of free fatty acids (FFAs) and glycerol, leading to elevated circulating FFA levels, primarily driven by nicotine-induced lipolysis [7].

Nicotine, the primary active component in cigarettes, disrupts insulin signaling, particularly in skeletal muscle by phosphorylating serine residues on insulin receptor substrate-1 (IRS1). This interferes with IRS1’s role in the insulin signaling pathway. Smoking also affects the metabolism of several amino acids, including glycine, threonine, cysteine, and homocysteine, leading to amino acid imbalances that impair respiratory system function in smokers [8]. These metabolic disruptions significantly contribute to the development and progression of smoking-related illnesses, such as cancer and cardiovascular disorders [9]. Furthermore, smokers with hypertension exhibit reduced levels of metabolites such as tetradecanedioic acid, hippuric acid, glyceric acid, 20-hydroxyeicosatetraenoic acid, phenylpyruvic acid, and capric acid, along with increased levels of 7-ketodeoxycholic acid, serotonin, N-stearoyl tyrosine, and deoxycholic acid glycine conjugate [10]. Biomarkers of oxidative damage, such as 8-hydroxydeoxyguanosine (8-OHdG) and malondialdehyde (MDA), are significantly elevated in smokers and users of heated tobacco products, reflecting heightened oxidative stress [11].

Moreover, tobacco leaves contain cadmium at concentrations of 1–2 μg/gram, exposing smokers to cadmium oxide. This compound binds to thiol groups in cysteine and glutathione, inducing toxicity in the liver and kidneys. Studies have shown that smokers have blood cadmium levels 4–5 times higher than non-smokers, with cadmium toxicity mechanisms primarily involving oxidative stress and tissue damage [12].

Metabolomics is a cutting-edge approach that provides a comprehensive analysis of metabolites within a biological system, offering valuable insights into the biochemical effects of various substances, including nicotine, on human health [13]. This advanced technique is particularly effective in studying metabolic alterations caused by both external and internal stimuli. Metabolomics involves identifying and quantifying metabolites in biological samples such as tissues, blood, and urine, providing an instant snapshot of an organism’s physiological state [14]. Both targeted and non-targeted metabolomics approaches allow researchers to discover new metabolites and pathways associated with specific biological conditions or treatments. Nicotine, a major component of cigarette smoke, has profound effects on the body’s metabolic pathways. Inhalation of nicotine induces immediate and long-term changes in the metabolome—the complete set of metabolites in an organism. Studies utilizing metabolomics have revealed distinct metabolic profiles in smokers compared to non-smokers.

This study provides a comprehensive investigation of the biochemical and metabolomic alterations induced by nicotine exposure, focusing on its impact on lipid and amino acid metabolism. Using a human model, the research aims to elucidate the mechanisms by which nicotine disrupts critical metabolic processes, contributing to the development of metabolic disorders such as diabetes mellitus. The study examines how smoking affects lipid profiles, amino acid levels, inflammatory markers, and essential metal concentrations, while also exploring its role in oxidative stress and insulin resistance. Through comprehensive metabolomic analysis, this research elucidates specific biomarkers and molecular pathways implicated in smoking-induced metabolic dysfunctions. These findings provide valuable insights into early diagnostic approaches and the development of targeted therapeutic interventions to mitigate these metabolic disorders.

## 2. Materials and Methods

### 2.1. Study Design

The study included two groups of participants from the same ethnic background: one group of healthy non-smokers and another comprising healthy cigarette smokers. Blood samples were collected following established protocols, and biochemical and analytical tests were conducted at the Pharmaceutical Chemistry Laboratory of Government College University Faisalabad, Pakistan. After obtaining informed consent, 500 volunteers (100 controls and 400 smokers) were recruited. Demographic information and medical history were recorded using a structured questionnaire completed by each participant. Control subjects were selected from the Gujranwala and Lahore districts, with strict exclusion criteria applied to ensure the reliability of the results. Individuals were excluded if they had ongoing or previous cancer, respiratory or oral cavity diseases, recent exposure to general anesthesia, use of antidepressants or smoking cessation medications, psychological disorders, or any conditions that might impair their ability to understand the consent process.

### 2.2. Biochemical Analysis

Blood samples collected in gel vacutainers were allowed to sit at room temperature for 30 min to facilitate clot formation. The clotted blood was then centrifuged, and the serum was extracted and stored at −20 °C for subsequent analysis. Additionally, a portion of the blood was collected in EDTA tubes for tests requiring plasma or whole blood. Biochemical parameters were measured using commercially available kits on a Selectra ProM clinical chemistry analyzer.

#### 2.2.1. Determination of Serum Glucose

Serum glucose levels serve as a vital indicator of diabetes mellitus and play a critical role in detecting metabolic disturbances. This test measures glucose levels in the liquid component of blood. In this study, serum glucose levels were quantified using a diagnostic reagent from AMS S.p.A, Florence, Italy, and analyzed with a photometric system (Selectra ProM).

#### 2.2.2. Determination of Serum Insulin

A serum insulin test, also known as a blood insulin test, measures the amount of insulin in the bloodstream. Insulin, a hormone produced by the pancreas, is essential for regulating blood glucose levels. In this study, serum insulin levels were determined using the sandwich electrochemiluminescence immunoassay (ECLIA) method. The Roche Elecsys Insulin assay kit (Reference No. 12017547122) and the cobas e601 analyzer by Roche Diagnostics GmbH (Vienna, Austria) were used for this analysis.

#### 2.2.3. Determination of Insulin Resistance

Fasting serum insulin and glucose levels were measured to assess insulin resistance using the Homeostatic Model Assessment of Insulin Resistance (HOMA-IR). The calculation was performed using the following equation:

HOMA-IR = Fasting insulin (µU) × serum glucose level (mg/dL)/450(1)

#### 2.2.4. Determination of HbA1c Levels

HbA1c levels were measured using the HbA1c assay kit (Catalog No: HOEM0904, Hzymes Biotech, Wuhan, China) in accordance with the manufacturer’s instructions.

#### 2.2.5. Lipid Biochemical Parameters

The lipid profile was analyzed using the Selectra ProM analyzer and the kit method. The biochemical parameters assessed included low-density lipoprotein (LDL), high-density lipoprotein (HDL), triglycerides (TGs), and total cholesterol. All chemical kits were provided by Wiener Lab., Rosario, Argentina, and the tests were conducted in accordance with the manufacturer’s protocols.

#### 2.2.6. Estimation of Kidney Function Biomarkers

Serum urea and creatinine levels were measured using assay kits from Wiener Lab., Argentina, in accordance with the manufacturer’s instructions. Quantification was conducted in a controlled laboratory setting through an in vitro process, utilizing the advanced photometric system of the Selectra ProM Chemistry Analyzer along with the diagnostic reagents.

#### 2.2.7. Assessment of Liver Biomarkers

Liver function biomarkers, including aspartate aminotransferase (AST), alkaline phosphatase (ALP), and alanine aminotransferase (ALT), were assessed in serum using an assay kit from Wiener Lab., Argentina. Absorbance measurements were performed using the Selectra ProM Chemistry Analyzer.

#### 2.2.8. Estimation of Inflammatory Biomarkers

Inflammatory mediators, such as C-reactive protein (CRP), were quantified in serum using ELISA kits from Merck, France. Interleukin-6 (IL-6) levels were measured using the ECLIA method, utilizing the Roche Elecsys IL-6 assay kit (Reference No. 07027532190) and the cobas e801 analyzer from Roche Diagnostics GmbH.

### 2.3. Estimation of Cadmium and Zinc in Serum by ICP-OES

#### 2.3.1. Reagents and Standard Solutions

Reagents from Merck (Darmstadt, Germany) were of supra-pure quality, and only highly pure deionized water was used for all experimental procedures. Ultrapure HNO_3_ was sourced from Sigma-Aldrich. Working standards for ICP-OES analysis were prepared from 1000 ppm standard solutions of each tested element, supplied by Perkin Elmer (Shelton, CT, USA). All other reagents were of analytical grade. Solutions were stored in polyethylene tubes, while all glassware, plastic tubes, and autosampler cups were pretreated by soaking in 10% v/v HNO_3_ for 24 h, followed by rinsing with distilled water. To minimize contamination risks from ambient air and dust, all procedures were performed within a clean bench environment.

#### 2.3.2. Conventional Digestion of Samples for ICP-OES Analysis

The concentrations of Cd and essential metals were measured using ICP-OES. Serum samples were collected in metal-free polypropylene containers using a clean-catch method and stored until analysis. Prior to analysis, serum samples were thawed at room temperature. In total, 1 mL of blood was treated with 3 mL of HNO_3_ and 1 mL of HClO^4^ in cleaned porcelain beakers and left overnight. The sample preparation followed a previously established method with slight modifications [15,16]. The acid-treated samples were then heated on an electric hot plate at 80 °C for 2 to 3 h until they became clear and transparent. The resulting solution was diluted to 25 mL with deionized water, yielding a clear, colorless liquid. This was stored in polyethylene bottles at 4 °C until ICP-OES analysis (Teledyne Leeman Labs Prodigy 7). Blank preparations were also conducted, and normal control samples were treated in the same manner. For ICP-OES analysis, samples, including calibration blanks, standards, reagent blanks, and control samples, along with matrix modifiers, were introduced. Calibration was regularly checked by analyzing standards every 10 readings. All procedures were carried out at room temperature (25 °C) following established laboratory protocols.

### 2.4. Qualitative Analysis of Metabolomes with LC-MS/MS

Blood samples from both groups were analyzed using LC-MS/MS-based metabolomics.

#### 2.4.1. Sample Preparation for LC–MS/MS Analysis

Serum was collected by centrifuging blood at 3500× *g* for 10 min at 4 °C and then stored at −80 °C in preparation for both qualitative and quantitative metabolomics analysis. For protein precipitation, 10 μL of serum was mixed with methanol and incubated for 10 min, followed by centrifugation at 16,000× *g* for 15 min to separate the supernatant. The residue was dried under nitrogen gas and reconstituted in 20 μL of pure methanol [17]. For metabolomic analysis, 10 μL of the supernatant was injected into the LC-MS/MS system at the Pakistan Council of Scientific & Industrial Research (PCSIR) in Lahore, Pakistan.

#### 2.4.2. Conditions of the Instrument

The instrument was operated according to the specified instrumental and operational parameters, as detailed in Table 1. The system used in this study was an Agilent 6495C triple quadrupole LC-MS/MS, coupled with an electrospray ionization source (ESI). A 2 µL sample was injected into an Agilent Zorbax Extend-C18 Rapid Resolution HT column (2.10 × 100 mm; 1.80 µm). An Agilent Technologies low-dispersion in-line filter with 2 µm frits was placed just before the analytical column. For chromatographic separation, a gradient of 0.1% formic acid in acetonitrile for mobile phase B and 0.1% formic acid in water for mobile phase A was used. The gradient increased from 2% to 20% B over 0 to 6 min; from 6 to 9 min, it rose to 45% B; and from 9 to 14 min, it reached 98% B. The column temperature was maintained at 35 °C, with a mobile phase flow rate of 0.35 mL/min [18]. Data were collected in positive ESI (ESI^+^), with the capillary voltage set at 3 kV. The *m*/*z* range was set from 50 to 1000 to acquire the required data. The wide *m*/*z* range spectrum was divided into smaller segments, with each segment scanned individually to generate narrower-range spectra during LC-MS/MS-based data collection. The table provides additional details on the operational and instrumental parameters.

#### 2.4.3. Annotation and Identification

Putative annotations were generated using online spectrum libraries and in silico technology. By comparing the measured mass spectra with online spectra for mass accuracy, retention times, and isotope patterns, annotations were automatically produced. For comprehensive metabolite and lipidomic profiling, MzMine 4.0.8 and MS-DIAL 4.2 were used in tandem to annotate and identify LC-MS/MS data. The raw data were first processed using MzMine 4.0.8, which performed detection, alignment, and peak deconvolution to generate a peak list. MS-DIAL 4.2 was then employed to annotate lipids by matching experimental spectra with the built-in spectra library. To minimize false positives and ensure high accuracy, parameters such as retention time tolerance and mass accuracy criteria (typically < 10 ppm) were applied. For metabolite identification, a spectrum similarity cut-off of 85% was used. The NIST spectral library was utilized to annotate non-lipid metabolites. Metabolites were identified by comparing experimental MS/MS spectra with reference spectra from the NIST library, which contains a comprehensive collection of standard mass spectra for small compounds.

### 2.5. Statistical Analysis

Statistical analysis was performed using GraphPad Prism 5 (GraphPad Software Inc., La Jolla, CA, USA), which included Student’s *t*-test and descriptive analysis. Pearson correlation was employed to identify significant differences across groups, with the probability threshold set at *p* < 0.05. Mean values and standard deviations (SDs) were graphically represented in the data presentation. For metabolomic quantification, fold-change calculations and paired *t*-tests were performed using Microsoft Excel 365. Correlations between Cd and zinc were assessed using Pearson correlation in GraphPad Prism 9.5.1, with *p*-values and r-values calculated. MetaboAnalyst 6.0, an open-access online platform, was used for univariate statistical analysis of metabolomic data. To ensure comparability among metabolites, the data were normalized using auto-scaling in MetaboAnalyst 6.0. A Student’s *t*-test was employed to identify metabolites with statistically significant differences in mean intensities between two groups. Biological significance was determined based on a fold change (FC) threshold of ±1.5, and a *p*-value < 0.05 was considered significant. LipidSig 2.0, another online platform, was utilized to extract lipidomic-specific insights from the metabolomic dataset.

## 3. Results

### 3.1. Biochemical Analysis

The biomarker analysis for both groups was conducted using serum samples from participants. The mean values and SDs of various biomarkers are presented in Table 2. Biochemical analytical tests provide critical insights into the chemical composition and functioning of compounds within the human body or a sample. The results of these tests can vary significantly depending on the specific test performed and the baseline ranges used by the laboratory. The analysis revealed significant differences in the levels of these biomarkers between smokers and non-smokers, with smokers exhibiting notable alterations in their blood profiles.

#### 3.1.1. Effect on Glycemic Index Parameters

**Figure 1:**
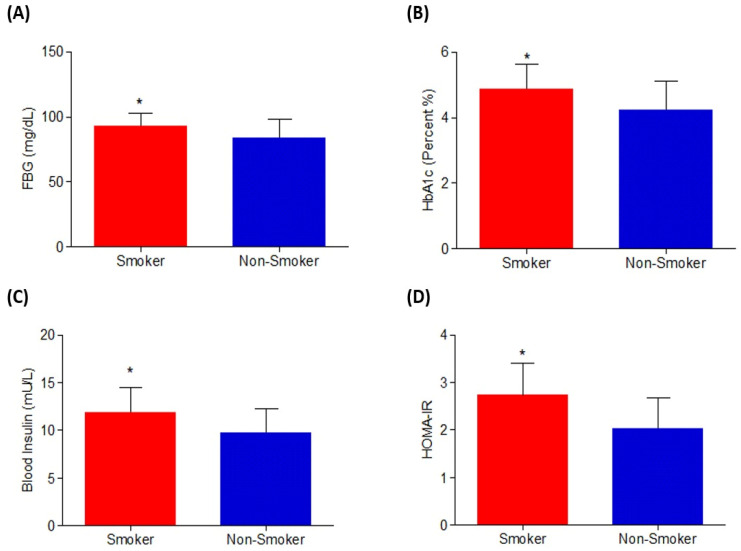
Participants’ (**A**) fasting blood glucose, (**B**) serum HbA1c, (**C**) blood insulin, and (**D**) HOMA-IR are displayed. The significance level was assessed using Student’s *t*-test, which revealed a significant difference (*p* < 0.05) between the two groups, the smokers and the non-smokers. The findings are given as mean ± SD. * represent the level of significant difference when compared to the smokers with non-smokers.

The glycemic index biomarkers for smokers and non-smokers were assessed, with fasting blood glucose (FBG) and HbA1c levels presented in Table 2. Smokers exhibited significantly higher FBG values (*p* = 0.0036) compared to non-smokers (Figure 1A), indicating a potential link between smoking and poor glycemic control. Serum insulin levels were also elevated in smokers, resulting in significantly higher HOMA-IR values in the smoker group compared to the non-smoker group (*p* = 0.041).

#### 3.1.2. Effects on Liver, Inflammatory, Kidney Biomarkers, and Lipid Profile

**Figure 2:**
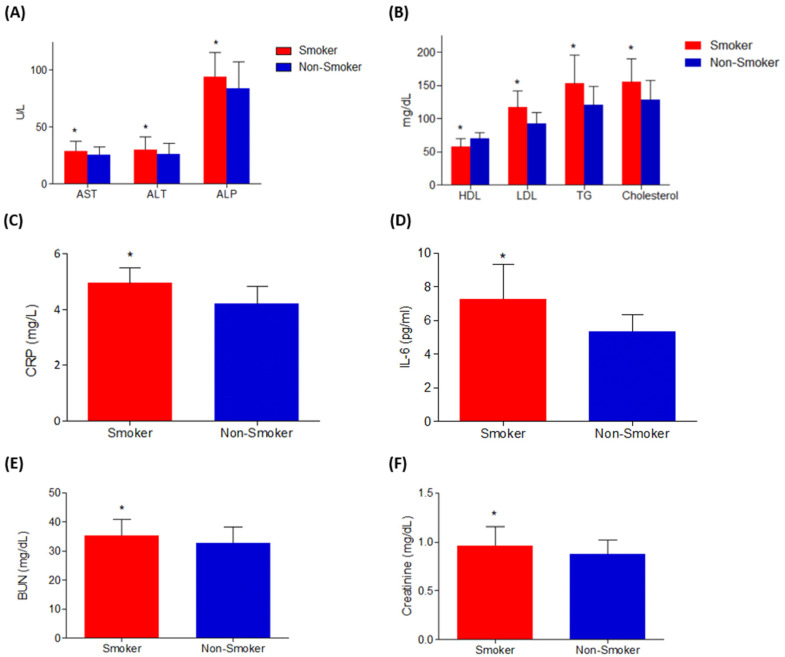
The serum levels of (**A**) AST, ALT, and ALP; (**B**) HDL, LDL, and TG; (**C**) CRP; (**D**) IL-6; (**E**) blood urea; and (**F**) creatinine levels are represented. Student’s *t*-test was used to assess the significance level, showing a notable difference between the two groups. The results showed that there was a significant difference (*p* < 0.05) between the two groups, the smokers and the non-smokers. * represent the level of significant difference when compared to the smokers with non-smokers.

The results of our investigation demonstrated that smokers had significantly higher levels of AST, ALT, and ALP (*p* < 0.05) compared to non-smokers (Figure 2A), suggesting a potential association between smoking and hepatocellular damage and liver dysfunction. Inflammatory markers, including CRP and IL-6, were also notably higher (*p* = 0.0062 and 0.0049) in smokers compared to non-smokers (Figure 2C). Regarding renal function, serum creatinine and blood urea levels were significantly elevated (*p* = 0.04 and 0.0008) in the smoker group relative to the non-smoker group (Figure 2D). Furthermore, smokers exhibited significantly higher blood levels of triglycerides, cholesterol, and LDL (*p* = 0.002, 0.00062 and 0.0013), while their HDL levels were significantly lower (*p* = 0.03) compared to non-smokers (Figure 2B).

### 3.2. Analysis of Metabolomes by LC-MS/MS

We collected data using ESI^+^ mode, maintaining a capillary voltage of 3 kV, with an *m*/*z* range set between 50 and 1000 for data acquisition. Peak selection was based on the precise molecular mass of the metabolite of interest and the corresponding fragmentation ion peaks. A literature review confirmed the characteristic (precursor) ion peaks and their corresponding fragment ion peaks, which facilitated the identification of various metabolites, including amino acid metabolites and lipid metabolites across different classes. In total, 930 metabolites were identified in positive ion mode in both groups (Supplementary Table S1).

**Figure 3:**
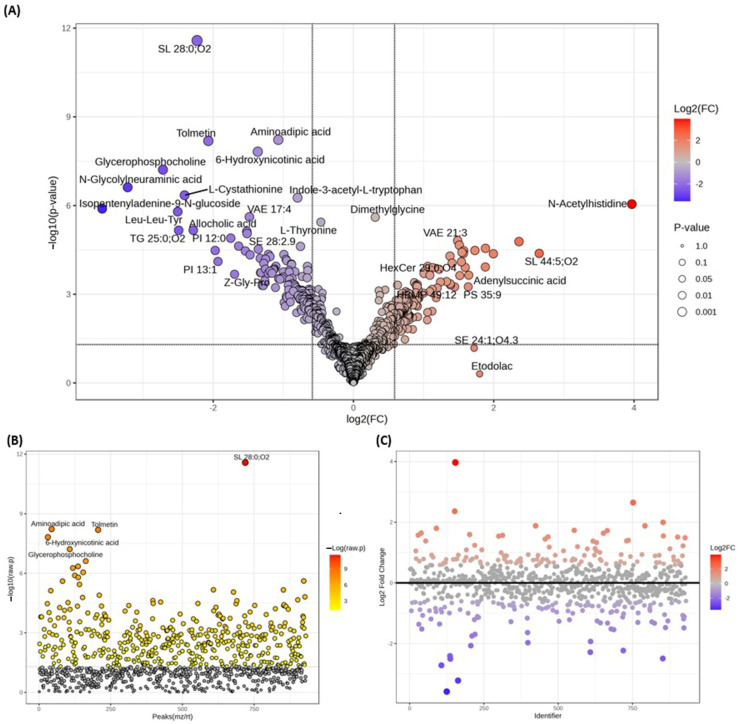
Representation of important features selected by (**A**) volcano plot with fold-change threshold of (x) 1.5 and *t*-test threshold of (y) 0.05; (**B**) *p*-value; and (**C**) fold change. The red circles represent features above the threshold. The most significantly dysregulated metabolites were identified using *t*-tests with a significance threshold of 0.05.

Following preprocessing, a data frame containing retention time, *m*/*z* values, and normalized peak intensity was prepared for further analysis. *T*-tests were conducted between smokers and non-smokers to investigate the impact of smoking on the serum metabolomic profile. A total of 343 metabolites showed significant changes (*p* < 0.05) (Supplementary Table S2). After considering fold change values (−1.5 < FC > 1.5), 245 metabolites were identified as dysregulated (Figure 3 and Supplementary Table S3).

The significance of changes in metabolite intensities was determined using *p*-values and fold change (FC) values. In the volcano plot, the *x*-axis represents the FC of the smoker group relative to the non-smoker group, while the *y*-axis represents the *p*-value from the *t*-test comparing the two groups (Figure 3A). A *p*-value threshold of less than 0.05 and a FC cutoff of 1.5 were used to identify significant metabolites. By comparing smokers to non-smokers, 116 metabolites were upregulated (FC > 1.5), and 127 metabolites were downregulated (FC < −1.5), as shown in Supplementary Table S4.

Several metabolites involved in oxidative stress pathways showed statistically significant difference between the study groups (Table 3). These changes reflect a higher level of oxidative stress in the smoker group compared to non-smokers. Notably, biliverdin, a metabolite associated with the heme degradation pathway, was significantly downregulated by three-fold in the serum of smokers relative to non-smokers. Conversely, levels of beta-tocopherol and oxidized glutathione were significantly elevated in the smoker group by 5.3% and 50.13%, respectively. A significant increase in purine (*p* = 0.0014, FC = +1.65) and its degradation products, such as hypoxanthine (40% increase), was also observed in smokers. Moreover, carnosine levels were identified to be significantly (*p* = 0.0013) lower (FC = −0.3) in smokers compared to non-smokers.

Metabolomic analysis further revealed significant alterations in the serum amino acid profiles of smokers. Several amino acids were upregulated in smokers, including isoleucine, valine, alanine, tryptophan, and glutamate. These amino acids exhibited statistically significant increases in concentration, with isoleucine (*p* < 0.05), valine (*p* < 0.05), alanine (*p* < 0.05), tryptophan (*p* < 0.05), and glutamate (*p* < 0.05) all elevated in smokers relative to non-smokers. Conversely, methionine and histidine were downregulated in smokers. Both amino acids showed significantly lower levels compared to non-smokers, with methionine (*p* < 0.05) and histidine (*p* < 0.05) showing substantial reductions. These changes indicate that smoking significantly impacts amino acid metabolism, reflected by the upregulation of certain amino acids and the downregulation of others. Additionally, serum levels of kynurenine and its metabolite, 3-hydroxy kynurenine, were elevated by 32% and 16% in smokers, suggesting a decline in kidney function compared to non-smokers.

Both smoker and non-smoker groups exhibited a variety of lipid molecules, including sterol lipids, glycerophospholipids, glycerolipids, and sphingolipids. The identified lipid classes included diacylglycerols, triglycerides, fatty amides, fatty esters, N-acyl glycine, N-acyl glycyl serine, N-acyl ornithine, phosphatidylethanolamine, phosphatidylserine, glycerophosphoglycerols, ether-linked phosphatidylcholine, ether-linked phosphatidylethanolamine, sphingoid bases, ceramides, hexosyl ceramides, phosphosphingolipids, glycosphingolipids, and bile acid derivatives. The lipid metabolomes were quantified using an Excel sheet and the fold-change technique.

#### 3.2.1. Metabolomic Enrichment Analysis

**Figure 4:**
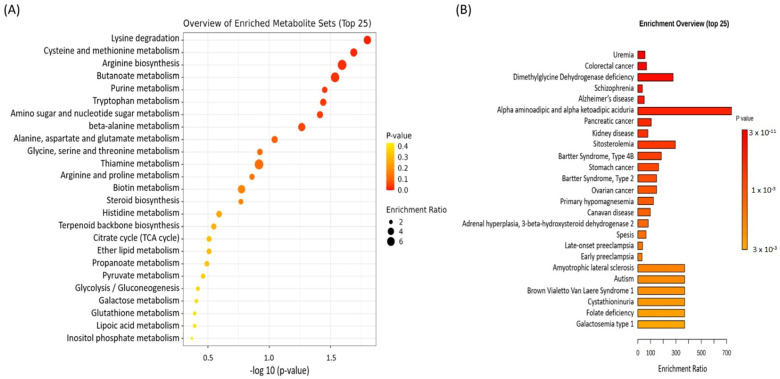
(**A**) Twenty-five significantly enriched metabolic pathways among smokers compared with non-smokers. (**B**) Disease enrichment analysis showing top 25 enriched diseases in smokers. The most significantly dysregulated metabolic pathways were identified using *t*-tests with a significance threshold of 0.05.

When the significant metabolomics data of smokers (excluding lipidomics) were compared with those of non-smokers, 35 metabolic pathways were identified, with 8 pathways showing a *p*-value of less than 0.05. The top 25 pathways, including amino acid, carbohydrate, lipid, and nucleic acid pathways, are illustrated, along with their corresponding *p*-values and enrichment ratios (Table 3 and Figure 4A). These metabolic alterations associated with smoking suggest systemic changes in key biological processes, as selected from the KEGG database. Notably, pathways such as oxidative stress pathways (methionine and cysteine metabolism, glutathione metabolism, and glutamate metabolism) and nucleotide metabolism (purine metabolism) were found to be significantly (*p* < 0.05) impacted.

The disease enrichment analysis identified 83 significant associations between altered metabolites and various disease categories (Supplementary Table S5), with the top 25 illustrated in Figure 4B. Key findings included a strong enrichment of metabolites linked to colorectal cancer, pancreatic cancer, stomach cancer, leukemia, kidney cancer, hepatocellular carcinoma, schizophrenia, Alzheimer’s disease, neurodegenerative diseases, and type 2 diabetes mellitus, all with *p*-values less than 0.05. These associations reflect smoking-related oxidative damage, inflammation, and enhanced DNA damage and repair activities. These findings underscore the systemic effects of smoking on metabolic health and its connection to diseases involving oxidative stress, inflammation, and nucleic acid metabolism.

#### 3.2.2. Metabolomes Profiling Using Product Ion Fragmentation Method

Different amino acids and lipid molecules were identified using the ion fragmentation approach, which involved analyzing the fragmentation patterns of precursor ions to determine their molecular structures. This method provided detailed insights into the composition and concentration of specific metabolites, helping to accurately characterize the amino acids and lipids present in the serum samples. By comparing the experimental ion fragmentation data with established spectral libraries, we were able to identify and quantify a wide range of metabolites involved in metabolic processes affected by smoking.

##### Phenylalanine

**Figure 5:**
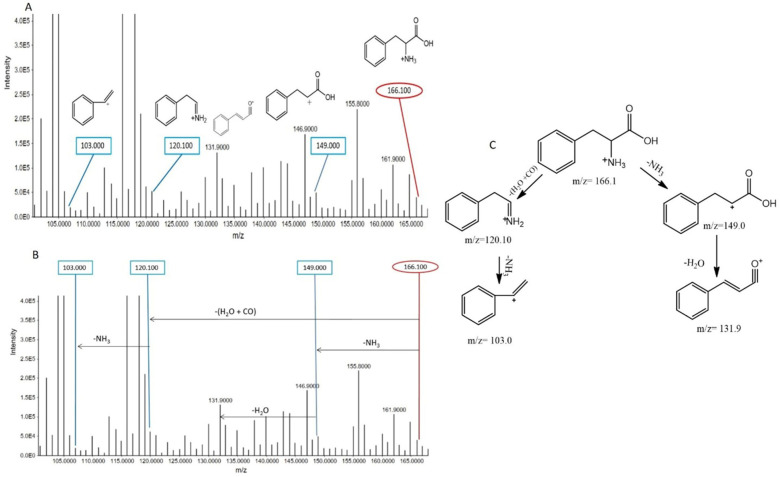
(**A**) MS/MS spectra of phenylalanine in the ESI^+^ scan mode. This spectrum emphasizes the unique peak that is characteristic of this particular metabolite at *m*/*z* 166.100. Accompanying fragment peaks were also seen, providing a thorough picture of the fragmentation pattern linked to phenylalanine at *m*/*z* 149.00, 120.100, and 103.00. (**B**) Precursor ion peaks and fragment ion peaks of phenylalanine in non-smokers. (**C**) Fragmentation pattern of phenyl alanine.

The protonated phenylalanine ion (*m*/*z* 166.100) was detected at a retention time of 4.65 min, and its fragmentation produced several distinct ion peaks. The fragmentation pattern included the removal of NH_3_ and H_2_O+CO groups, leading to the generation of fragment ions at *m*/*z* 149.000 and 120.100, respectively. Further removal of water from the 149.000 fragment resulted in the *m*/*z* 131.900 peak, while the loss of NH_3_ from the 120.100 fragment produced a peak at *m*/*z* 103.000. These specific fragmentation patterns allowed for the identification of phenylalanine. Interestingly, when comparing the intensities of these peaks between the smoker and non-smoker groups, all peak intensities were higher in the non-smoker group (Figure 5), suggesting that smoking might influence the metabolism or stability of phenylalanine in the body, potentially affecting its levels or turnover in serum.

##### Tryptophan

**Figure 6:**
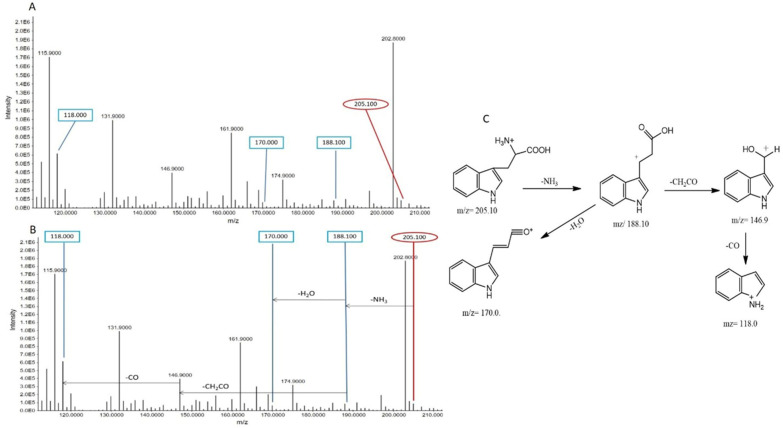
(**A**) Tryptophan’s MS/MS spectra in ESI^+^ scan mode. The peculiar peak at *m*/*z* 205.100, which is unique to this particular metabolite, is shown in this spectrum. Supporting fragment peaks are also seen, such as those at *m*/*z* 188.100, 170.000, 146.900, and 118.00, which provide a thorough understanding of the tryptophan-related fragmentation pattern. (**B**) Precursor ion peaks and fragment ion peaks of tryptophan in non-smokers. (**C**) Fragmentation pattern of tryptophan.

At a retention time (R.T.) of 3.625 min, tryptophan exhibited a precursor ion peak at *m*/*z* 205.100, corresponding to the protonated ion (C_11_H_12_N_2_O_2_^+^). The subsequent fragmentation led to the generation of a fragment ion peak at *m*/*z* 188.100 after the removal of NH_3_. Further fragmentation of the C_11_H_10_NO_2_^+^ ion resulted in a peak at *m*/*z* 146.900 after the loss of CH_2_CO, and a peak at *m*/*z* 170.000 after the removal of H_2_O. The final fragmentation step, involving the loss of CO from the ion at *m*/*z* 146.900, produced the peak at *m*/*z* 118.000 (Figure 6). These fragmentation patterns help to identify tryptophan and its metabolites. When comparing the intensities of these peaks between groups, variations may suggest changes in tryptophan metabolism due to smoking, which could potentially affect its breakdown and downstream biological pathways.

##### Valine

**Figure 7:**
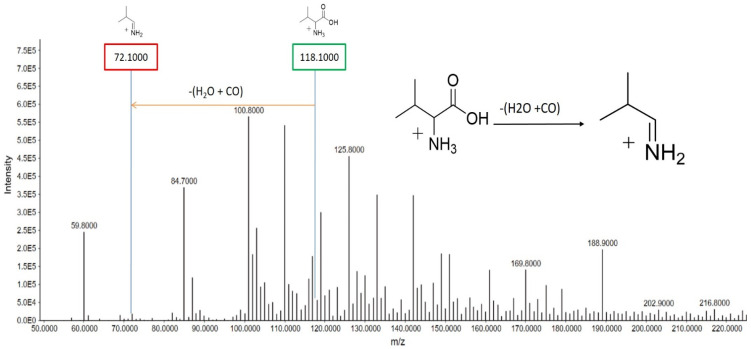
Valine’s MS/MS spectra in ESI^+^ scan mode. This spectrum emphasizes the unique peak that is characteristic of this particular metabolite at *m*/*z* 118.100. Further fragment peaks are also seen, such as those at *m*/*z* 72.100, providing a thorough understanding of the fragmentation pattern connected to valine. The precursor ion peaks and fragment ion peaks of valine and the fragmentation pattern of valine in non-smokers are represented.

At a retention time (R.T.) of 2.736 min, valine displayed a precursor ion peak at *m*/*z* 118.100, corresponding to the protonated ion (C_5_H_11_NO_2_^+^). Upon fragmentation, the removal of both H_2_O and CO resulted in the generation of a fragment ion peak at *m*/*z* 72.100 (Figure 7). This fragmentation pattern provides insight into valine’s molecular structure and its degradation products. The comparison of peak intensities between smokers and non-smokers may reveal alterations in valine metabolism related to smoking, which could impact various biological processes, including protein synthesis and energy metabolism.

##### Histidine

**Figure 8:**
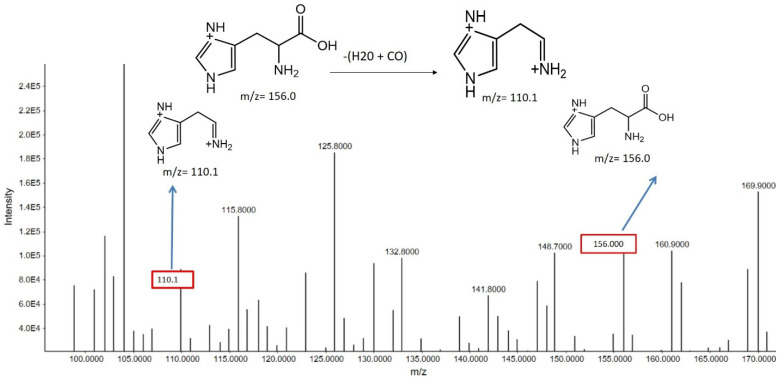
Positive mode histidine MS/MS spectra. This spectrum emphasizes the unique peak, exclusive to this particular metabolite, at *m*/*z* 156.000. Further fragment peaks are also visible, such as those at *m*/*z* 100.100, providing a thorough understanding of the fragmentation pattern connected to histidine.

Histidine exhibited a precursor ion peak at *m*/*z* 156.00, corresponding to the protonated form of the molecule (C_6_H_9_N_3_O_2_^+^). Upon fragmentation, the loss of both H_2_O and CO resulted in the generation of a fragment ion peak at *m*/*z* 110.1 (Figure 8). This fragmentation pattern suggests that histidine undergoes dehydration and decarboxylation processes during ionization and fragmentation. The changes in the fragment ion intensities, particularly when comparing smokers and non-smokers, could provide insights into how smoking impacts amino acid metabolism and associated biological functions.

##### Carnitine

**Figure 9:**
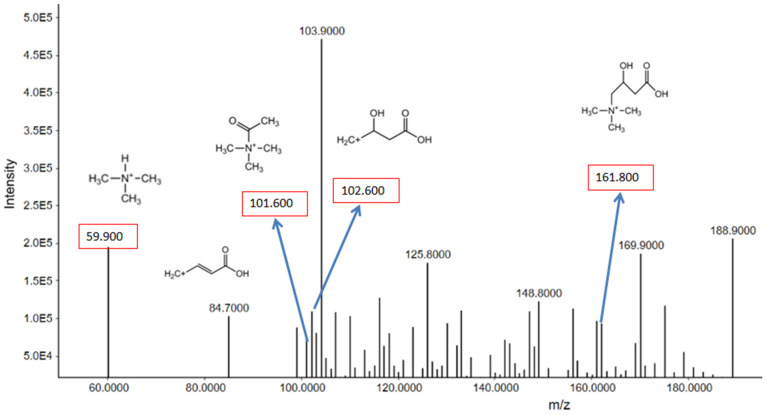
Precursor ion peaks and fragment ion peaks of carnitine in smoker group.

The [M+H]^+^ ion of carnitine, with the molecular formula C_7_H_16_NO_3_ and a molecular weight of 161.105, was observed at *m*/*z* 161.8 at a retention time of 5.952 min. Fragmentation of the carnitine molecule produces several significant peaks, including *m*/*z* 102.6, *m*/*z* 101.6, *m*/*z* 84.7, and *m*/*z* 59.9. The peak at *m*/*z* 59.9 indicates the loss of the trimethylammonium ion (C_3_H_9_N^+^) from the carnitine molecule, confirming the presence of carnitine in the sample (Figure 9). This fragmentation pattern is characteristic of carnitine and supports its identification in the sample. The analysis of carnitine levels could provide insights into metabolic changes associated with smoking, as carnitine plays a crucial role in fatty acid metabolism and mitochondrial function.

##### Sphinganine

Sphinganine, a key molecule in the sphingolipid metabolic pathway, was identified by its [M+H]^+^ ion at *m*/*z* 302.8, with a retention time of 12.83 min. The molecular weight of sphinganine is 302, and its chemical formula is C_16_H_39_NO_2_. Upon fragmentation, several notable peaks were observed: *m*/*z* 284.9—fragmentation due to the loss of one water molecule (H_2_O); *m*/*z* 266.8—further fragmentation, representing the loss of two water molecules; *m*/*z* 254.9—resulting from the loss of a CH_3_O group and an additional water molecule; *m*/*z* 175.9—fragmentation attributed to the loss of an alkyl chain with 9 carbon atoms; *m*/*z* 106.4—fragmentation due to the loss of an alkyl chain with 14 carbon atoms; and *m*/*z* 150.03—peak observed after the alkyl chain undergoes cyclization.

**Figure 10:**
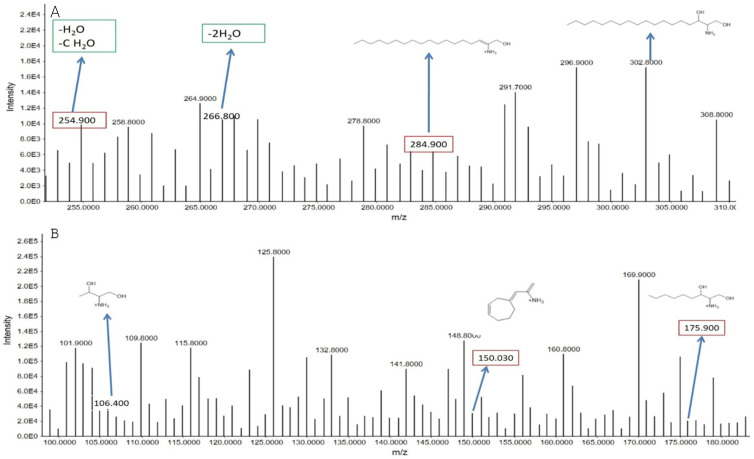
(**A**) Precursor ion peaks and the fragment ion peaks of sphinganine in smoker groups. The parent ion peak of sphinganine was identified at *m*/*z* of 302.80. The fragment ion peaks were identified at *m*/*z* of 284.90, 266.80, and 254.90. (**B**) The remaining fragment ion peaks were identified at *m*/*z* of 175.90, and 150.03, and 106.40.

These fragmentation patterns confirm the presence of sphinganine and reflect the structural characteristics of the molecule, which plays a vital role in cellular membranes and signaling. The analysis could provide insights into lipid metabolism alterations, especially in conditions like smoking, which may impact sphingolipid metabolism (Figure 10).

#### 3.2.3. Miscellaneous Amino Acids

The analysis of serum samples from smokers identified the characteristic peaks for the following amino acids: glutamic acid, glutamine, and ornithine. Glutamic acid exhibited a precursor ion peak at *m*/*z* 148.000. Glutamine was identified by its precursor ion peak at *m*/*z* 147.100. Ornithine displayed a characteristic precursor ion peak at *m*/*z* 133.100.

**Figure 11:**
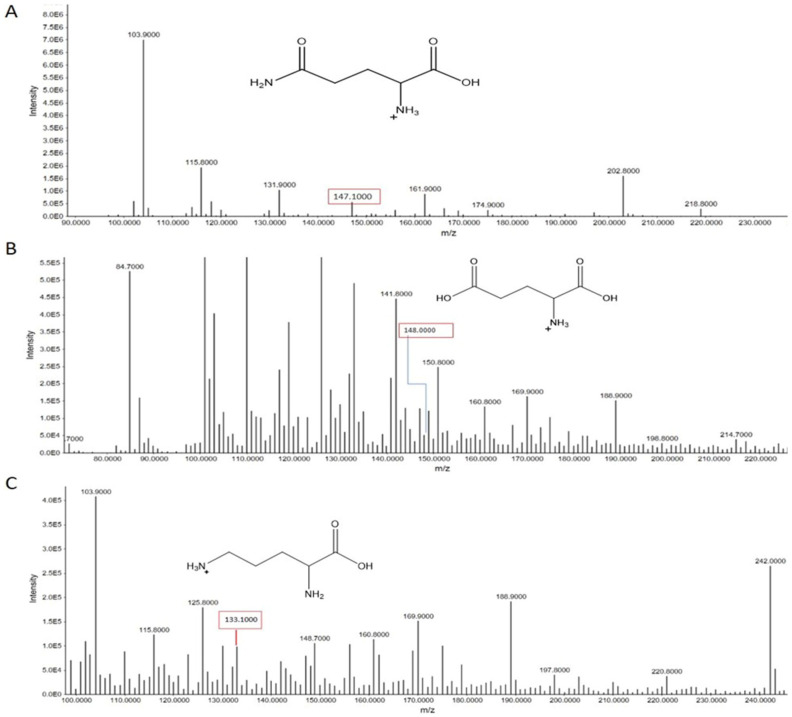
(**A**) A detailed illustration of the glutamine distinctive peak at *m*/*z* 147.100 is given. (**B**) A detailed illustration of glutamic acid distinctive peak at *m*/*z* 148.00 is provided. (**C**) The illustration also includes the ornithine distinctive peak at *m*/*z* 133.100.

These peaks, detected through advanced mass spectrometry, indicate the presence of these amino acids in the serum of smokers. Their identification suggests potential metabolic alterations linked to smoking, reflecting changes in amino acid metabolism and their roles in oxidative stress and inflammatory pathways (Figure 11).

#### 3.2.4. Differentially Detected Lipidomes

**Figure 12:**
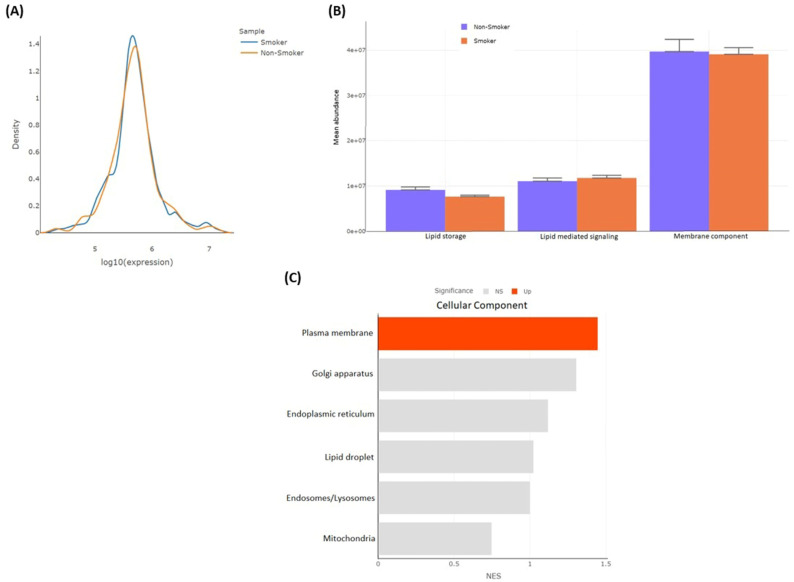
(**A**) Lipidomic analysis by density plot showing expression of lipids in smokers compared to non-smokers. (**B**) Analysis showing differential abundance of functional lipids and (**C**) plasma membrane-derived lipids compared to those from other cellular organelles.

The lipidomic comparison between smokers and non-smokers revealed significant metabolic alterations. Notably, three lipid metabolites were exclusively detected in the smoker group, while seven lipid metabolites were uniquely found in the non-smoker group (Table 4). These differences may be attributed to altered metabolic pathways in smokers or exogenous sources of lipids, warranting further investigation. In smokers, a shift toward higher lipid levels was observed, as demonstrated in the density plot (Figure 12A). Lipid-mediated signaling molecules showed a slight increase in mean abundance, suggesting a shift in cellular signaling pathways. However, certain membrane-component lipids, such as PC 36:7, SM 38:3;O3, PI 44:3, PS 42:11, SM 44:3;O2, PE 44:12, HexCer 44:2;O2, and PC 42:8, exhibited a slight decrease in total abundance in smokers by about 6% with *p*-value < 0.05 of each lipid. This indicates potential alterations in membrane composition, which could impact cellular integrity and function (Figure 12B).

Regarding lipid storage, triglycerides (e.g., TG 62:12, TG 50:0;O, TG 50:0, TG 58:2, and TG 60:14) displayed 125.3% lower abundance in smokers, while TG 50:0;O and TG 50:0 showed a positive fold change of 0.53 and 0.023, respectively. Additionally, lipid source analysis revealed that lipids derived from the plasma membrane were significantly (*p* < 0.05) more abundant than those originating from other organelles, such as the Golgi apparatus, endoplasmic reticulum, lipid droplets, endosomes/lysosomes, and mitochondria (Figure 12C).

These findings highlight the systemic effects of smoking on lipid metabolism, suggesting profound changes in lipid signaling, storage, and membrane composition. Further investigation into the pathways involved could provide deeper insights into the metabolic shifts associated with smoking.

**Figure 13:**
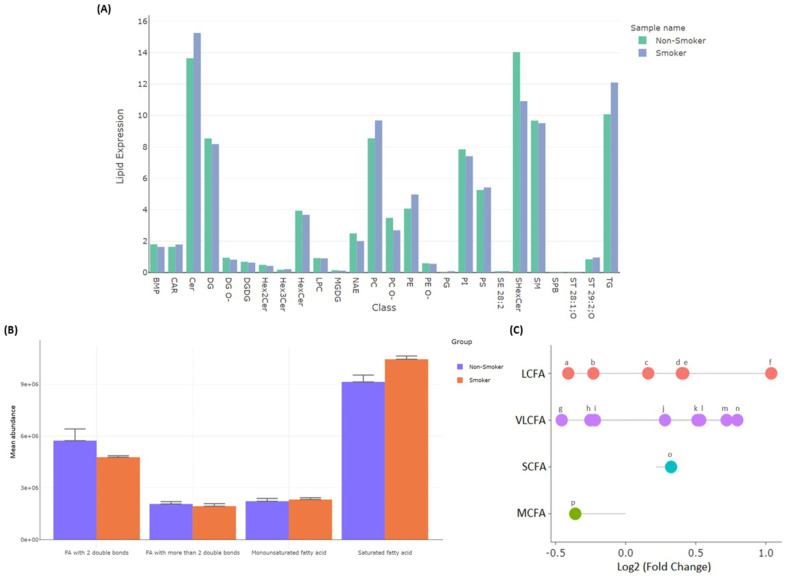
(**A**) Differential expression of lipid molecules, highlighting percentage expression among different lipid sub-classes. (**B**) Mean abundance data of fatty acids with degree of unsaturation. (**C**) Fold changes in lipids, highlighting upregulation of specific long-chain, very long chain, and short-chain fatty acids, with notable downregulation in certain medium- and long-chain fatty acids. (a) SPB 18:0; O3. (b) CAR 20:0. (c) LPC 18:1. (d) CAR 16:3. (e) CAR 21:1. (f) NAE 17:0. (g) LPC 24:3. (h) CAR 22:2. (i) NAE 23:0. (j) LPC 26:5. (k) LPS 38:4. (l) CAR 28:2. (m) CAR 27:1. (n) SPB 24:0; O2. (o) NAE 4:0. (p) CAR 7:0.

The lipidomic and fatty acid profile comparison between smokers and non-smokers revealed significant differences in lipid expression across various classes, with smokers consistently showing higher lipid levels (Figure 13A). Specifically, the expression of ceramides (Cer) was elevated in smokers (15.26%) compared to non-smokers (13.6%), while sulfatides (SHexCer) and triglycerides (TGs) were also higher in smokers, with values of 14.04% and 12.1%, respectively, compared to 10.91% and 10.08% in non-smokers. Phosphatidylcholines (PCs) showed a similar trend, with 9.7% in smokers compared to 8.5% in non-smokers. These increases suggest that smoking may cause alterations in lipid metabolism, potentially contributing to smoking-related health risks.

Regarding fatty acid composition, saturated fatty acids (SFAs) exhibited higher mean abundance in smokers, with key lipids such as SPB 24:0;O2, CAR 20:0, and NAE 4:0 accumulating more in the smoker group, with *p*-values of 0.0011219, 0.016028, and 0.003823, respectively. In contrast, fatty acids with two double bonds, such as CAR 28:2 and CAR 22:2, were reduced in smokers by 84.5%, indicating a decrease in these unsaturated lipids. Monounsaturated fatty acids (e.g., LPC 18:1 with positive fold change of 0.31 and CAR 27:1 with negative fold change of 7.7) and those with more than two double bonds (e.g., LPC 24:3 and LPC 38:4 with *p*-values of 0.0083213 and 0.002817, respectively) showed only slight differences in total abundance between the two groups (Figure 13B).

Further analysis of fatty acid chain length revealed distinct patterns of regulation. Long-chain fatty acids (LCFAs) showed upregulation in smokers, as compared to non-smokers, of specific lipids, like NAE 17:0, CAR 21:1, and LPC 18:1, with positive fold changes of 7.7, 0.2, and 0.43, respectively, while smokers’ data showed downregulation of CAR 20:0 and SBP 18:0;O3 by 20% and 94%, respectively, as compared to non-smokers. For very long chain fatty acids (VLCFAs), lipids such as SBP 24:0;O2, CAR 27:1, and LPC 26:5 were upregulated with positive fold changes of 5, 7.72, and 0.2, respectively, while NAE 23:0 and CAR 22:2 were downregulated by 78% and 91%, respectively, in smokers as compared to non-smokers. Medium-chain fatty acids (MCFAs) showed a downregulation of CAR 7:0 by 98%, and short-chain fatty acids (SCFAs) exhibited a significant upregulation of NAE 4:0 (*p* = 0.004) in the smoker group as compared to non-smokers (Figure 13C).

These findings indicate that smoking leads to substantial alterations in lipid and fatty acid metabolism, with changes in lipid classes, fatty acid saturation, and chain length. The upregulation of saturated and very long chain fatty acids, along with the downregulation of certain unsaturated lipids, suggests a complex disruption of metabolic pathways in smokers. These metabolic shifts may underlie smoking-related health issues, including inflammation, oxidative stress, and cellular dysfunction. Further research into these pathways could provide valuable insights into their clinical significance and their potential role in smoking-related diseases.

### 3.3. Effect on Serum Cd and Zn Levels

**Figure 14:**
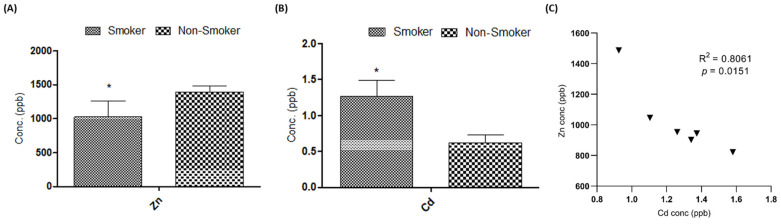
Concentration of (**A**) cadmium and (**B**) zinc in blood of smoker and non-smoker groups. (**C**) Correlation between Cd and Zn concentration in the smoker group. The results showed that there was a significant difference (*p* < 0.05) between the two groups, the smokers and the non-smokers. * represent the level of significant difference when compared to the smokers with non-smokers.

The serum concentrations of Cd and Zn were measured in both the smoker and non-smoker groups, revealing significant differences between the two. The mean Cd concentration was significantly higher in smokers (1.264 ppb) compared to non-smokers (0.624 ppb), with a *p*-value less than 0.05, indicating statistical significance (Figure 14A). In contrast, the mean Zn concentration was lower in smokers (1025.08 ppb) compared to non-smokers (1389.66 ppb) (Figure 14B).

The study further explored the relationship between Cd and Zn in the serum of smokers, revealing a strong inverse correlation. This finding suggests that Cd may reduce the concentration of Zn by competing for the same binding sites in the body. The correlation coefficient (R^2^ = 0.8061, *p* = 0.015) confirms a significant negative relationship between these two metals (Figure 14C). These results indicate that higher Cd levels in smokers correspond to lower Zn levels, emphasizing the potential impact of Cd exposure on zinc metabolism. This inverse correlation may contribute to the understanding of smoking-related alterations in metal homeostasis and their implications for health.

## 4. Discussion

This study aims to investigate the biochemical and metabolomic alterations induced by smoking, with a focus on disruptions in lipid, amino acid, and carbohydrate metabolism. To achieve this, a comprehensive analytical approach was employed, combining biochemical assays and advanced metabolomic techniques. The study involved two groups: smokers and non-smokers, with blood samples collected and analyzed using LC-MS/MS and standard biochemical assays. The results revealed significant metabolic disruptions in smokers compared to non-smokers, including altered lipid and amino acid profiles, elevated inflammatory markers (CRP and IL-6), and increased oxidative stress indicators (e.g., oxidized glutathione). A total of 343 metabolites were significantly altered, with 116 upregulated and 127 downregulated, affecting key pathways, contributing to smoking-induced metabolic disorders like diabetes and cardiovascular diseases.

The study demonstrated significantly higher FBS, HbA1c, HOMA-IR, and creatinine levels in smokers compared to non-smokers, indicating impaired glycemic control and early signs of renal dysfunction. These findings are consistent with Soulimane’s research [19], who reported that smoking exacerbates insulin resistance, reflected in elevated HOMA-IR values, and contributes to hyperglycemia by impairing insulin signaling. Similarly, another study observed higher HbA1c levels in smokers, associating them with chronic inflammation and oxidative stress, which both disrupt glucose metabolism [20]. Elevated creatinine levels in smokers, as noted in our study, align with Halimi’s study, which linked smoking-induced oxidative damage to reduced renal function [21].

Our findings indicate that smoking leads to significant shifts in lipid parameters, including elevated levels of LDL, cholesterol, and altered triglyceride concentrations, which are known risk factors for the development of metabolic diseases. These results are consistent with earlier studies that reported similar outcomes. Smoking leads to an increase in plasma levels of triglycerides, and this increase is associated with elevated amounts of lipoproteins high in triglycerides, such as LDL. This increase may result from either a reduced clearance rate of these lipoproteins or an increase in hepatic triglyceride production. Previous research by Larregle et al. (2008) also demonstrated elevated plasma triglycerides, LDL, and VLDL in smokers [22]. Moreover, the reduced HDL levels align with observations by Nakamura, emphasizing smoking’s role in cardiovascular risk. Unique to our study, the identification of sphinganine and specific ceramides points to disruptions in sphingolipid metabolism that have been linked to insulin resistance and atherosclerosis, extending the understanding of smoking-induced lipid dysregulation.

Elevated BUN and creatinine levels in the serum of the smoking group may contribute to the development of renal disease [23]. In our study, the mean BUN values for the smoking and non-smoker groups were 35.285 ± 5.645 mg/dL and 32.642 ± 5.534 mg/dL, respectively. Similarly, the mean creatinine values were 0.959 ± 0.197 mg/dL and 0.873 ± 0.143 mg/dL, respectively. Our findings indicate a strong positive association of smoking with elevated serum creatinine and BUN levels. Elevated urea levels have been linked to an increased likelihood of insulin resistance and reduced insulin production, contributing to excessively high blood glucose levels [24]. These findings align with the results of Huang et al. (2016), who reported similar elevations in BUN and creatinine levels among smokers, attributing these changes to the nephrotoxic effects of smoking-induced oxidative stress and inflammation [25]. Similarly, Fu et al. (2022) demonstrated that chronic exposure to cigarette smoke is associated with impaired glomerular function, leading to higher creatinine levels. The strong positive correlation between BUN and creatinine in our study further supports the association of smoking with early markers of renal damage, consistent with previous research linking smoking to chronic kidney disease progression [26].

In our findings, smokers exhibited lower levels of ALT and AST compared to non-smokers. Elevated levels of liver function indicators such as ALT and AST have been associated with nicotine in cigarette smoke across the US population [27], a finding which is consistent with our findings. Although the exact mechanism behind smoking’s harmful effects on the liver is not fully understood, oxidative stress and inflammation are recognized as major contributors. Nicotine exposure promotes the infiltration of Kupffer cells and polymorphonuclear neutrophils into the liver [28].

Our findings of elevated CRP and IL-6 in smokers are consistent with studies by Aldaham (2015) that reported similar increases in inflammatory markers associated with smoking-induced oxidative stress [29]. Similarly, the downregulation of biliverdin reflects reduced antioxidant capacity, as previously noted by Paul (2024). The observed metabolic disruptions in lipid and amino acid metabolism can be attributed, in part, to smoking-induced oxidative stress and chronic inflammation, as evidenced by elevated levels of CRP, IL-6, and oxidized glutathione in smokers [30]. Oxidative stress disrupts cellular antioxidant defenses, leading to lipid peroxidation and amino acid imbalances, while inflammation promotes hepatic fatty acid synthesis and amino acid turnover. These mechanisms highlight the pivotal role of oxidative and inflammatory pathways in driving smoking-related metabolic dysfunctions [31,32].

Smokers show a significant increase in Cd concentration as tobacco plants absorb cadmium from the soil. Previous studies indicate that the kidneys are particularly vulnerable to Cd toxicity due to their inefficient mechanism for eliminating Cd, especially in the glomeruli and proximal tubules. As a result, chronic Cd exposure leads to the accumulation of about 50% of the body’s total Cd in the kidneys, potentially causing severe and even life-threatening effects [33]. In this study, we also observed a correlation between Cd and essential metals like Zn, which was found to be inversely related. This inverse relationship may be because Cd and Zn interact with overlapping sites within biological systems due to their similar chemical properties, but their binding affinities and effects differ. Zinc typically binds to sites involving cysteine residues and thiol groups, playing crucial roles in enzyme function and immune response through its presence in zinc fingers and other proteins [34,35]. On the other hand, Cd, which can mimic zinc due to its similar size and charge, binds to sulfhydryl groups with higher affinity, often displacing zinc and leading to toxicity [36,37,38]. These combined effects can contribute to various health issues, including bone diseases, altered muscle function, and impaired cellular signaling [37].

Several metabolites identified in our study exhibit significant alterations and are implicated in metabolic disorders, including diabetes. The upregulation of kynurenine and 3-hydroxykynurenine in smokers, suggesting a disruption in the kynurenine pathway, aligns with findings from Yang (2024), who associated elevated kynurenine metabolites with cardiovascular disorders [39]. Similarly, the observed increase in uric acid and hypoxanthine levels reflects heightened purine metabolism, consistent with Gherghina (2022), who linked these metabolites to oxidative stress and metabolic dysfunction in smokers [40]. Elevated beta-tocopherol levels, indicating a compensatory antioxidant response, parallel findings from Bruno (2005), who reported similar trends in smokers experiencing oxidative stress. These comparisons highlight the consistency of our findings with the existing literature while providing new insights into the systemic impact of smoking-induced metabolic disruptions.

In our study, purine and its catabolic intermediate, hypoxanthine, were significantly elevated in smokers, reflecting heightened purine catabolism, which contributes to oxidative stress through reactive oxygen species production. Additionally, smokers exhibited lower carnosine levels, consistent with observations in passive smokers, likely due to decreased histidine availability for synthesis. Reduced carnosine levels may signify oxidative stress and inflammation, leading to increased utilization [41]. Elevated 7-methylguanine levels were observed in our study in smokers, potentially caused by nicotine-derived nitrosamine ketones (NNKs) and DNA damage repair, thus further indicating exposure to methylating agents [42]. These findings support the idea that 7-methylguanine serves as a biomarker for DNA damage and methylation exposure in smokers.

Furthermore, we observed the upregulation of several amino acids, including arginine, glutamic acid, leucine, tryptophan, valine, and cysteine; and downregulation of histidine and methionine, all of which can contribute to various metabolic disorders due to their roles in critical biochemical pathways. For instance, excess arginine may overwhelm the urea cycle, leading to hyperammonemia and ammonia accumulation in the blood, which can be toxic [43]. High levels of glutamic acid can induce excitotoxicity, damaging nerve cells through excessive stimulation [44]. Additionally, excess cysteine can contribute to cystinuria, leading to cystine stone formation in the kidneys that can cause recurrent kidney stones and potential damage [45]. Essential amino acids such as tryptophan and phenylalanine have been linked to insulin resistance, increasing the risk of type 2 diabetes [46].

In this study, we found an increase in tryptophan levels, accompanied by lower concentrations of quinolinic acid and pyridoxal 5-phosphate (P5P). In this study, we observed increased tryptophan levels along with decreased concentrations of quinolinic acid and pyridoxal 5-phosphate (P5P). Our findings on P5P levels align with previous research by Okafor et al. (2018) [47], but they contrast with results on quinolinic acid by Onmaz et al. (2025) [48], as the level of quinolinic acid is lower in our study. Quinolinic acid is an inhibitor of gluconeogenesis and phosphoenolpyruvate carboxykinase, contributing to hyperglycemia in smokers. Changes in the kynurenine pathway, especially the imbalance between kynurenine, 3-hydroxykynurenine, and quinolinic acid, suggest a potential mechanism by which smoking may contribute to metabolic dysfunction through dysregulation of this pathway [49].

**Figure 15:**
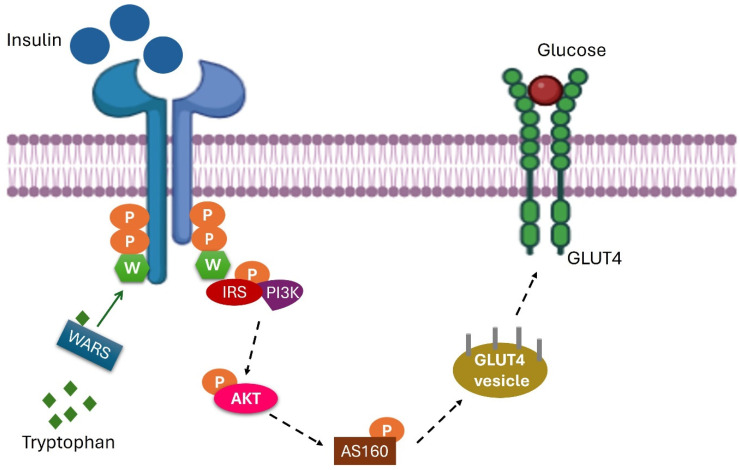
Increase in tryptophan concentration is linked to insulin resistance.

Previous research also indicates that individuals with incident type 2 diabetes have significantly higher levels of tryptophan compared to controls. Tryptophan can be detected by WARS (tryptophan–tRNA synthetase), which attaches tryptophan to the ε-amines of lysine residues on the insulin receptor. This modification interferes with insulin receptor phosphorylation, reducing insulin-signaling sensitivity and contributing to insulin resistance [50] as shown in Figure 15.

The upregulation of carnitine, sphinganine, lysophosphatidylcholine (LPC 11:0), and specific ceramides can contribute to various metabolic disorders due to their pivotal roles in lipid metabolism and cellular functions. For example, carnitine is essential for transporting long-chain fatty acids into mitochondria for beta-oxidation. Disruptions in the carnitine cycle can lead to Fatty Acid Oxidation Disorders (FAODs), which result in symptoms such as muscle weakness, cardiomyopathy, and liver dysfunction [51]. Sphinganine, a precursor in the synthesis of complex sphingolipids, can accumulate in conditions where metabolic defects are present. This accumulation may cause sphingolipidoses, which disrupt cellular functions and lead to neurodegeneration and organomegaly [52]. The dysregulation of these metabolites emphasizes their critical involvement in maintaining normal cellular homeostasis and their potential role in metabolic disease development.

**Figure 16:**
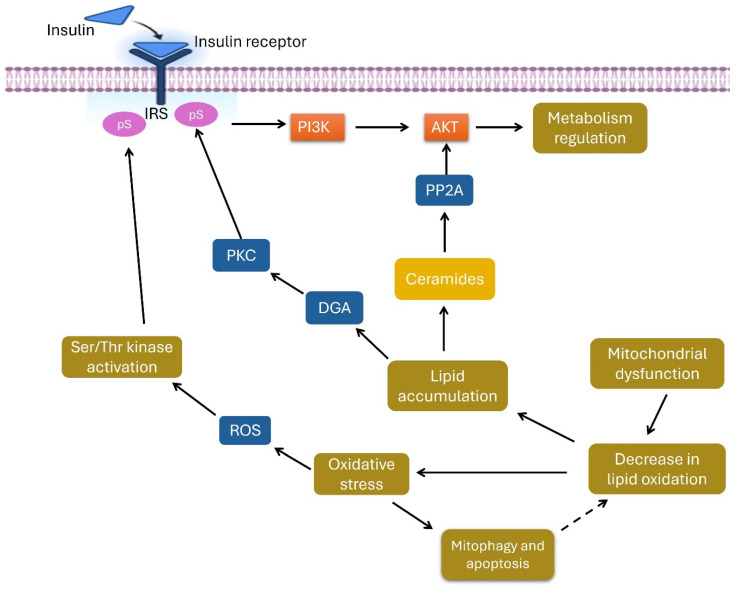
Lipid dysregulation triggers the activation of PKC and Ser/Thr kinases, leading to increased insulin resistance.

Altered levels of lysophosphatidylcholines (LPCs), particularly LPC 11:0, are associated with T2DM, as they contribute to insulin resistance and inflammation through their effects on lipid signaling pathways [53]. Nicotine-induced stress on mitochondria decreases lipid oxidation and promotes lipid accumulation, which subsequently activates protein kinase C (PKC) and Ser/Thr kinases. This activation contributes to increased insulin resistance in smokers [54]. Additionally, elevated ceramide levels (e.g., Cer 49:3;O4|Cer and 13:1;O3/36:2(2OH)) are linked to metabolic syndrome and cardiovascular diseases by promoting insulin resistance, inflammation, and atherosclerosis [55]. These metabolic disorders illustrate the critical roles these molecules play in cellular metabolism and signaling, with disruptions leading to significant health issues (Figure 16).

Metabolomics aids in identifying specific biomarkers related to the onset or progression of diseases. These biomarkers can often be detected early, sometimes even before clinical symptoms manifest, facilitating earlier diagnosis. In summary, metabolomics enhances early disease detection, allowing for prompt interventions and improved patient outcomes.

## 5. Limitations

This study has several limitations that should be acknowledged. First, potential confounding factors such as diet, alcohol consumption, physical activity, and exposure to environmental toxins were not thoroughly controlled or reported, which may have influenced the observed metabolic alterations. Additionally, the cross-sectional design of the study limits the ability to establish causal relationships between smoking and metabolic changes. While LC-MS/MS-based metabolomic profiling was employed, the inclusion of other omics approaches, such as proteomics and genomics, could provide a more comprehensive understanding of the molecular mechanisms involved. Lastly, the study population was limited in diversity, which may restrict the generalizability of the findings to broader populations.

## 6. Conclusions

This study provides a comprehensive analysis of the metabolic and biochemical alterations induced by smoking, with a focus on the impact of nicotine exposure on lipid metabolism, amino acid profiles, inflammation, and oxidative stress. Our findings highlight significant disruptions in lipid profiles, including elevated triglycerides, LDL, and total cholesterol levels, alongside reduced HDL concentrations in smokers. These lipid imbalances suggest an increased risk of cardiovascular diseases. Additionally, the upregulation of amino acids such as arginine, glutamic acid, tryptophan, and histidine was associated with insulin resistance, hyperglycemia, and other metabolic disorders, offering insight into the mechanisms driving smoking-induced metabolic dysfunction. Furthermore, we observed significant elevation of inflammatory markers such as CRP and IL-6, indicating chronic inflammation as a contributor to hepatic lipid accumulation and liver damage. Alterations in essential metal concentrations, such as the inverse relationship between Cd and Zn, further elucidated the toxic effects of smoking on renal and cellular function. Notably, disruptions in the kynurenine pathway, particularly the imbalance of tryptophan metabolites, also suggested a potential mechanism for smoking-induced metabolic dysfunction. The metabolomic approach employed in this study has provided valuable insights into the early biomarkers and molecular pathways associated with smoking-related metabolic disturbances. By identifying these alterations, we not only enhance our understanding of the underlying mechanisms but also pave the way for the development of early diagnostic tools and potential therapeutic targets for smoking-induced diseases.

Future research should focus on the longitudinal assessment of smoking-induced metabolic changes to further investigate the progression of these alterations and their long-term impact on health. Additionally, exploring potential interventions, such as dietary modifications or pharmacological agents, to counteract smoking-induced metabolic dysfunctions could provide valuable therapeutic strategies. A deeper investigation into the role of specific metabolites, such as those involved in the kynurenine pathway, could yield novel insights into personalized treatment options for metabolic disorders linked to smoking.

## Figures and Tables

**Table 1:** Instrument parameters for LC-MS/MS.

Parameters	Particulars
Instrument	The triple quadrupole LC/MS 6495C from Agilent accompanied by electrospray ionization source
Mode of ion	Positive ion scan mode
Sheath gas temperature	300 °C
Sheath gas flow rate	8 L/min
Range of scanning mass	50 to 1000 *m*/*z*
Flow rate of auxiliary gas	14/min
Capillary voltage	3000 V
Nozzle voltage	1500 V

**Table 2:** Blood levels of biochemical biomarkers of smokers and non-smokers. The superscript “a” shows the significant difference between the two groups, smokers and non-smokers.

Biochemical Parameters	Non-Smoker	Smoker	*p*-Value
**Glycemic index markers**			
FBG (mg/dL)	84.000 ± 14.004	93.000 ± 9.785	<0.05 ^a^
HbA1c (%)	4.235 ± 0.885	4.876 ± 0.762	<0.05 ^a^
Insulin (U/mL)	9.788 ± 2.501	11.9 ± 2.635	<0.05 ^a^
**Lipid profile biomarkers**			
Cholesterol (mg/dL)	128.666 ± 28.854	154.888 ± 35.565	<0.05 ^a^
LDL (mg/dL)	92.363 ± 16.842	117.545 ± 24.138	<0.05 ^a^
HDL (mg/dL)	70.363 ± 9.08	57.181 ± 12.440	<0.05 ^a^
Triglycerides (mg/dL)	120.478 ± 28.348	153.043 ± 43.347	<0.05 ^a^
**Inflammatory biomarkers**			
CRP (mg/L)	4.2 ± 0.618	4.937 ± 0.568	<0.05 ^a^
IL-6 (pg/mL)	5.21 ± 0.769	6.137± 0.979	<0.05 ^a^
**Liver function biomarkers**			
ALT (U/L)	25.919 ± 10.011	30.112 ± 11.512	<0.05 ^a^
AST (U/L)	25.758 ± 7.049	28.93 ± 8.853	<0.05 ^a^
ALP (U/L)	83.677 ± 23.327	93.806 ± 21.350	<0.05 ^a^
**Kidney function biomarkers**			
Creatinine (mg/dL)	0.873 ± 0.143	0.959 ± 0.197	<0.05 ^a^
BUN (mg/dL)	32.642 ± 5.534	35.285 ± 5.645	<0.05 ^a^

A two-tailed Student’s *t*-test was used to perform a statistical comparison between two columns. *p*-values less than 0.05 were regarded as significant. The information is displayed as mean ± SD.

**Table 3:** Via enrichment analysis, it was observed that significant number of metabolites of different metabolic processes were identified, so chances of perturbation of these processes are greater compared to others. The top 25 metabolic processes are illustrated, along with *p*-values and enrichment ratio. The superscript “a” shows the significant difference between the two groups including the smokers and non-smokers, while the superscript “b” indicates the non-significant difference between the two groups.

Pathway Name	Total	Expected	Hits	*p*-Value
Lysine degradation	30	0.546	3	0.0158 ^a^
Cysteine and methionine metabolism	33	0.6	3	0.0204 ^a^
Arginine biosynthesis	14	0.255	2	0.0254 ^a^
Butanoate metabolism	15	0.273	2	0.029 ^a^
Purine metabolism	70	1.27	4	0.0352 ^a^
Tryptophan metabolism	41	0.746	3	0.0362 ^a^
Amino sugar and nucleotide sugar metabolism	42	0.764	3	0.0385 ^a^
beta-Alanine metabolism	21	0.382	2	0.0542 ^a^
Alanine, aspartate and glutamate metabolism	28	0.509	2	0.0902 ^b^
Glycine, serine and threonine metabolism	33	0.6	2	0.119 ^b^
Thiamine metabolism	7	0.127	1	0.121 ^b^
Arginine and proline metabolism	36	0.655	2	0.138 ^b^
Biotin metabolism	10	0.182	1	0.168 ^b^
Steroid biosynthesis	41	0.746	2	0.17 ^b^
Histidine metabolism	16	0.291	1	0.256 ^b^
Terpenoid backbone biosynthesis	18	0.327	1	0.283 ^b^
Citrate cycle (TCA cycle)	20	0.364	1	0.309 ^b^
Ether lipid metabolism	20	0.364	1	0.309 ^b^
Propanoate metabolism	21	0.382	1	0.322 ^b^
Pyruvate metabolism	23	0.418	1	0.346 ^b^
Glycolysis/Gluconeogenesis	26	0.473	1	0.382 ^b^
Galactose metabolism	27	0.491	1	0.393 ^b^
Glutathione metabolism	28	0.509	1	0.405 ^b^
Lipoic acid metabolism	28	0.509	1	0.405 ^b^
Inositol phosphate metabolism	30	0.546	1	0.427 ^b^

**Table 4:** Differentially detected Metabolites in MS/MS spectra of non-smokers and smokers.

**Differentially Detected Metabolites in MS/MS Spectra of Non-Smokers**				
**Sr. No**	**R.T.**	***m*** **/*z***	**Metabolite**	**Area**
1	8.736	259.2	NAOrn 8:0	11,496
2	8.091	270.2	NAGly 13:1	12,542
3	4.065	275.2	NAOrn 8:0;O	423,486
4	5.347	278.2	DG 10:0|DG 5:0_5:0	12,878
5	6.456	325.2	DG 13:0	14,618
6	14.555	758.7	Cer49:3;O4|Cer,13:1;O3/36:2(2OH)	1,840,580
7	2.304	948.9	TG 57:1|TG 8:0_11:0_38:1	105,025
**Differentially Detected Metabolites in MS/MS Spectra of Smokers**				
**Sr. No**	**R.T**	***m*** **/*z***	**Metabolite**	**Area**
1	3.334	330.3	SPB 19:1;O3	24,725
2	3.9	355.3	NAGly 18:2	7024
3	9.286	358.3	CAR 13:0	15,329

## Data Availability

All data are available in this manuscript and its Supplementary File.

## Supplementary Materials

The following supporting information can be downloaded at https://www.mdpi.com/article/10.3390/metabo15020096/s1. Table S1: Annotated metabolites with mean intensities in smokers and non-smokers; Table S2: Significantly altered metabolites (*p* < 0.05); Table S3: Significantly altered metabolites by fold-change analysis; Table S4: Significantly altered metabolites by *p*-value and fold-change analysis; Table S5: Enriched diseases identified as significant (*p*-value was less than 0.05).

## References

[1] Benowitz N.L. (2010). Nicotine Addiction. N. Engl. J. Med..

[2] Fiore M.C., Jaén C., Baker T.B., Bailey W.C., Benowitz N.L., Curry S.J., Dorfman S.F., Froelicher E., Goldstein M. (2008). A Clinical Practice Guideline for Treating Tobacco Use and Dependence: 2008 Update: A US public health service report. Am. J. Prev. Med..

[3] Lerman C., Tyndale R., Patterson F., Wileyto E.P., Shields P.G., Pinto A., Benowitz N. (2006). Nicotine metabolite ratio predicts efficacy of transdermal nicotine for smoking cessation. Clin. Pharmacol. Ther..

[4] Tan X., Vrana K., Ding Z.-M. (2021). Cotinine: Pharmacologically active metabolite of nicotine and neural mechanisms for its actions. Front. Behav. Neurosci..

[5] Shoaib S.M., Afzal S., Feezan A., Akash M.S.H., Nadeem A., Mir T.M. (2023). Metabolomics Analysis and Biochemical Profiling of Arsenic-Induced Metabolic Impairment and Disease Susceptibility. Biomolecules.

[6] Fan J., Zhou Y., Meng R., Tang J., Zhu J., Aldrich M.C., Cox N.J., Zhu Y., Li Y., Zhou D. (2023). Cross-talks between gut microbiota and tobacco smoking: A two-sample Mendelian randomization study. BMC Med..

[7] Bajaj M. (2012). Nicotine and Insulin Resistance: When the Smoke Clears. Diabetes.

[8] Ahmadkhaniha R., Yousefian F., Rastkari N. (2021). Impact of smoking on oxidant/antioxidant status and oxidative stress index levels in serum of the university students. J. Environ. Health Sci. Eng..

[9] Chen B., Zeng G., Sun L., Jiang C. (2024). When smoke meets gut: Deciphering the interactions between tobacco smoking and gut microbiota in disease development. Sci. China Life Sci..

[10] Shen Y., Wang P., Yang X., Chen M., Dong Y., Li J. (2023). Untargeted metabolomics unravel serum metabolic alterations in smokers with hypertension. Front. Physiol..

[11] Harada S., Ohmomo H., Matsumoto M., Sata M., Iida M., Hirata A., Miyagawa N., Kuwabara K., Kato S., Toki R. (2023). Metabolomics Profiles Alterations in Cigarette Smokers and Heated Tobacco Product Users. J. Epidemiol..

[12] Ganguly K., Levänen B., Palmberg L., Åkesson A., Lindén A. (2018). Cadmium in tobacco smokers: A neglected link to lung disease?. Eur. Respir. Rev..

[13] Tkachenko K. (2023). Multiplatform Metabolome Profiling to Identify Specific Signatures and Biomarkers in Blood Samples: Untargeted Approach. Ph.D. Thesis.

[14] Dator R., Villalta P.W., Thomson N., Jensen J., Hatsukami D.K., Stepanov I., Warth B., Balbo S. (2020). Metabolomics profiles of smokers from two ethnic groups with differing lung cancer risk. Chem. Res. Toxicol..

[15] Novelle M.G., Wahl D., Diéguez C., Bernier M., de Cabo R. (2015). Resveratrol supplementation: Where are we now and where should we go?. Ageing Res. Rev..

[16] Saba S., Akash M.S.H., Rehman K., Saleem U., Fiayyaz F., Ahmad T. (2020). Assessment of heavy metals by ICP-OES and their impact on insulin stimulating hormone and carbohydrate metabolizing enzymes. Clin. Exp. Pharmacol. Physiol..

[17] Zhang R., Sun X., Huang Z., Pan Y., Westbrook A., Li S., Bazzano L., Chen W., He J., Kelly T. (2022). Examination of serum metabolome altered by cigarette smoking identifies novel metabolites mediating smoking-BMI association. Obesity.

[18] Wang Q., Ji X., Rahman I. (2021). Dysregulated Metabolites Serve as Novel Biomarkers for Metabolic Diseases Caused by E-Cigarette Vaping and Cigarette Smoking. Metabolites.

[19] Soulimane S., Simon D., Herman W.H., Lange C., Lee C.M., Colagiuri S., Shaw J.E., Zimmet P.Z., Magliano D., Ferreira S.R. (2014). HbA1c, fasting and 2 h plasma glucose in current, ex- and never-smokers: A meta-analysis. Diabetologia.

[20] Vlassopoulos A., Lean M.E., Combet E. (2013). Influence of smoking and diet on glycated haemoglobin and ‘pre-diabetes’ categorisation: A cross-sectional analysis. BMC Public Health.

[21] Halimi J.M., Giraudeau B., Vol S., Cacès E., Nivet H., Lebranchu Y., Tichet J. (2000). Effects of current smoking and smoking discontinuation on renal function and proteinuria in the general population. Kidney Int..

[22] Larregle E.V., Varas S.M., Oliveros L.B., Martinez L.D., Antón R., Marchevsky E., Giménez M.S. (2008). Lipid metabolism in liver of rat exposed to cadmium. Food Chem. Toxicol..

[23] Cheng Y.-Y., Huang N.-C., Chang Y.-T., Sung J.-M., Shen K.-H., Tsai C.-C., Guo H.-R. (2017). Associations between arsenic in drinking water and the progression of chronic kidney disease: A nationwide study in Taiwan. J. Hazard. Mater..

[24] Xie Y., Bowe B., Li T., Xian H., Al-Aly Z. (2018). Blood urea nitrogen and risk of insulin use among people with diabetes. Diabetes Vasc. Dis. Res..

[25] Huang F., Chen J., Liu X., Han F., Cai Q., Peng G., Zhang K., Chen W., Wang J., Huang H. (2016). Cigarette smoking reduced renal function deterioration in hypertensive patients may be mediated by elevated homocysteine. Oncotarget.

[26] Fu Y.C., Xu Z.L., Zhao M.Y., Xu K. (2022). The association between smoking and renal function in people over 20 years old. Front. Med..

[27] Hyder O., Chung M., Cosgrove D., Herman J.M., Li Z., Firoozmand A., Gurakar A., Koteish A., Pawlik T.M. (2013). Cadmium exposure and liver disease among US adults. J. Gastrointest. Surg..

[28] Hong D., Min J.Y., Min K.B. (2021). Association Between Cadmium Exposure and Liver Function in Adults in the United States: A Cross-sectional Study. J. Prev. Med. Public Health.

[29] Aldaham S., Foote J.A., Chow H.H., Hakim I.A. (2015). Smoking Status Effect on Inflammatory Markers in a Randomized Trial of Current and Former Heavy Smokers. Int. J. Inflam..

[30] Caliri A.W., Tommasi S., Besaratinia A. (2021). Relationships among smoking, oxidative stress, inflammation, macromolecular damage, and cancer. Mutat. Res. Rev. Mutat. Res..

[31] Chen Z., Tian R., She Z., Cai J., Li H. (2020). Role of oxidative stress in the pathogenesis of nonalcoholic fatty liver disease. Free. Radic. Biol. Med..

[32] Vona R., Gambardella L., Cittadini C., Straface E., Pietraforte D. (2019). Biomarkers of oxidative stress in metabolic syndrome and associated diseases. Oxidative Med. Cell. Longev..

[33] Satarug S. (2024). Is Chronic Kidney Disease Due to Cadmium Exposure Inevitable and Can It Be Reversed?. Biomedicines.

[34] Mosna K., Jurczak K., Krężel A. (2023). Differentiated Zn (II) binding affinities in animal, plant, and bacterial metallothioneins define their zinc buffering capacity at physiological pZn. Metallomics.

[35] Grzybowska E.A. (2018). Calcium-binding proteins with disordered structure and their role in secretion, storage, and cellular signaling. Biomolecules.

[36] Yu H.-t., Zhen J., Leng J.-y., Cai L., Ji H.-l., Keller B.B. (2021). Zinc as a countermeasure for cadmium toxicity. Acta Pharmacol. Sin..

[37] Choong G., Liu Y., Templeton D.M. (2014). Interplay of calcium and cadmium in mediating cadmium toxicity. Chem.-Biol. Interact..

[38] Zhou X., Hao W., Shi H., Hou Y., Xu Q. (2015). Calcium homeostasis disruption-a bridge connecting cadmium-induced apoptosis, autophagy and tumorigenesis. Oncol. Res. Treat..

[39] Yang Y., Liu X., Liu X., Xie C., Shi J. (2024). The role of the kynurenine pathway in cardiovascular disease. Front. Cardiovasc. Med..

[40] Gherghina M.E., Peride I., Tiglis M., Neagu T.P., Niculae A., Checherita I.A. (2022). Uric Acid and Oxidative Stress-Relationship with Cardiovascular, Metabolic, and Renal Impairment. Int. J. Mol. Sci..

[41] Prokopieva V.D., Yarygina E.G., Bokhan N.A., Ivanova S.A. (2016). Use of Carnosine for Oxidative Stress Reduction in Different Pathologies. Oxid. Med. Cell. Longev..

[42] Xue J., Yang S., Seng S. (2014). Mechanisms of Cancer Induction by Tobacco-Specific NNK and NNN. Cancers.

[43] Meier C., Burns K., Manolikos C., Fatovich D., Bell D.A. (2024). Hyperammonaemia: Review of the pathophysiology, aetiology and investigation. Pathology.

[44] Balkhi H.M., Gul T., Banday M.Z., Haq E. (2014). Glutamate excitotoxicity: An insight into the mechanism. Int. J. Adv. Res..

[45] D’Ambrosio V., Capolongo G., Goldfarb D., Gambaro G., Ferraro P.M. (2022). Cystinuria: An update on pathophysiology, genetics, and clinical management. Pediatr. Nephrol..

[46] Lu Y., Wang Y., Liang X., Zou L., Ong C.N., Yuan J.-M., Koh W.-P., Pan A. (2019). Serum amino acids in association with prevalent and incident type 2 diabetes in a Chinese population. Metabolites.

[47] Onmaz M., Eryavuz Onmaz D., Demirbas N., Kutlu R., Ünlü A., Hatir A. (2025). Tobacco induces abnormal metabolism of tryptophan via the kynurenine pathway. Turk. J. Biochem..

[48] Okafor I., Uzoma G., Nvani L. (2018). Cobalamin, Folate and Pyridoxal 5- Phosphate Level of Smokers in Calabar Cross River State. J. Glob. Oncol..

[49] Kozieł K., Urbanska E.M. (2023). Kynurenine pathway in diabetes mellitus—Novel pharmacological target?. Cells.

[50] Sun W.-X., Zhang K.-H., Zhou Q., Hu S.-H., Lin Y., Xu W., Zhao S.-M., Yuan Y.-Y. (2024). Tryptophanylation of insulin receptor by WARS attenuates insulin signaling. Cell. Mol. Life Sci..

[51] Longo N., Frigeni M., Pasquali M. (2016). Carnitine transport and fatty acid oxidation. Biochim. Biophys. Acta (BBA)-Mol. Cell Res..

[52] Quinville B.M., Deschenes N.M., Ryckman A.E., Walia J.S. (2021). A comprehensive review: Sphingolipid metabolism and implications of disruption in sphingolipid homeostasis. Int. J. Mol. Sci..

[53] Shao F., Hu X., Li J., Bai B., Tian L. (2023). Lipidomics analysis of impaired glucose tolerance and type 2 diabetes mellitus in overweight or obese elderly adults. Endocr. Connect..

[54] Black H.S. (2024). Oxidative Stress and ROS Link Diabetes and Cancer. J. Mol. Pathol..

[55] Zardini Buzatto A., Tatlay J., Bajwa B., Mung D., Camicioli R., Dixon R.A., Li L. (2021). Comprehensive serum lipidomics for detecting incipient dementia in Parkinson’s disease. J. Proteome Res..

