# Supplementary material for: Metabolomic Insights into Smoking-Induced Metabolic Dysfunctions: A Comprehensive Analysis of Lipid and Amino Acid Metabolomes
Source: Metabolites. 2025 Feb 4;15(2):96. doi: 10.3390/metabo15020096 (PMC11857658; doi:10.3390/metabo15020096)
Supplement: Supplementary file 1 [file metabolites-15-00096-s001.zip › metabolites-3407777-supplementary.pdf]

Table S1: Annotated metabolites with mean intensities in Smokers and Non-Smokers.

HMDB ID	Name	Retention Time (min)	Precursor m/z	Mean Intensity Smokers	Mean Intensity Non-Smokers
HMDB0000011	3-Hydroxybutyric acid	3.814	105.1	2527895.667	1218970
HMDB0000064	Creatine	3.208	392.20001	160106	163442.6667
HMDB0000172	Isoleucine	3.601	132.10001	13177762.67	9217122
HMDB0000159	Phenylalanine	13.179	166.10001	131648.3333	120362.6667
HMDB0000883	Valine	2.736	118.1	653932.3333	412938.3333
HMDB0034188	Glabridin	6.825	325	15566	15705.66667
HMDB0029531	Glabrol	3.46	393	1422815.667	1038579
NA	1-(9Z-octadecenoyl)-sn-glycero-3-phosphoserine	3.46	524.29999	6292012	6214420
NA	1,2-diheptadecanoyl-sn-glycero-3-phosphocholine	12.864	762.59998	1113472	1069053
HMDB0000593	1,2-Dioleoyl-sn-glycero-3-phosphocholine	10.788	786.59998	540407	390652.3333
NA	1,2-Dipalmitoyl-rac-glycerol	7.698	551.5	282398	533906.3333
HMDB0029279	1,3-Dicaffeoylquinic acid	12.212	515	280629.3333	260339.6667
HMDB0001926	Ethinyl estradiol	5.834	279.39999	522125.6667	537893.3333
HMDB0001491	Pyridoxal 5-phosphate	3.853	247	350287.6667	454722.3333
HMDB0003331	1-Methyladenosine	5.897	282.10001	168558	99887
NA	1-octadecanoyl-rac-glycerol	3.751	381.29999	315754.3333	300917.6667
NA	1-O-hexadecyl-2-C-methyl-3-phosphatidylcholine	11.378	496	196872.3333	148654.6667
NA	1-Palmitoylglycerophosphocholine	3.53	496.39999	2720805.667	2649906.333
NA	1-Stearoylglycerophosphocholine	3.46	524.40002	11296654.67	9274646
NA	24-Ethylcoprostanol	5.874	399.5	173939.3333	169455.6667
NA	2'-Deoxycytidine-5'-diphosphate sodium salt	4.38	388.29999	15240	14337.66667
HMDB0003537	Epigallocatechin gallate	8.885	459.10001	88369	86617
NA	2-O-sinapoylmalate	3.53	339.60001	204755.3333	164224.6667
HMDB0000058	3',5'-Cyclic AMP	13.179	659.09998	344997	534977.3333
HMDB0000582	3,5-Diiodothyronine	3.192	525.90002	258356.3333	247948.6667
NA	3,5-Diiodo-tyrosine	3.271	434	79146	123075.3333
NA	3-Man2GlcNAc	12.164	695	605219	347769.3333
HMDB0000725	4-Hydroxy-L-proline	11.119	261.10001	38912.33333	25285.66667
HMDB0003355	5-Aminopentanoate	3.593	118	5655402.667	5722936
NA	5-beta-Androstane-3-alpha,17-beta-diol	5.764	257.29999	82510	77923
HMDB0001983	5'-Deoxyadenosine	4.081	252	50972	47411.66667
HMDB0000189	5'-ITP	4.93	509.29999	55292	52574
HMDB0002004	5-Methoxy-N,N-dimethyltryptamine	4.883	219	350927.3333	331986.3333
HMDB0002894	5-Methylcytosine	2.76	126	7710055	2592553.333
NA	6-Deoxy-alpha-L-galactose phosphate	12.487	243.2	169901	175675.6667
HMDB0002658	6-Hydroxynicotinate	4.049	140	4596087	11834019.67
HMDB0000391	7-Ketodeoxycholic acid	6.739	406.29999	146178.3333	156742.6667
HMDB0000897	7-Methylguanine	3.609	166	265903.3333	162024.6667
HMDB0001859	Acetaminophen	2.901	152.10001	117035	118689.6667
HMDB0001206	Acetyl-CoA	11.394	808.59998	387508	714915

HMDB0000034	Vitamin B4	4.82	136.10001	82495	131514.3333
HMDB0000050	Sandesin	3.224	268.29999	45892	31039.66667
HMDB0000538	Adenosine 5'-triphosphate	3.939	252.5	48550	53490
HMDB0000536	Adenylosuccinate	2.768	230.5	242732.3333	77671
HMDB0006557	Adenosine 5'-diphospho-glucose	3.051	590.09998	1914877	1871425
HMDB0000505	Allocholic Acid	5.268	409.29999	34871	100052
NA	Allocryptopine	4.765	370	82805	80584
HMDB0014622	Amikacin	6.684	586	592421	617174.3333
HMDB0000510	Aminoadipic acid	3.601	162	9027538	18970927.67
HMDB0004350	Anabasine	8.374	163	352531.3333	335157.6667
HMDB0000194	beta-Alanyl-3-methyl-L-histidine	3.342	241.3	17392	22028.33333
HMDB0000052	Argininosuccinate	4.435	291	164608.3333	95401
HMDB0000706	Aspartylphenylalanine	5.331	281.10001	40370	51828
NA	Baic-7-GlcA	2.76	445.39999	40459	45463
HMDB0003409	Berberine	0.794	336	25304	39237
HMDB0000056	beta-Alanine	3.609	90	159889.3333	113707.6667
HMDB0000852	beta-Sitosterol	6.314	397.60001	95861.66667	74709
HMDB0006335	rac-B-tocopherol	2.886	417.60001	48239	45809.66667
HMDB0001008	Biliverdin	5.952	583.29999	64231	258567.6667
HMDB0000030	Biotin	6.998	245.10001	24379	24231.66667
HMDB0011181	Brassicasterol	3.751	381.39999	403771	281946.6667
HMDB0249406	Brucine	6.165	395.20001	29523	28816.66667
NA	C10-homoserine lactone	3.271	256.20001	119083	115228.6667
HMDB0002869	Campesterol	4.317	383.39999	94219.66667	66251
HMDB0002706	Canavanine	2.878	177	107549	110953.6667
HMDB0000033	Carnosine	4.458	225.10001	337111	499444.3333
HMDB0004949	N-Palmitoyl-D-sphingosine	5.182	560.5	309901	325703.3333
HMDB0000518	Chenodeoxycholic acid	5.331	393.29999	41085	37040.66667
HMDB0000918	Cholesteryl oleate	14.838	673.59998	356011	368540.3333
HMDB0000097	Choline	1.95	104.2	550580.6667	331951.3333
HMDB0000094	Citric acid	8.17	459.20001	64740	65133
HMDB0001046	Cotinine	4.018	177.10001	123447	232717.3333
HMDB0000562	Creatinine	3.578	114	3675855	2445132.333
HMDB0002009	Crotonoyl-CoA	7.407	836.59998	446212	420490.3333
HMDB0000058	3',5'-Cyclic AMP	13.651	657.09998	376061.3333	386173.3333
NA	Cyclopamine	2.681	412.29999	68343	155919.3333
NA	Cysteine Sulfate Thioester	11.929	200.10001	23439	23703.66667
HMDB0015122	Cytosine arabinoside	8.327	244	66240.66667	35085.66667
HMDB0000089	Cytidine	2.823	244.10001	55479.66667	37129.66667
HMDB0000631	Deoxycholic acid glycine conjugate	3.743	449.29999	619286.3333	596088.3333
HMDB0000014	2'-Deoxycytidine	4.679	228	70580	65933
NA	Deoxypumiloside	10.977	497.20001	124869.3333	116969.6667
HMDB0003581	Desthiobiotin	8.272	215	131074.3333	72987
HMDB0240498	Dihydroresveratrol	14.075	231.10001	27741	26211.66667
HMDB0000269	Dihydrosphingosine	4.914	302	881691	979463.6667
HMDB0000092	N,N-Dimethylglycine	3.955	104	97133822.67	78318722

HMDB0000624	leucic acid	4.222	131.10001	48240	44503.66667
HMDB0001274	Palmitoylcarnitine	3.428	400.39999	194727.3333	180568.6667
HMDB0003153	1-tridecanoyl-2-hydroxy-sn-glycero-3-phosphocholine	3.216	454.29999	87589	123251.3333
HMDB0030554	beta-Tocotrienol	3.09	432.29999	90123	84915
HMDB0000878	Ergosterol	3.688	379.39999	43358	77355
HMDB0001926	Ethinyl estradiol	5.921	279.39999	315964.3333	327317.3333
HMDB0000622	Ethylmalonate	3.837	131.60001	366899	709201
HMDB0014887	Etodolac	5.363	288	128514.6667	36930.66667
HMDB0001248	FAD	7.832	439.10001	80078	75153
NA	Farnesyl acetate	3.232	264.29999	116498	124880.6667
NA	FucGlcNAcGA	12.652	559	428454.3333	448533.3333
NA	FucGlcNAcThrNAc	5.213	660	996131	994477
HMDB0000648	Psychosine	3.2	462	80023	74390
NA	GalNAcGlcNAcGA	4.27	616	1993703.667	1946499.333
HMDB0034367	gamma-Glutamylmethionine	10.293	279.10001	29147	28792.66667
HMDB0000594	gamma-Glutamylphenylalanine	9.286	295.10001	19767	16856.66667
HMDB0029159	gamma-Glutamylthreonine	4.726	249.10001	16929	21440.66667
HMDB0011172	gamma-Glutamylvaline	4.01	247.2	38631	39106
HMDB0035930	3,5-Dibromo-L-tyrosine	8.838	337.89999	96352	246722.3333
HMDB0012958	(R)-gamma-Tocotrienol	3.633	411.60001	554543	368012.3333
HMDB0029533	Glabrone	5.787	335	86081.66667	68108
NA	GlcNAcThrNAc	5.292	514	1445476.333	1419123
HMDB0001401	Glucose 6-phosphate	14.468	261	35634	65308
HMDB0028818	Glu-Glu	5.693	277	63288	89413
HMDB0000086	sn-Glycero-3-phosphocholine	5.268	258.10001	641862	4218900.667
HMDB0000138	Glycocholate	3.405	466	93088	181277.3333
HMDB0028854	Glycylvaline	7.73	175.10001	365947.3333	360103.3333
HMDB0011628	Glycyrrhetinate	11.048	471	163227	234578.3333
HMDB0013222	b-Guanidinopropionate	4.623	132	1136515.667	745129.3333
HMDB0000133	Guanosine	9.483	285.10001	28969	17199.66667
HMDB0000870	Histamine	13.053	112	112584	106243.6667
HMDB0000745	Homocarnosine	3.9	241	340040.3333	215519.6667
HMDB0000732	3-Hydroxykynurenine	3.947	225	213893.3333	179518.6667
NA	Hypaconitine	12.282	616.29999	596894	684990
HMDB0000157	Hypoxanthine	2.862	137.10001	326514.3333	193479.6667
NA	Indole-3-acetyl-L-tryptophan	4.034	362	4761325	8273316.667
HMDB0000197	Indoleacetic acid	14.044	176.10001	77841.66667	48994.66667
HMDB0000671	Indolelactic acid	1.604	206.10001	26702	29163.66667
HMDB0000189	dITP	5.772	493.29999	158533.3333	100220.6667
HMDB0000717	Isolithocholic acid	5.638	377.29999	60249.66667	35299.66667
NA	isopentenyl-Adenine-9-glucoside	10.348	372	69644.66667	49938.66667
HMDB0012240	Isopentenyladenine-9-N-glucoside	0.873	366	29594	354488
HMDB0037362	Isoquercetin	8.555	463	208360.3333	194675.6667
HMDB0002092	2-Propene-1,2-dicarboxylic acid	1.517	130.60001	164212	224420.3333
HMDB0012245	6-Furfurylaminopurine	3.365	216	120249.3333	89736

HMDB0000684	Kynurenine	13.077	209.10001	47055	25529
HMDB0000201	Acetylcarnitine	10.301	204.2	17229	16432.66667
HMDB0004041	allo-Threonine	3.617	120	2642372.333	2599415.333
HMDB0000452	2-Aminobutyric acid	5.654	104.1	1718077	2333053.667
HMDB0000191	L-Aspartic acid	2.839	134.10001	311535.3333	296062.3333
NA	L-beta-homoleucine-HCl	3.751	146.2	96906	192747.3333
HMDB0000062	Carnitine	5.952	161.8	210985.3333	127868.6667
HMDB0000099	Cystathionine	3.766	223	189267	1004416
	Leu-Leu-Tyr	6.786	408	359546	2041393.667
HMDB0254042	Leupeptin	7.549	427	107889	105118.6667
HMDB0000265	Liothyronine	3.184	651.79999	391095.6667	379412.3333
HMDB0000722	Lithocholyltaurine	3.413	466.29999	173515.3333	188090.6667
HMDB0000667	L-Thyronine	4.002	274.10001	6379158	8806348.667
HMDB0000182	L-Lysine	3.751	146.10001	115311	182271.3333
HMDB0002815	1-Oleoylglycerophosphocholine	13.541	522.40002	201477.3333	150934.6667
HMDB0000156	Malic acid	2.815	233.10001	25468	25645.66667
HMDB0001175	malonyl-CoA	11.661	854.40002	416822.6667	392413.3333
NA	Man3GlcNAc	12.078	857.29999	425480.3333	428706.3333
HMDB0001713	m-Coumaric acid	8.366	163.10001	202661.3333	118570.6667
HMDB0001389	Melatonin	10.112	233	63289	59564
NA	Mesaconitine	11.488	632.29999	665926.3333	652728.3333
HMDB0014567	Methocarbamol	3.586	242.10001	8881828	8799614
HMDB0015474	Methotrimeprazine	10.773	329	17343	15301.66667
HMDB0001186	N1-Acetylspermine	3.798	245	387169	75407
NA	N6-(gamma,gamma-Dimethylallyl)adenosine	3.2	336.39999	33143	34311.66667
HMDB0032055	N-Acetylhistidine	6.361	198	1392085.667	88933
NA	N-Acetylneuraminate	5.441	310	74298.66667	43149.66667
HMDB0000902	NAD+	13.265	662.09998	611119.3333	639400.3333
NA	Nari-7-Glc	2.854	433.39999	50099	49471.66667
HMDB0033740	Narirutin	13.281	581	381863	396724.3333
NA	N-Benzyl dimethyl stearyl ammonium	3.381	388.39999	78165.66667	45405.66667
NA	Neoxaline	4.073	436.20001	1762069	1789295
NA	N-Fructosyl isoleucylglutamate	3.79	423.20001	188431	253976
HMDB0031874	N-gamma-Glutamyl-S-allylcysteine	14.28	291.10001	25104	23758.66667
HMDB0000833	N-Glycolylneuraminate	6.637	326	273156	2543907.667
HMDB0004949	N-Palmitoyl-D-sphingosine	9.648	520.5	407693	215560.6667
NA	N-Stearoyl tyrosine	3.774	447.29999	917609	1203794.667
HMDB0002088	Oleoyl Ethanolamide	3.263	326.29999	28713	24538.66667
NA	Oleyl sarcosine	3.153	354.29999	50457	48562.66667
NA	Oxaline	3.216	448.20001	195624	184306.6667
HMDB0003337	Oxidized glutathione	6.196	613	689656.3333	459371.3333
HMDB0030172	Oxyacanthine	4.568	609.29999	384825	708968
HMDB0015396	9-OH-Risperidone	2.713	413.20001	211394	201817.6667
NA	Palmatine	2.878	352	76620.66667	51299
HMDB0000222	Palmitoylcarnitine	5.882	399.29999	70996	69377
HMDB0010570	1,2-dipalmitoylphosphatidylglycerol	4.057	723	363222	552342.3333

HMDB0036634	Phlorhizin	5.504	437.39999	77091	77252
HMDB0001565	Phosphorylcholine	3.428	185	904999	683813.3333
HMDB0004610	Phytosphingosine	3.271	318.29999	21997	18478.66667
HMDB0000070	DL-Pipecolic acid	4.6	130.10001	601922.3333	614925.3333
HMDB0001830	Progesterone	3.413	315.39999	30908	26746.66667
HMDB0000162	Proline	3.719	116.1	55553286	35616411.67
HMDB0034580	Pseudopelletierine	9.601	154	92776	89820
HMDB0240265	Puerarin	4.985	415.10001	52583	53780
HMDB0001366	Purine	2.791	121	992144.6667	599272.3333
HMDB0030775	Quercetin 3-beta-D-galactopyranoside	8.492	463.39999	64066	60335.66667
NA	Riboflavin-5'-monophosphate sodium salt hydrate	4.576	457.39999	166386	171221.6667
HMDB0000884	5-Methyluridine	11.913	259.10001	32653	29931.66667
HMDB0003249	Rutin	11.52	609	630259.6667	660138.3333
HMDB0000939	S-Adenosyl-L-homocysteine	14.083	383.10001	29586.66667	25477.66667
HMDB0001185	S-Adenosyl-L-methionine	13.022	298.10001	45593	25393.66667
HMDB0029367	Sanguinarine	7.156	333	93710.66667	44104.66667
HMDB0001066	S-Lactoylglutathione	4.867	380	82952	78087
HMDB0000269	Sphinganine	12.833	302.8	27603	18177.66667
HMDB0000256	Squalene	6.731	411.39999	30726	29531.66667
HMDB0000937	Stigmasterol	6.361	395.5	42264	55243
HMDB0001259	4-Oxobutanoic acid	4.993	102.1	911697	874541.3333
HMDB0031554	Sucralose	4.482	419	68556	137066.3333
HMDB0015619	Sulfathiazole	7.682	256	46220	45239.66667
HMDB0000036	Taurocholate	3.208	516.29999	240163	233844.6667
HMDB0000896	Taurodeoxycholate	12.385	500.29999	84062	88531
HMDB0005066	Myristoyl-L-carnitine	3.216	394.29999	121917	125071.6667
HMDB0002666	Thiamine monophosphate	4.993	346	808837	268072.6667
HMDB0001372	Cocarboxylase	6.204	426	117586	108093.6667
HMDB0000273	Thymidine	2.925	243	515200	529334.3333
HMDB0000248	L-Thyroxine	12.212	777.70001	955954	572588.3333
HMDB0014643	Tolmetin	5.276	258	2294736	9629877.667
HMDB0001124	Trehalose 6-phosphate	14.744	423	99452	97610
NA	Tropine	2.807	142	4501278	4441793
HMDB0006709	Coenzyme Q2	4.317	319.20001	133603	137010.6667
HMDB0000302	UDP-galactose	14.697	565.40002	519725	1747935.667
NA	UDP-Galactose disodium salt	3.759	567.40002	3265605.667	1887345.333
HMDB0000294	Urea	6.338	121.2	78623	122895.3333
HMDB0000289	Uric acid	3.892	169.10001	230358.3333	217198.6667
HMDB0000296	Uridine	5.787	268.10001	17740	15318.66667
HMDB0000935	Uridine-diphosphate-glucuronic acid	3.743	579.40002	2255584	2225391.333
HMDB0000302	UDP-galactose	7.832	565.40002	192358	257745
HMDB0000304	Uridine diphospho-2-acetamido-2-deoxy-D-galactose	12.644	606.5	638716.3333	617580.3333
NA	Uridine-5'-diphosphate sodium salt	3.9	405.29999	85399	197836.6667
HMDB0259814	Vindoline	8.587	457	350441	148483.6667
NA	Z-Gly-Pro	9.632	307	12585	40752

HMDB0000214	Ornithine	5.677	133.10001	371647.3333	385318.3333
HMDB0000148	Glutamic Acid	2.846	148	438766.3333	255251.6667
HMDB0000641	Glutamine	3.617	147.10001	3567949.667	3630360.333
HMDB0000177	Histidine	5.693	156	193128	407562.3333
HMDB0000517	Arginine	3.586	175.10001	696587	999392
HMDB0000168	Asparagine	10.867	133	579250	913502
HMDB0000574	Cysteine	13.981	122.3	85653	82360
HMDB0000687	Leucine	0.464	132.10001	409808	394211.3333
HMDB0000167	Threonine	3.908	120	446347	1072011.667
HMDB0000929	Tryptophan	3.625	205.10001	390565.3333	323276.3333
HMDB0000696	Methionine	7.203	150	107905.3333	164351.6667
HMDB0000232	Quinolinic Acid	0.637	167.89999	118464	463568
NA	NAE 4:0	3.593	132.10001	7747364	962423
NA	NAE 10:0	3.145	216.2	126790	479182
NA	NAGly 10:0	3.932	247.2	39663.33333	1695251.667
NA	NAGly 12:0	3.263	258.20001	51112.33333	990317.6667
NA	CAR 7:0	3.208	274.20001	25928.66667	1284227.667
NA	NAGly 15:3	3.035	294.20001	49493.66667	739464
NA	NAE 17:0	3.279	314.29999	794067	91556
NA	SPB 18:0;O3	3.279	318.29999	23549.66667	436785.3333
NA	NAGly 16:4;O	3.326	322.20001	27060.66667	666480
NA	Cer 20:0;O2	3.224	326.29999	33104.33333	13430531.67
NA	DG O-15:0	3.2	334.29999	47531.33333	739070
NA	Cer 21:1;O2	3.507	338.29999	41918.33333	1654970.667
NA	Cer 20:0;O3	3.169	342.29999	89533	682806
NA	Cer 22:2;O2	3.405	350.29999	19688.66667	395325
NA	Cer 21:1;O3	3.153	354.29999	59591.33333	420914.3333
NA	NAOrn 14:0;O	3.766	359.29999	79914.33333	718137
NA	Cer 23:3;O2	3.098	362.29999	925329.6667	768131.6667
NA	Cer 22:2;O3	3.515	366.29999	69735	654721.6667
NA	NAGly 19:3	3.625	367.29999	16410.66667	338187.6667
NA	LPC 7:0	3.271	370.20001	114745	199362
NA	CAR 14:1	3.279	370.29999	144648	308731
NA	NAE 22:5	3.279	374.29999	57062.33333	523686.6667
NA	NAE 22:5	3.491	374.29999	212909	113710.3333
NA	Cer 20:0;O4	3.279	376.29999	179165	147177.6667
NA	Cer 23:3;O3	3.491	378.29999	49389.33333	396487.6667
NA	NAGly 20:4	3.829	379.29999	53758.33333	181254
NA	LPC 8:1	3.255	382.20001	323932	150925
NA	CAR 15:2	3.279	382.29999	660719.3333	140636.3333
NA	SPB 24:0;O2	2.894	386.39999	40540.33333	247968.3333
NA	Cer 21:1;O4	3.601	388.29999	71170.33333	183338
NA	NAGly 18:1;O2	2.854	389.29999	49595.33333	344635.6667
NA	NAGly 22:4	3.216	390.29999	190092	185024.3333
NA	NAGly 21:5	3.538	391.29999	45825.33333	320665.6667
NA	CAR 16:3	3.208	394.29999	104775	399731.6667

NA	NAE 23:0	2.839	398.39999	97514.66667	457296
NA	Cer 22:2;O4	3.184	400.29999	238366.6667	597073
NA	NAOrn 16:1;O2	2.878	401.29999	26860.66667	675023
NA	Cer 23:3;O2	3.184	402.29999	244607.6667	837915.6667
NA	NAGly 22:6	3.751	403.29999	559204.6667	330741
NA	TG 18:0	3.192	404.29999	96200	586250
NA	ST 27:0;O	6.754	406.39999	397537	423242.6667
NA	Cer 26:0;O2	3.169	410.39999	393055.3333	546733.6667
NA	NAGly 24:6	4.199	414.29999	295406	351322
NA	ST 28:1;O	7.258	418.39999	57526.33333	1193798.667
NA	Cer 27:1;O2	9.735	422.39999	35675	479279.3333
NA	ST 29:2;O	3.098	430.39999	3860577	546770
NA	BMP 10:0	3.271	432.20001	310919	742777.3333
NA	Cer 28:2;O2|Cer 12:0;O2/16:2	2.956	434.39999	62902	459702.6667
NA	Cer 29:3;O2	3.019	446.39999	271188	459032.6667
NA	NAGlySer 16:1;O2	3.224	448.29999	212981	363421.6667
NA	CAR 20:0	6.825	456.39999	170544.6667	213638.3333
NA	SE 28:2/2:0	3.192	458.39999	238039.6667	120637.3333
NA	Cer 26:0;O4	5.26	460.39999	100511	137932
NA	TG 22:1	3.68	463.29999	675711	580819.6667
NA	PS 13:1	3.177	468.20001	277989	130864.6667
NA	CAR 21:1	7.454	468.39999	121206.3333	101264
NA	NAOrn 22:1;O	3.633	469.39999	785453.3333	127112.3333
NA	Cer 27:1;O4	8.72	472.39999	281601.6667	3961124.667
NA	TG 23:2	5.394	475.29999	66393.33333	209686.3333
NA	CAR 22:2	3.491	480.39999	422028.6667	4906104
NA	Cer 32:6;O2	3.161	482.39999	102205.6667	271933
NA	Cer 28:2;O4	2.909	484.39999	134739	237925
NA	PS 13:0	3.027	492.20001	391358.3333	19064.66667
NA	Cer 30:3;O4	1.171	492.39999	125938.6667	26889.66667
NA	Cer 33:7;O2	5.551	494.39999	257206.3333	60189.66667
NA	Cer 29:3;O4	3.515	496.39999	3617227.667	180418
NA	TG 25:4	3.892	499.29999	1020074.333	41245.66667
NA	PS 14:1	3.861	504.20001	733382	50913.66667
NA	CAR 24:4	3.004	504.39999	240405.6667	62948.66667
NA	VAE 13:0	3.743	505.39999	176178.3333	23332
NA	Cer 34:8;O2	3.656	506.39999	506978.6667	68261
NA	DG 26:0	14.524	507.39999	97140.33333	219427.3333
NA	PC 16:1	3.224	508.29999	351218	207033.3333
NA	Cer 30:4;O4	6.361	508.39999	228809.6667	699200.6667
NA	SL 26:0;O	5.001	509.39999	72332.33333	58421.66667
NA	Cer 32:5;O4	12.605	516.40002	159980.6667	361348.6667
NA	DG O-28:0	4.317	516.5	938546.6667	59633.66667
NA	VAE 14:1	3.971	517.40002	7956977.667	57339
NA	DG 27:1	3.703	519.40002	737061.3333	96920.66667
NA	PE 20:2	3.46	520.29999	685673.3333	48737.66667

NA	Cer 31:5;O4	9.538	520.40002	346674	125877
NA	SL 27:1;O	9.365	521.40002	241352.6667	253501.3333
NA	Cer 32:0;O3	4.27	528.5	429483	2686977.667
NA	LPC 18:1	14.358	529.40002	122731.3333	85383.66667
NA	DG 28:2	4.002	531.40002	754937	195162
NA	Cer 32:6;O4	12.683	532.40002	159895.3333	337305
NA	SL 28:2;O	5.473	533.40002	255516.6667	215170.3333
NA	TG 25:0;O2	10.427	534.40002	471797.6667	129075.3333
NA	TG 27:0	3.625	535.40002	1607263.333	84147.66667
NA	SL 28:0;O2	5.441	536.40002	13565197	260355
NA	SM 22:0;O2	4.482	537.40002	375262.3333	193578
NA	NAOrn 28:1	14.956	537.5	113429.3333	2853431.667
NA	Cer 34:7;O4	3.027	540.40002	487081.6667	332893
NA	Cer 34:7;O4	3.53	540.40002	3272788	445628
NA	CAR 26:0	3.043	540.5	474945.6667	5655355.667
NA	VAE 16:3	6.951	541.40002	246541.6667	445486
NA	VAE 16:3	13.493	541.40002	253842.6667	2112718.667
NA	TG 27:2;O	3.751	542.40002	2461658.667	462077.6667
NA	DG 29:3	12.825	543.40002	374144.3333	432129.3333
NA	PC 17:1	4.616	544.29999	6017539	339766
NA	Cer 33:7;O4	4.616	544.40002	8455148.667	836463.6667
NA	SL 29:3;O	13.934	545.40002	308671	402639
NA	TG 26:1;O2	3.656	546.40002	8762919.667	598758
NA	TG 28:1	8.39	547.40002	210227.3333	377541
NA	SL 29:1;O2	1.085	548.40002	362137.3333	560692.6667
NA	PC O-20:0	5.402	552.40002	2559079	263089.3333
NA	CAR 27:1	4.718	552.5	174407.3333	1522469.667
NA	PI 12:0	4.152	553.20001	571289.6667	577219.6667
NA	VAE 17:4	4.183	553.40002	1779691.667	664154
NA	VAE 18:0	4.175	553.5	2724730	381064.6667
NA	TG 28:3;O	11.952	554.40002	177735.3333	656695.6667
NA	Cer 34:8;O4	3.672	556.40002	3919383	845822.6667
NA	TG 27:2;O2	3.004	558.40002	241763.6667	2022358.667
NA	TG 29:2	11.111	559.40002	200394	508066.6667
NA	SL 30:2;O2	12.589	560.40002	347665.6667	2351207.667
NA	Cer 34:1;O2	5.15	560.5	323126	405841.6667
NA	SM 24:2;O2	13.47	561.40002	295996	698052
NA	CAR 28:2	11.307	564.5	173795.3333	366089.6667
NA	PI 13:1	14.429	565.20001	303339.3333	606378.6667
NA	VAE 18:5	7.879	565.40002	203141.3333	1906361.667
NA	VAE 19:1	3.09	565.5	634407.6667	556212.3333
NA	TG 29:4;O	14.201	566.40002	282748.3333	1058032.667
NA	DG 31:5	3.774	567.40002	3671055.667	612021
NA	PE 22:3	3.499	568.29999	16208792.67	1709145.667
NA	NAGly 33:8;O2	3.499	568.40002	19920258.67	571938.3333
NA	TG 30:3	12.66	571.40002	423894.3333	997713.6667

NA	SL 31:3;O2	14.602	572.40002	274580.3333	404988.6667
NA	Cer 35:2;O2	14.728	572.5	796249	574554
NA	SM 23:0;O2	8.076	573.40002	190214.3333	504235
NA	PE O-25:2	14.327	576.40002	469475.6667	638235
NA	TG 30:5;O	3.782	578.40002	1297933.667	492731.6667
NA	DG 32:6	3.727	579.40002	2458142.333	691076
NA	PC 20:4	4.183	580.29999	1745291.667	548109
NA	NAGly 34:9;O2	8.791	580.40002	524960	609071.6667
NA	SL 32:6;O	4.844	581.40002	292750	532389.3333
NA	TG 31:4	2.878	583.40002	562256.6667	983374
NA	SL 32:4;O2	7.368	584.40002	464729.6667	358247.6667
NA	SM 25:3;O3	7.863	589.40002	5245074	613277
NA	VAE 21:3	7.871	589.5	13137424.67	244759.3333
NA	PE 24:5	2.988	592.29999	424058.3333	636849
NA	NAGly 35:10;O2	4.655	592.40002	492115.6667	632364
NA	Cer 39:0;O2	7.038	592.59998	430894.3333	807381
NA	SL 33:7;O	12.393	593.40002	559013.6667	1148774.667
NA	TG 32:5	7.266	595.40002	277838	499549
NA	SL 33:5;O2	5.174	596.40002	402997	395513.6667
NA	Cer 37:4;O2	13.674	596.5	358472.3333	521348.3333
NA	Cer 38:0;O2	4.553	596.59998	376481.3333	433643.6667
NA	SM 25:2;O2	4.34	597.40002	2367812	302599.3333
NA	LPC 22:1	14.814	600.40002	574523.6667	392208.6667
NA	Cer 38:5;O4	14.807	600.5	717899	308121.3333
NA	VAE 22:4	3.169	601.5	220532	326507
NA	TG 31:0;O	14.067	602.5	432352	532968.6667
NA	DG 34:8	11.787	603.40002	742547.3333	298370.3333
NA	SL 34:0;O	13.234	604.5	3162111.667	665091.6667
NA	TG 33:6	4.671	607.40002	384549.3333	194267
NA	TG 33:6	11.921	607.40002	457366.6667	288395
NA	SL 34:6;O2	14.138	608.40002	519511.6667	969395.6667
NA	SM 26:3;O2	12.463	609.40002	357411	211947.3333
NA	TG 33:2	2.988	610.5	392144	766064.6667
NA	PE O-28:5	13.863	612.40002	519804.6667	166930.3333
NA	Cer 39:6;O4	4.623	612.5	579314.6667	244751
NA	SM 27:5;O3	8.736	613.40002	320671.3333	307593
NA	BMP 23:0	4.734	614.40002	473600.6667	677796
NA	HexCer 29:0;O2	14.775	614.5	534106.6667	232629.3333
NA	DG 35:9	4.16	615.40002	587127.6667	218034
NA	DG 34:2	9.082	615.5	403824.3333	149154
NA	PC 24:3	11.488	616.40002	731964.3333	216303.3333
NA	SL 35:1;O	12.495	616.5	664312.6667	1442034
NA	Cer 41:2;O2	3.735	616.59998	1528005.667	195461
NA	TG 34:7	7.022	619.40002	411332	209485.3333
NA	Cer 40:1;O3	2.964	620.59998	369971.3333	287444.3333
NA	SM 27:4;O2	5.544	621.40002	646336	236328

NA	SM 28:0;O2	5.371	621.5	627797.3333	182040.3333
NA	SE 24:1	14.13	622.5	345237	157772.3333
NA	SL 33:0;O2	3.129	623.5	997692.3333	1648031.667
NA	LPC 24:3	4.215	624.40002	983927.6667	2348383.667
NA	Cer 40:7;O4	10.812	624.5	422385	1466953.667
NA	VAE 24:6	3.137	625.5	723654	1924416.667
NA	BMP 24:1	12.896	626.40002	652865.3333	2242003
NA	HexCer 30:1;O2	13.651	626.5	401608	347023.6667
NA	DG 36:10	7.895	627.40002	458560.6667	806522
NA	DG 35:3	4.443	627.5	609952.6667	734546
NA	PC 25:4	11.26	628.40002	685491.3333	678005
NA	TG 35:8	14.736	631.40002	1126655.333	524158.3333
NA	SL 36:8;O2	3.515	632.40002	6253648	1353888.667
NA	Cer 41:2;O3	11.221	632.59998	811585	299161.3333
NA	SM 28:5;O2	3.098	633.40002	1312333.333	402451
NA	SM 29:1;O2	3.09	633.5	2371156.333	536708.3333
NA	SE 24:1;O4	11.41	634.5	581876.6667	670886
NA	SL 34:1;O2	4.836	635.5	556678.6667	349759
NA	PE O-30:7	4.293	636.40002	443957.3333	646988.6667
NA	Cer 41:8;O4	4.427	636.5	1033677.667	585453
NA	BMP 25:2	2.98	638.40002	605662.6667	1386891
NA	HexCer 31:2;O2	4.411	638.5	793868	456749
NA	DG 36:4	4.199	639.5	937508	516143.6667
NA	SE 28:2	2.996	640.59998	672300	704739.6667
NA	SL 37:9;O2	14.586	644.40002	586068.6667	289046
NA	SM 29:6;O2	12.715	645.40002	547895.6667	791445.6667
NA	SM 30:2;O2	11.85	645.5	591129.6667	322130.6667
NA	SE 24:1;O4	7.533	646.5	1619164.667	357647.6667
NA	SL 35:2;O2	4.411	647.5	441131	393510
NA	spb	14.602	648.40002	431067	367590.3333
NA	Cer 42:9;O4	7.84	648.5	820438	427926.6667
NA	PI 19:1	4.254	649.29999	587403.6667	723952.6667
NA	BMP 26:3	3.161	650.40002	335517	825019.6667
NA	DG 37:5	3.184	651.5	364416	1739577.667
NA	PC 27:6	3.499	652.40002	9353067.333	3535102
NA	SL 38:4;O	14.39	652.5	412836.3333	135549.3333
NA	SE 28:2	12.526	652.59998	674857.3333	266438
NA	Cer 40:1;O4	4.364	654.59998	1042622.333	174619
NA	NAGly 37:1;O2	3.184	655.59998	436459.3333	540915.6667
NA	SHexCer 25:0;O2	3.043	656.40002	1135809.333	144251.3333
NA	SM 31:3;O2	11.158	657.5	462114.6667	159254.3333
NA	SE 24:1;O4	12.369	658.5	515959.6667	191928
NA	SL 36:3;O2	12.369	659.5	431297.3333	188097.3333
NA	SL 36:3;O2	3.161	659.5	5746608	52573.66667
NA	PC O-28:2	13.077	660.5	526251.6667	866037
NA	PI 20:2	11.221	661.29999	413437	1393610.667

NA	BMP 27:4	4.812	662.40002	857742	970649
NA	ASG 28:2;O	4.27	662.5	509326.6667	297920
NA	DG 38:6	14.563	663.5	413627.3333	883788
NA	PE 31:7	3.711	664.40002	512719.6667	216487.3333
NA	PE 31:7	4.364	664.40002	3587064.333	885719.6667
NA	HexCer 29:0;O4	3.043	664.5	815426	458597.3333
NA	SE 28:2	14.406	664.59998	620869.3333	154556
NA	NAGly 39:9;O2	13.997	667.5	404010.3333	2010348.667
NA	SHexCer 26:1;O2	3.578	668.40002	2610643	541908.3333
NA	Cer 44:5;O3	12.723	668.59998	576818.6667	361921.6667
NA	SM 32:4;O2	12.652	669.5	451991.6667	478772.3333
NA	SE 24:1;O4	4.427	670.5	888875.6667	369993
NA	TG 37:0	7.596	670.59998	336314	232045.3333
NA	SL 37:4;O2	3.161	671.5	868719	223136.3333
NA	PC O-29:3	8.791	672.5	535916.6667	222097.3333
NA	VAE 26:7	4.474	673.5	1190596.667	171211
NA	PS 26:0	3.633	674.40002	12593080.67	403554.6667
NA	HexCer 34:5;O2	8.65	674.5	600053.6667	859927
NA	DG 39:7	11.936	675.5	405577	413023.6667
NA	PE 32:8	5.371	676.40002	871224.3333	201673
NA	HexCer 30:1;O4	12.33	676.5	715347	305556.3333
NA	SE 28:2	12.652	676.59998	540529.6667	215040.3333
NA	Cer 42:3;O4	3.153	678.59998	434948.3333	168739
NA	NAGly 40:10;O2	4.34	679.5	1008667.333	808159
NA	NAGly 39:3;O2	3.059	679.59998	905268.3333	285181.3333
NA	SHexCer 27:2;O2	13.077	680.40002	424506.3333	694479
NA	Cer 45:6;O3	4.836	680.59998	447438	742989
NA	SM 33:5;O2	3.153	681.5	937531.6667	497246.6667
NA	TG 39:8	13.737	682.5	479307.6667	268807.3333
NA	TG 38:1	14.995	682.59998	462439.6667	588545
NA	SL 38:5;O2	5.158	683.5	425873	525455.6667
NA	SHexCer 26:1;O3	13.202	684.40002	407852	531076.6667
NA	PC O-30:4	8.956	684.5	461650	337626
NA	PI 22:4	7.95	685.29999	337627	685744
NA	PS 27:1	4.105	686.40002	816395.3333	637481
NA	HexCer 35:6;O2	4.616	686.5	632424.6667	422626.6667
NA	DG 40:8	3.137	687.5	708127	574947.3333
NA	PE 33:9	4.183	688.40002	1565731.667	353580
NA	HexCer 31:2;O4	2.917	688.5	608719.6667	446773
NA	SE 28:2	11.905	688.59998	510102.6667	1447430.667
NA	Cer 43:4;O4	3.68	690.59998	814233.3333	1113296.667
NA	Cer 43:4;O4	10.065	690.59998	1819484.667	586927
NA	NAGly 40:4;O2	13.132	691.59998	389249	367898
NA	SHexCer 28:3;O2	14.233	692.40002	353442	344264.6667
NA	Cer 46:7;O3	14.225	692.59998	902411	2095020
NA	SM 34:6;O2	4.537	693.5	554618.6667	306837

NA	TG 40:9	2.917	694.5	482732	108105
NA	TG 39:2	11.842	694.59998	361215.3333	192726
NA	SL 39:6;O2	3.019	695.5	1139024.333	238703
NA	SHexCer 27:2;O3	3.499	696.40002	11421606.33	545685
NA	PC O-31:5	14.956	696.5	927204.6667	675907.6667
NA	PI 23:5	4.592	697.29999	598955.6667	421472.3333
NA	PS 28:2	10.356	698.40002	288554.3333	683446.6667
NA	HexCer 36:7;O2	14.72	698.5	495691.6667	498471.3333
NA	DG O-41:0	3.098	698.70001	977155.6667	104803.6667
NA	DG 41:9	3.192	699.5	374634.3333	323599
NA	HexCer 32:3;O4	4.899	700.5	462258	559965.3333
NA	SE 28:2	3.067	700.59998	559450.6667	127078
NA	Cer 44:5;O4	9.617	702.59998	1416671.667	96507
NA	NAGly 42:12;O2	14.775	703.5	758419	65419
NA	SHexCer 29:4;O2	10.45	704.40002	735709.3333	207656.3333
NA	Cer 47:8;O3	10.317	704.59998	1017404.667	76946.66667
NA	SM 35:7;O2	11.842	705.5	686851	9160173.667
NA	SM 35:7;O2	13.069	705.5	522254.6667	45756.66667
NA	TG 41:10	3.004	706.5	662430.3333	56218
NA	SL 40:7;O2	11.976	707.5	361857	42475
NA	SL 39:0;O2	10.151	707.59998	617287.6667	29114.66667
NA	SHexCer 28:3;O3	4.71	708.40002	404920	23851.66667
NA	PC O-32:6	4.71	708.5	404632	16190.66667
NA	HexCer 37:8;O2	15.074	710.5	450225.6667	59596
NA	Cer 45:0;O3	9.349	710.70001	352257	52954.66667
NA	DG 42:10	3.184	711.5	722697	176447
NA	PI 24:2	3.633	712.40002	5289404	333190.6667
NA	HexCer 33:4;O4	4.395	712.5	589667.6667	333068.3333
NA	SE 28:1	4.238	712.70001	738155.3333	15507806.67
NA	NAOrn 40:12;O2	3.798	715.5	2910502.667	403069.6667
NA	NAGly 42:6;O2	3.798	715.59998	1002170.667	517821
NA	NAGly 42:6;O2	5.213	715.59998	3089810.667	400263
NA	Cer 48:9;O3	13.989	716.59998	520732	822466.6667
NA	TG 40:0	4.27	717.59998	882954.3333	477523.6667
NA	SL 41:0;O2	10.568	718.59998	403175.3333	779750.6667
NA	SL 41:8;O2	3.184	719.5	780021	644360
NA	SL 40:1;O2	3.184	719.59998	750995	1439158.667
NA	PC O-33:7	10.576	720.5	583281.6667	1263114.667
NA	PS 30:4	4.38	722.40002	944260.6667	2322317.667
NA	HexCer 38:9;O2	4.38	722.5	872236	625516.6667
NA	Cer 46:0;O4	11.071	722.70001	978580.6667	1479129.667
NA	DG 43:11	3.177	723.5	652252	604164
NA	PI 25:3	12.935	724.40002	437032	792000
NA	HexCer 34:5;O4	6.487	724.5	344440	378836.6667
NA	SE 28:2	13.132	724.70001	481407.6667	338122
NA	PC 30:1	14.516	726.5	464738.6667	2488902.667

NA	Cer 45:0;O4	3.129	726.70001	642046.6667	207724.3333
NA	NAGly 43:7;O2	4.27	727.59998	841858	522841
NA	SHexCer 31:6;O2	3.578	728.40002	8947945	89577.66667
NA	HexCer 36:0;O3	3.161	728.59998	345546.3333	7558
NA	SL 42:1;O2	4.561	730.59998	825201	24941.66667
NA	SHexCer 30:5;O3	4.796	732.40002	1489892.667	956338.6667
NA	PC O-34:8	11.567	732.5	679566.3333	227087.3333
NA	PS 31:5	13.297	734.40002	426919.3333	1678576.667
NA	BMP 32:3	5.559	734.5	411134	425839.6667
NA	DG 44:12	3.082	735.5	1423446.667	376006
NA	SM 35:0;O3	5.433	735.59998	629314.6667	1812100.667
NA	PI 26:4	10.631	736.40002	568028.6667	2381984.667
NA	PI 26:4	11.984	736.40002	1541824.333	814071.6667
NA	HexCer 35:6;O4	11.984	736.5	497930.6667	403079.6667
NA	SE 28:2	3.586	736.70001	1168416.667	9432291
NA	PC 31:2	12.715	738.5	859171	485252
NA	Cer 46:1;O4	3.617	738.70001	785946	666115.6667
NA	NAGly 44:8;O2	2.815	739.59998	905639	359915.6667
NA	SHexCer 31:0;O2	14.665	740.5	1131709.333	1102706.667
NA	AHexCer 37:0;O2	14.665	740.59998	1034209.667	561613.3333
NA	TG 42:2	0.621	741.59998	1242097.333	418301.6667
NA	Cer 47:1;O2	12.031	742.70001	836489.3333	343675.6667
NA	SL 42:3;O2	3.145	743.59998	551644.6667	347832
NA	SHexCer 31:6;O3	3.051	744.40002	740563.3333	388960
NA	PC O-35:9	6.094	744.5	487355	2421423
NA	BMP 33:4	3.043	746.5	533854.6667	575171
NA	Cer 48:2;O4	4.474	746.70001	500624.6667	356498.6667
NA	SM 36:1;O3	3.051	747.59998	414857	781525
NA	PI 27:5	3.759	748.40002	3900087.667	7584108.667
NA	HexCer 36:7;O4	5.717	748.5	352127	660872
NA	PC 32:3	11.339	750.5	437939	170355.3333
NA	Cer 47:2;O4	4.112	750.70001	472424.6667	4270363.667
NA	SHexCer 33:8;O2	12.212	752.40002	636941.6667	556953
NA	SHexCer 32:1;O2	13.25	752.5	337261.3333	769881
NA	AHexCer 38:1;O2	12.393	752.59998	420880.3333	283841.3333
NA	AHexCer 38:1;O2	13.053	752.59998	434664.3333	942615.6667
NA	SL 44:3;O2	6.739	754.59998	692985	545889.3333
NA	SL 43:4;O2	11.134	755.59998	956026.6667	478150.6667
NA	PC O-36:1	9.805	756.5	607678.6667	1277196.667
NA	PI 28:6	12.927	760.40002	1225079.667	385596.6667
NA	SE 28:2	3.004	760.70001	536242.6667	696144.6667
NA	PC 34:0	12.825	762.59998	1547618.667	466933
NA	Cer 48:3;O4	14.657	762.70001	1064235.667	702406
NA	SHexCer 33:2;O2	14.06	764.5	634860.6667	663669
NA	AHexCer 39:2;O2	3.114	764.59998	1779202.667	210308.3333
NA	SL 45:4;O2	4.553	766.59998	826144	376978.3333

NA	SL 44:5;O2	2.854	767.59998	2450033	364770.6667
NA	SHexCer 33:8;O3	9.129	768.40002	532476	217439.3333
NA	PE O-40:11	4.128	768.5	524400	1153893.667
NA	PS 34:8	13.281	770.40002	464295.6667	11843393.67
NA	Cer 50:4;O4	13.824	770.70001	371876.3333	364097.6667
NA	SM 38:3;O3	5.237	771.59998	547628.6667	588688
NA	Cer 53:8;O2	11.166	772.70001	560444.6667	3925480
NA	Hex2Cer 28:3;O2	4.23	774.5	467956.6667	933424
NA	PC 35:1	4.639	774.59998	349291.3333	720039.6667
NA	Cer 49:4;O4	4.631	774.70001	362984.3333	433473.3333
NA	AHexCer 40:3;O2	4.789	776.59998	481658.6667	437199
NA	Cer 50:4;O2	12.487	778.70001	460662.6667	488261
NA	Cer 51:0;O2	13.297	778.79999	563673.6667	522665.3333
NA	AHexCer 39:2;O3	11.936	780.59998	1098630.667	396027.6667
NA	PS 35:9	3.924	782.40002	1789638.667	224324.3333
NA	LPC 35:1	5.213	782.59998	785182	164999
NA	DG O-47:0	13.147	782.79999	1042239.667	118775.3333
NA	SM 39:4;O3	3.279	783.59998	666125	2782091.667
NA	PI 30:8	3.153	784.40002	489689.6667	1156977.667
NA	MGDG 36:10	3.145	784.5	472142.6667	682362
NA	AHexCer 38:2;O3	14.209	784.59998	1324427.667	279488.3333
NA	Hex2Cer 29:4;O2	14.099	786.5	484097.6667	315123
NA	PC 36:2	10.765	786.59998	566963.6667	520904.6667
NA	SL 47:0;O	13.069	786.70001	429497.3333	7237239.667
NA	SL 46:1;O	3.177	787.70001	499172.6667	742726
NA	SHexCer 35:4;O2	10.678	788.5	278036.3333	623942.3333
NA	AHexCer 41:4;O2	10.67	788.59998	267656.3333	593897.6667
NA	TG 46:6	3.263	789.59998	538552.6667	2923880.667
NA	Cer 52:0;O3	12.786	790.79999	378114	1378170.667
NA	SL 46:7;O2	14.012	791.59998	650781	497504
NA	SHexCer 34:3;O3	4.364	792.5	759538	573950
NA	AHexCer 40:3;O3	4.914	792.59998	368943.3333	347436.6667
NA	PS 36:10	14.846	794.40002	445098	704997
NA	Cer 52:6;O4	4.215	794.70001	686306	635151.6667
NA	SM 40:5;O3	3.295	795.59998	578238.6667	1055608.667
NA	PI 30:2	3.169	796.5	512573.6667	420755
NA	DG 47:2	3.161	797.70001	481043.6667	322914
NA	PC 36:7	10.112	798.5	786332	719375.6667
NA	PC 37:3	10.12	798.59998	355349	966421.6667
NA	SL 48:1;O	3.122	798.70001	1327419.667	407750
NA	NAOrn 46:12;O2	3.42	799.59998	621700.3333	582443
NA	SL 47:2;O	3.578	799.70001	1296733.667	577789
NA	SHexCer 36:5;O2	5.52	800.5	399953.3333	356277
NA	AHexCer 42:5;O2	5.135	800.59998	302116.3333	1403436
NA	HexCer 41:0;O2	3.059	800.70001	596631.6667	369023.6667
NA	TG 47:7	5.96	801.59998	482810.6667	340497.6667

NA	SM 41:0;O2	4.639	803.70001	891912	981939.6667
NA	SHexCer 35:4;O3	11.009	804.5	534714	512754
NA	SE 24:1	14.303	804.70001	697584	170436
NA	BMP 38:9	12.4	806.5	689614	608349.6667
NA	Cer 53:7;O4	14.075	806.70001	709294.3333	89361.66667
NA	Cer 52:0;O4	4.246	806.79999	561487.6667	287683
NA	SM 41:6;O3	3.161	807.59998	447809	346880
NA	PI 31:3	11.701	808.5	773376.3333	282206
NA	HexCer 43:1;O2	13.407	808.70001	371051.3333	441755.6667
NA	DG 49:10	3.011	809.59998	888478.6667	11528859.67
NA	DG 48:3	3.035	809.70001	869702	765496
NA	Hex2Cer 31:6;O2	11.779	810.5	466824.6667	543756.3333
NA	PC 38:4	10.222	810.59998	361347	810277.6667
NA	SL 48:3;O	10.474	811.70001	435766.3333	494026.3333
NA	SHexCer 37:6;O2	6.983	812.5	535604.6667	516771
NA	AHexCer 43:6;O2	3.633	812.59998	1068782.667	575598.6667
NA	HexCer 42:0;O3	12.967	812.70001	528051.6667	660531
NA	SL 49:8;O2	12.518	814.59998	383603.3333	358763.6667
NA	Cer 54:2;O3	13.871	814.79999	549496.6667	273272.3333
NA	SM 42:1;O2	14.343	815.70001	368108.3333	236616.3333
NA	SHexCer 36:5;O3	3.578	816.5	597929.6667	549496.3333
NA	SHexCer 36:5;O3	12.715	816.5	16359319.67	960713
NA	AHexCer 42:5;O3	3.578	816.59998	17445150.67	610944
NA	SE 24:1;O4	12.558	816.70001	588908.6667	378967
NA	BMP 39:10	11.653	818.5	576643.6667	684200
NA	LPC 38:4	13.863	818.59998	369617.3333	576277.6667
NA	Cer 53:1;O4	13.541	818.79999	478722.6667	557101
NA	PI 32:7	3.153	819.40002	1396910.333	616206
NA	PI 31:0	12.731	819.5	654023.3333	448645.6667
NA	SM 41:0;O3	12.282	819.70001	352511.3333	1475784.667
NA	HexCer 44:2;O2	3.011	820.70001	546799.6667	256513
NA	DG 50:11	4.977	821.59998	288473	679287
NA	Hex2Cer 32:7;O2	12.109	822.5	296436.6667	165388
NA	PC 39:5	4.576	822.59998	454821	463192.6667
NA	SL 49:4;O	5.276	823.70001	229764.6667	575323.6667
NA	SHexCer 38:7;O2	4.152	824.5	646056.3333	451472.6667
NA	HBMP 37:0	4.568	824.59998	351866.3333	541641
NA	AHexCer 43:0;O2	11.166	824.70001	336120.3333	562938
NA	TG 49:9	11.402	825.59998	426864.3333	726233
NA	SL 50:9;O2	14.303	826.59998	656217	676246
NA	SL 49:2;O2	12.51	826.70001	414456	945398.6667
NA	SM 43:2;O2	12.314	827.70001	570148.6667	3342527.667
NA	SHexCer 37:6;O3	15.003	828.5	693361	250240
NA	AHexCer 43:6;O3	14.995	828.59998	625751.6667	983559.6667
NA	TG 49:5	14.24	828.70001	670015	5038958.667
NA	TG 48:1	5.15	829.79999	374776.3333	528120.3333

NA	BMP 40:11	12.912	830.5	516081.6667	447996
NA	PC O-40:1	11.425	830.70001	480145.6667	11205404
NA	PI 33:8	6.896	831.40002	1500463.333	409784.3333
NA	PI 32:1	6.896	831.5	1392247.333	505402.3333
NA	SM 43:8;O3	6.896	831.59998	538205.6667	2140517.667
NA	SM 42:1;O3	3.098	831.70001	1097975.667	1722178.667
NA	PI 33:5	14.689	832.5	307173	1033229.667
NA	ASG 28:1;O	4.844	832.70001	366425.3333	15102833.67
NA	DG 51:12	12.817	833.59998	368200.3333	653668.6667
NA	DG 50:5	4.27	833.70001	678016	557881.3333
NA	PC 39:10	7.077	834.5	2401596.333	558251.6667
NA	SL 51:4;O	6.975	834.70001	2515740.667	622028
NA	SL 50:5;O	12.676	835.70001	486927.6667	339769.6667
NA	AHexCer 44:1;O2	14.044	836.70001	405415.3333	518339.6667
NA	TG 50:1	13.619	837.59998	637612.6667	529538.3333
NA	NAGly 50:1;O2	14.005	837.79999	260383.3333	442843.6667
NA	SL 50:3;O2	9.617	838.70001	352232	445026
NA	Cer 56:4;O3	12.558	838.79999	1019446.667	931042
NA	SM 43:7;O2	3.09	839.59998	994081.6667	12737892.67
NA	SM 44:3;O2	5.371	839.70001	328527.3333	382889.6667
NA	SHexCer 38:7;O3	3.688	840.5	2657775.333	852900.6667
NA	TG 50:6	13.415	840.70001	512048.6667	1408858.667
NA	PS 41:10	10.104	842.5	342044.6667	435922.6667
NA	PC O-41:2	3.499	842.70001	608160.6667	2190986
NA	Cer 55:3;O4	12.259	842.79999	387019	352186.6667
NA	PI 34:9	3.735	843.40002	2426417	448318
NA	PI 33:2	13.446	843.5	403861.3333	345749.3333
NA	SM 44:9;O3	5.363	843.59998	302985	458228.6667
NA	SM 43:2;O3	3.633	843.70001	1722633.667	267090
NA	ASG 28:2;O	13.737	844.70001	376573.3333	282119.3333
NA	DG 52:13	12.267	845.59998	380672.3333	206634.3333
NA	DG 51:6	12.259	845.70001	285337.3333	162652
NA	SL 52:5;O	12.77	846.70001	429848	1572255.667
NA	SL 51:6;O	14.767	847.70001	524614.6667	131825
NA	SHexCer 40:9;O2	11.323	848.5	746823	158002.3333
NA	AHexCer 45:2;O2	12.935	848.70001	379272.3333	300632
NA	NAGly 51:2;O2	12.078	849.79999	772271.3333	63625875.67
NA	Cer 57:5;O3	14.437	850.79999	564891.6667	516808.3333
NA	SM 44:8;O2	5.237	851.59998	307952.3333	487385.3333
NA	SM 44:8;O2	11.284	851.59998	213535.3333	257539.3333
NA	SM 45:4;O2	3.735	851.70001	1540367.667	221963.3333
NA	SHexCer 39:8;O3	14.083	852.5	403717.3333	474781.3333
NA	TG 51:7	14.571	852.70001	572321.6667	473211
NA	TG 50:0	14.225	852.79999	435263.3333	332712.3333
NA	PS 42:11	3.074	854.5	629202.6667	806648.6667
NA	SHexCer 38:0;O3	3.743	854.59998	861983	569218.3333

NA	PC O-42:3	3.051	854.70001	709012	433323.6667
NA	Cer 56:4;O4	3.743	854.79999	574587.6667	1211809.667
NA	Cer 56:4;O4	4.285	854.79999	682239.3333	501904.6667
NA	Cer 56:4;O4	4.655	854.79999	1898899.667	438774.6667
NA	SM 45:10;O3	3.027	855.59998	878741	762955
NA	PI 35:7	3.129	856.5	244260.6667	658788
NA	PI 35:7	12.267	856.5	594640.6667	462853
NA	PS 39:0	12.267	856.59998	421826.3333	2168633.667
NA	ASG 28:2;O	12.904	856.70001	437065.3333	4942857.667
NA	DG 53:14	3.035	857.59998	1576891.333	738397.6667
NA	DG 52:7	3.027	857.70001	406133.3333	739697
NA	PE 44:12	3.059	858.5	279963.3333	470784.3333
NA	PC 42:8	11.567	858.59998	542999.6667	670796
NA	SL 53:6;O	4.568	858.70001	508467.6667	608044
NA	Cer 58:0;O2	12.259	858.90002	439076	623868.6667
NA	SL 52:7;O	3.169	859.70001	328683.3333	633887.6667
NA	HBMP 40:3	11.535	860.59998	289954.3333	390921
NA	HBMP 40:3	12.691	860.59998	326279.3333	381306
NA	TG 52:12	11.944	861.59998	422357	659583.6667
NA	TG 51:5	14.594	861.70001	431516.3333	677229.6667
NA	NAGly 52:3;O2	14.673	861.79999	307631.3333	406127.6667
NA	TG 46:7;O4	3.145	862.59998	739633.3333	1680401.667
NA	SL 52:5;O2	3.137	862.70001	515172.6667	699531
NA	Cer 58:6;O3	5.3	862.79999	362324	390845.6667
NA	SM 45:9;O2	3.475	863.59998	356417	552489.6667
NA	TG 52:8	4.631	864.70001	424859.3333	567899.3333
NA	PS 41:9	6.149	866.5	268106	683768
NA	SHexCer 39:1;O3	6.314	866.59998	260213.3333	469403
NA	PC O-43:4	10.663	866.70001	394016	1760445.667
NA	Cer 57:5;O4	4.804	866.79999	320965.3333	1341542
NA	PI 35:4	4.521	867.5	452424.6667	498642.3333
NA	SM 45:4;O3	6.715	867.70001	222114	655509
NA	PI 36:8	12.133	868.5	1384226.667	275249.3333
NA	DGDG 29:0	13.029	868.59998	954927.6667	382132
NA	ASG 28:2;O	13.029	868.70001	936276.6667	331116.6667
NA	TG 50:0;O	4.065	868.79999	1213391.333	564700.3333
NA	DG 54:15	13.957	869.59998	920658.6667	620861
NA	DG 53:8	5.19	869.70001	850306	1548581.667
NA	PC 43:9	7.918	870.59998	562748.6667	571722
NA	SL 54:7;O	3.027	870.70001	425209.3333	1060106.667
NA	SL 54:7;O	13.729	870.70001	575153.6667	1023464.667
NA	SL 53:0;O	9.648	870.79999	540106.6667	509830.3333
NA	SL 53:8;O	13.32	871.70001	552213.6667	477596
NA	HBMP 41:4	5.095	872.59998	368949.3333	449907.6667
NA	AHexCer 47:4;O2	14.846	872.70001	675187	359798.6667
NA	TG 53:13	11.339	873.59998	358983.3333	573223

NA	TG 52:6	11.347	873.70001	359406	676463
NA	NAGly 53:4;O2	13.635	873.79999	221171.3333	488344.3333
NA	TG 47:8;O4	3.098	874.59998	663591.6667	875450
NA	SL 53:6;O2	14.366	874.70001	293374.3333	298135
NA	SL 53:6;O2	14.744	874.70001	581954.6667	312874.3333
NA	Cer 58:0;O3	3.027	874.90002	539887.6667	375830.3333
NA	TG 52:2	9.349	876.79999	190325.3333	513803.3333
NA	PS 42:10	5.253	878.5	398056	170521
NA	SHexCer 40:2;O3	9.184	878.59998	305850	332687.6667
NA	PC O-44:5	4.301	878.70001	326800	473121
NA	Cer 58:6;O4	13.43	878.79999	312425.3333	5410224.667
NA	PI 36:5	12.872	879.5	341669	730921
NA	SM 47:12;O3	3.633	879.59998	2430009.667	980541
NA	PI 37:9	10.277	880.5	200542.3333	132492
NA	HexCer 48:0;O2	3.735	880.79999	2559790.333	324864.6667
NA	DG O-54:0	5.08	880.90002	295641	318291
NA	DG 54:9	3.641	881.70001	2571282.667	338955.3333
NA	PC 44:10	3.578	882.59998	9660823.667	643350.6667
NA	SL 55:8;O	12.306	882.70001	387245	4766614.667
NA	SL 54:9;O	12.518	883.70001	339396	420164.6667
NA	AHexCer 48:5;O2	3.876	884.70001	1022997.333	557630.6667
NA	TG 54:14	12.613	885.59998	550506.6667	400992.6667
NA	TG 53:7	11.63	885.70001	496436.6667	502163.6667
NA	TG 48:9;O4	3.727	886.59998	238788.6667	1048617.667
NA	TG 48:9;O4	12.306	886.59998	1049482.333	1913266
NA	SL 54:7;O2	12.196	886.70001	225100.3333	748391
NA	Cer 60:8;O3	11.496	886.79999	483002	812054.6667
NA	Cer 59:1;O3	7.871	886.90002	521790	507686
NA	SM 48:7;O2	7.965	887.70001	3069036.333	461888
NA	AHexCer 48:11;O3	7.934	888.59998	1244065.333	931978
NA	TG 54:10	8.107	888.70001	847356	467206.6667
NA	PS 43:11	3.161	890.5	590327.6667	751804
NA	SHexCer 41:3;O3	5.158	890.59998	175698.3333	723881
NA	PC O-45:6	4.506	890.70001	396568.3333	933798
NA	Cer 59:7;O4	4.506	890.79999	317176	779890
NA	PI 37:6	13.58	891.5	289892.3333	908857
NA	SM 47:6;O3	14.484	891.70001	437492	765301.6667
NA	PI 38:10	7.84	892.5	1362497.333	697197
NA	DGDG 31:2	11.512	892.59998	348938.6667	894052
NA	BMP 43:1	14.712	892.70001	286768.3333	416118.6667
NA	HexCer 49:1;O2	5.276	892.79999	308559	1091445.667
NA	DG 55:10	3.625	893.70001	3162844.333	432343.6667
NA	PC 45:11	14.877	894.59998	649903	1136677.667
NA	SL 56:9;O	3.711	894.70001	6486825	757422.6667
NA	SL 55:2;O	14.185	894.79999	330074	1865293.667
NA	SE 28:1	10.183	894.90002	259838	1029905

NA	HBMP 43:6	5.732	896.59998	255275	521145
NA	AHexCer 49:6;O2	5.182	896.70001	422712	518894.3333
NA	NAGlySer 48:1;O2	14.932	896.79999	493382.6667	397120.6667
NA	TG 55:15	3.688	897.59998	7606396	536648.3333
NA	NAGly 55:6;O2	3.696	897.79999	3399667.667	333012
NA	SL 55:8;O2	4.262	898.70001	325749.6667	473960.6667
NA	SL 55:8;O2	7.266	898.70001	394689.3333	1076645.667
NA	SL 55:8;O2	11.866	898.70001	509346.6667	264071
NA	Cer 61:9;O3	3.947	898.79999	971133.6667	324568.6667
NA	Cer 60:2;O	3.947	898.90002	929319.6667	1520188.667
NA	SM 52:11;O3	12.534	951.70001	118528.3333	277578.3333
NA	DGDG 36:7	3.593	952.59998	815229.3333	3573030.667
NA	BMP 48:6	13.352	952.70001	173309.3333	158935.3333
NA	ASG 28:2;O	3.137	952.79999	307998.3333	112508.3333
NA	ASG 28:2;O	3.829	952.79999	704335	189578.3333
NA	Cer 63:4;O4	4.639	952.90002	170030.3333	125851.3333
NA	DG 60:15	3.161	953.70001	1015689.333	794737.6667
NA	Hex3Cer 29:1;O2	3.759	954.59998	519492	154346
NA	PI 41:0	3.483	954.70001	577635.6667	151080
NA	AHexCer 50:1;O3	5.811	954.79999	136486.3333	83779.66667
NA	AHexCer 50:1;O3	10.505	954.79999	224624.3333	329391.6667
NA	SE 28:2	12.243	954.90002	139781	167895.3333
NA	Cer 65:1;O2	4.12	955	206720	344221.6667
NA	DG 59:7	9.609	955.79999	174179	18878
NA	DG 58:0	12.746	955.90002	231889	66542
NA	HBMP 48:11	12.377	956.59998	157484.3333	465670.3333
NA	AHexCer 54:11;O2	3.145	956.70001	470338.6667	51116
NA	Cer 62:4;O4	14.39	956.90002	235245	3209140.667
NA	TG 59:13	13.839	957.70001	231995	77819.66667
NA	SL 58:0;O	3.161	957.90002	607595.6667	699495
NA	SHexCer 47:3;O2	3.019	958.70001	538472.6667	69380
NA	Cer 65:7;O3	5.646	958.90002	345616.3333	2653553.667
NA	TG 58:5	3.523	959.79999	442709	255925.3333
NA	TG 59:9	13.596	960.79999	365701	2998417.667
NA	TG 58:2	4.348	960.90002	384300	1039906.667
NA	Cer 64:0;O2	13.572	961	218332	1845613.667
NA	SL 58:6;O2	8.807	961.79999	130749	278924.3333
NA	SHexCer 47:9;O3	6.07	962.59998	181191	434447.3333
NA	PE O-54:12	10.183	962.70001	135213.3333	214373.3333
NA	ASG 27:1;O	11.189	964.79999	191836	229261.3333
NA	Cer 64:5;O4	7.847	964.90002	472011.3333	785433
NA	SM 52:4;O3	10.757	965.79999	99001	349878.6667
NA	Hex3Cer 30:2;O2	11.433	966.59998	179253.3333	516505.3333
NA	PI 42:1	8.736	966.70001	229771	546251
NA	AHexCer 51:2;O3	3.743	966.79999	1114621.333	290206
NA	Cer 67:9;O2	12.015	966.90002	262980.3333	470734.3333

NA	Cer 66:2;O2	5.04	967	165933	356606.6667
NA	DG 60:8	10.057	967.79999	121297	392380.6667
NA	DG 59:1	4.561	967.90002	241571.6667	980098
NA	HBMP 49:12	3.334	968.59998	604087.6667	799278.6667
NA	AHexCer 55:12;O2	11.653	968.70001	149869	293180.3333
NA	Cer 63:5;O4	3.648	968.90002	1137750.333	534718.3333
NA	TG 60:14	11.74	969.70001	210389.6667	514989.3333
NA	SHexCer 48:4;O2	11.276	970.70001	191264	490216
NA	TG 59:6	5.213	971.79999	289131.3333	924674
NA	TG 60:10	5.551	972.79999	194513	221749.3333
NA	TG 59:3	3.169	972.90002	773450	510649.6667
NA	Cer 65:0;O3	5.103	973	381066.3333	855637.6667
NA	SL 58:0;O2	3.633	973.90002	701400.3333	618116
NA	PC O-52:13	5.308	974.70001	156347	1065809.667
NA	AHexCer 53:3;O3	9.247	974.79999	163352.6667	608910
NA	SM 54:13;O3	3.884	975.70001	692890	1051659.667
NA	SM 54:13;O3	12.55	975.70001	152486	426596.3333
NA	DGDG 38:9	3.114	976.59998	253075.6667	277194
NA	BMP 50:8	12.424	976.70001	155867.6667	801824.6667
NA	ASG 28:2;O	12.424	976.79999	178224	774315.6667
NA	Cer 65:6;O4	12.416	976.90002	139004	458532
NA	Cer 64:0;O3	3.184	977	965514.6667	239742.3333
NA	PI 43:5	3.192	977.59998	1221757.333	724019.6667
NA	SM 53:5;O3	5.253	977.79999	148817.3333	672554
NA	HexCer 55:0;O2	13.438	978.90002	278768.6667	560736
NA	Cer 67:3;O2	14.406	979	395915	372309.6667
NA	DG 61:9	12.613	979.79999	294677.6667	378331.6667
NA	AHexCer 56:13;O2	5.355	980.70001	211133	550992.3333
NA	PC 48:0	5.74	980.79999	141544	282827
NA	Cer 64:6;O4	3.067	980.90002	291097	306897.3333
NA	TG 61:15	5.056	981.70001	223076	462505
NA	SHexCer 49:5;O2	3.672	982.70001	1732142.667	453910.3333
NA	HexCer 54:0;O2	9.318	982.90002	115120	537518
NA	TG 60:7	3.177	983.79999	611329	726426.6667
NA	TG 59:0	3.633	983.90002	122974.3333	430967.6667
NA	TG 59:0	6.031	983.90002	517128.6667	2672087.667
NA	Cer 66:1;O3	3.609	985	1395340.667	232138.3333
NA	SM 54:0;O2	7.792	985.90002	102919	583939
NA	PE O-56:14	14.594	986.70001	137469	162828.3333
NA	AHexCer 54:4;O3	12.809	986.79999	189370	799973
NA	SM 55:14;O3	3.444	987.70001	526392	179878
NA	DGDG 39:10	3.814	988.59998	166348	507533.6667
NA	PG 51:9	8.626	988.70001	182943.6667	336155
NA	Cer 66:7;O4	5.3	988.90002	165577	293158.3333
NA	PI 44:6	13.014	989.59998	115340.3333	232857.3333
NA	SM 54:6;O3	12.754	989.79999	122811	134966.3333

NA	Hex3Cer 32:4;O2	10.985	990.59998	166160	612619
NA	PI 44:3	11.197	990.70001	153315.6667	151083.3333
NA	AHexCer 53:4;O3	11.189	990.79999	156424	175422
NA	Cer 68:4;O2	5.567	991	127510.3333	205625.3333
NA	DG 62:10	4.27	991.79999	155384	725082
NA	DG 61:3	10.018	991.90002	90716.33333	566317.6667
NA	HBMP 51:14	4.222	992.59998	171298	369335.6667
NA	Cer 64:0;O4	13.478	993	127864.3333	3414973.667
NA	TG 62:1	12.267	993.70001	159740	305124
NA	TG 62:1	3.09	996.79999	693020	578601
NA	TG 62:12	3.664	996.79999	1052772.333	4974866.667
NA	TG 61:5	5.245	996.90002	187230	5890959.667
NA	Cer 65:0;O2	3.688	997	463860	257745
NA	SM 55:1;O2	2.705	997.90002	157513.6667	566690
NA	SHexCer 49:5;O3	11.921	998.70001	109305	4689909.667
NA	AHexCer 55:5;O3	4.946	998.79999	159074	227974
NA	SM 56:15;O3	3.963	999.70001	156307	636631.6667
NA	SM 56:15;O3	7.627	999.70001	405118.3333	1799789.667

Table S2: Significantly altered Metabolites (*P*<0.05).

Sr. No.	Metabolite Name	P-Value	-log 10 P
1	SL 28:0;O2	2.63E-12	11.579
2	Aminoadipic acid	5.97E-09	8.224
3	Tolmetin	6.53E-09	8.1853
4	6-Hydroxynicotinic acid	1.51E-08	7.8219
5	Glycerophosphocholine	6.11E-08	7.2137
6	N-Glycolylneuraminic acid	2.42E-07	6.6162
7	L-Cystathionine	4.49E-07	6.3482
8	Quinolinic Acid	4.87E-07	6.3123
9	Indole-3-acetyl-L-tryptophan	5.44E-07	6.2645
10	N-Acetylhistidine	8.95E-07	6.0481
11	Isopentenyladenine-9-N-glucoside	1.29E-06	5.8907
12	Leu-Leu-Tyr	1.61E-06	5.7934
13	VAE 17:4	2.44E-06	5.6129
14	Dimethylglycine	2.49E-06	5.6034
15	L-Thyronine	3.64E-06	5.4392
16	PI 12:0	6.82E-06	5.1659
17	TG 25:0;O2	6.89E-06	5.1616
18	Allocholic acid	7.52E-06	5.1238
19	SE 28:2.9	8.84E-06	5.0537
20	UDP-galactose	1.27E-05	4.896
21	VAE 21:3	1.54E-05	4.8119
22	N1-Acetylspermine	1.66E-05	4.7806
23	TG 55:15	2.19E-05	4.6588
24	DG 28:2	2.36E-05	4.6265
25	SHexCer 31:6;O2	2.40E-05	4.6194
26	DG 53:14	2.80E-05	4.5528
27	gamma-Taraxastane-3,20-diol	3.00E-05	4.5232
28	DG 29:3	3.30E-05	4.4809
29	PC 17:1	3.31E-05	4.4797
30	5-Methylcytosine	3.40E-05	4.469
31	SHexCer 29:4;O2	3.44E-05	4.4636
32	TG 26:1;O2	3.81E-05	4.4187
33	SL 44:5;O2	4.16E-05	4.3807
34	Cer 33:7;O4	4.19E-05	4.3775
35	TG 25:4	4.28E-05	4.3686
36	Uridine-5'-diphosphate sodium salt	4.49E-05	4.3478
37	L-beta-homoleucine-HCl	4.54E-05	4.3427
38	TG 29:4;O	4.65E-05	4.3323
39	PI 26:4.1	5.12E-05	4.2907
40	Cyclopamine	5.31E-05	4.2748
41	SM 44:8;O2.1	6.08E-05	4.2158
42	TG 34:7	6.52E-05	4.1857
43	Glycocholic acid	6.58E-05	4.1818

44	Ethylmalonic acid	7.34E-05	4.1341
45	PI 13:1	7.78E-05	4.109
46	Cer 27:1;O4	7.99E-05	4.0974
47	SL 36:3;O2.1	8.87E-05	4.0523
48	Cer 46:1;O4	9.05E-05	4.0434
49	Acetyl-CoA	9.50E-05	4.0222
50	Oxyacanthine	9.92E-05	4.0035
51	AHexCer 40:3;O3	0.00011257	3.9486
52	L-Asparagine	0.00011373	3.9441
53	PI 33:8	0.00011415	3.9425
54	SM 25:2;O2	0.00011971	3.9219
55	Thiamine monophosphate	0.0001239	3.9069
56	PI 32:1	0.00012687	3.8966
57	SL 26:0;O	0.00014034	3.8528
58	AHexCer 43:0;O2	0.00014109	3.8505
59	PI 35:7	0.00014143	3.8495
60	VAE 14:1	0.00014268	3.8456
61	AHexCer 42:5;O2	0.00015327	3.8145
62	1,2-Dipalmitoyl-rac-glycerol	0.00015661	3.8052
63	1-Methyladenosine	0.00015664	3.8051
64	VAE 16:3.1	0.00016753	3.7759
65	Ergosterol	0.00017245	3.7633
66	Sucralose	0.00017612	3.7542
67	Cotinine	0.0001783	3.7489
68	PI 44:6	0.00018555	3.7315
69	SE 24:1;O4.4	0.00018636	3.7297
70	DG 27:1	0.00019471	3.7106
71	NAGly 50:1;O2	0.00019626	3.7072
72	PI 43:5	0.00019896	3.7012
73	Z-Gly-Pro	0.00021068	3.6764
74	Adenylsuccinic acid	0.00022736	3.6433
75	AHexCer 51:2;O3	0.00022808	3.6419
76	HexCer 29:0;O4	0.0002331	3.6325
77	Cer 44:5;O4	0.00025113	3.6001
78	NAOrn 28:1	0.00026098	3.5834
79	Lysine	0.00027818	3.5557
80	Glucose 6-phosphate	0.00028828	3.5402
81	SL 28:2;O	0.0002931	3.533
82	VAE 13:0	0.00029627	3.5283
83	TG 28:1	0.00031299	3.5045
84	SM 36:1;O3	0.00031374	3.5034
85	L-Threonine	0.00034567	3.4613
86	PI 34:9	0.00037127	3.4303
87	Vindoline	0.00038497	3.4146
88	SL 39:6;O2	0.00040457	3.393
89	Cer 52:0;O3	0.00043664	3.3599

90	Biliverdin	0.00044098	3.3556
91	PS 28:2	0.00046595	3.3317
92	SM 55:1;O2	0.00046709	3.3306
93	SM 35:7;O2.1	0.00047293	3.3252
94	SL 34:0;O	0.00048923	3.3105
95	TG 27:0	0.00048988	3.3099
96	SHexCer 35:4;O2	0.00049456	3.3058
97	TG 62:12	0.00050163	3.2996
98	SL 52:5;O	0.00050236	3.299
99	SM 41:6;O3	0.00051319	3.2897
100	Cer 40:1;O4	0.00052312	3.2814
101	Hex2Cer 29:4;O2	0.00052929	3.2763
102	DG 60:15	0.0005524	3.2577
103	PS 35:9	0.00055879	3.2528
104	HBMP 49:12	0.00056259	3.2498
105	VAE 18:0	0.00059393	3.2263
106	SL 43:4;O2	0.00060082	3.2213
107	PC 36:7	0.00061812	3.2089
108	3',5'-Cyclic AMP	0.00066795	3.1753
109	Glutamylglutamic acid	0.00067623	3.1699
110	N-Palmitoyl-D-sphingosine	0.00067922	3.168
111	TG 40:9	0.00068127	3.1667
112	Cer 50:4;O4	0.00069246	3.1596
113	PG(16:0/16:0)	0.00070146	3.154
114	NAGly 42:12;O2	0.00070432	3.1522
115	PE 22:3	0.00070603	3.1512
116	HexCer 30:1;O2	0.00071855	3.1435
117	SM 38:3;O3	0.00072748	3.1382
118	Cer 47:8;O3	0.00073892	3.1314
119	NAGly 33:8;O2	0.0007491	3.1255
120	PI 25:3	0.00076831	3.1145
121	PE O-54:12	0.00077995	3.1079
122	DGDG 38:9	0.00078822	3.1034
123	Sanguinarine	0.00080743	3.0929
124	TG 50:0;O	0.00081327	3.0898
125	SM 45:10;O3	0.0008874	3.0519
126	L-alpha-Aminobutyric acid	0.00090158	3.045
127	PI 44:3	0.00090799	3.0419
128	Cer 32:0;O3	0.00091803	3.0371
129	AHexCer 48:11;O3	0.00093118	3.031
130	SHexCer 36:5;O3	0.00093855	3.0275
131	PS 42:11	0.00093984	3.0269
132	Adenine	0.00095747	3.0189
133	NAGly 40:4;O2	0.00095999	3.0177
134	Cer 60:2;O	0.0009791	3.0092
135	AHexCer 42:5;O3	0.0010662	2.9722

136	SHexCer 32:1;O2	0.0010818	2.9658
137	Cer 34:7;O4.1	0.0011039	2.9571
138	m-Coumaric acid	0.0011129	2.9535
139	SPB 24:0;O2	0.0011219	2.95
140	PC O-34:8	0.0011247	2.9489
141	Thyroxine	0.0011722	2.931
142	LPC 18:1	0.0011751	2.9299
143	SM 23:0;O2	0.0012109	2.9169
144	Argininosuccinic acid	0.001212	2.9165
145	SL 31:3;O2	0.0012131	2.9161
146	DG 31:5	0.0012283	2.9107
147	SL 55:8;O2	0.0012323	2.9093
148	PC 35:1	0.0012516	2.9025
149	PC 20:4	0.0012873	2.8903
150	Cer 46:0;O4	0.0012918	2.8888
151	Histidine	0.0013071	2.8837
152	SHexCer 26:1;O2	0.0013083	2.8833
153	HexCer 31:2;O4	0.001317	2.8804
154	Carnosine	0.0013398	2.873
155	SHexCer 47:3;O2	0.0013681	2.8639
156	SL 54:7;O.1	0.001376	2.8614
157	Cer 26:0;O4	0.0013902	2.8569
158	SL 38:5;O2	0.001401	2.8536
159	Purine	0.0014151	2.8492
160	Cer 63:4;O4	0.0014203	2.8476
161	VAE 26:7	0.0014318	2.8441
162	HexCer 38:9;O2	0.0014723	2.832
163	Urea	0.0014783	2.8302
164	AHexCer 43:6;O2	0.0014914	2.8264
165	SL 49:2;O2	0.001492	2.8262
166	PI 24:2	0.0014927	2.826
167	PI 37:9	0.0014984	2.8244
168	Glycyrrhetinic acid	0.0015035	2.8229
169	NAGly 44:8;O2	0.0015043	2.8227
170	PC O-43:4	0.0015308	2.8151
171	3,5-Diiodo-tyrosine	0.0015359	2.8136
172	DG 48:3	0.0015429	2.8117
173	Guanidinopropionic acid	0.0015849	2.8
174	AHexCer 48:5;O2	0.0016142	2.7921
175	Isoleucine	0.0016217	2.79
176	Hypoxanthine	0.0017203	2.7644
177	TG 59:0.1	0.0017585	2.7548
178	SHexCer 38:0;O3	0.0018119	2.7419
179	HexCer 41:0;O2	0.0018389	2.7354
180	TG 50:0	0.001858	2.731
181	NAGly 53:4;O2	0.001861	2.7303

182	Cer 35:2;O2	0.0018915	2.7232
183	SL 52:5;O2	0.0018954	2.7223
184	SM 44:3;O2	0.0019093	2.7191
185	HBMP 37:0	0.0019187	2.717
186	HexCer 29:0;O2	0.0019468	2.7107
187	AHexCer 40:3;O2	0.0020239	2.6938
188	Glutamic acid	0.0020879	2.6803
189	Cer 65:7;O3	0.0021224	2.6732
190	SL 58:0;O2	0.0021285	2.6719
191	Hex3Cer 29:1;O2	0.0022644	2.645
192	Cer 53:7;O4	0.0022659	2.6448
193	Cer 59:1;O3	0.0022739	2.6432
194	TG 42:2	0.0022818	2.6417
195	SL 52:7;O	0.0023089	2.6366
196	Dethiobiotin	0.0023277	2.6331
197	Cer 34:8;O4	0.0023298	2.6327
198	AHexCer 41:4;O2	0.0023527	2.6284
199	PI 38:10	0.0025277	2.5973
200	SE 28:2.8	0.0025344	2.5961
201	PI 31:0	0.0025531	2.5929
202	Methionine	0.0025656	2.5908
203	Homocarnosine	0.002666	2.5741
204	SL 35:2;O2	0.0026878	2.5706
205	TG 35:8	0.0027046	2.5679
206	PS 36:10	0.0027884	2.5546
207	Cer 32:6;O4	0.0028167	2.5503
208	LPC 24:3	0.002817	2.5502
209	Cer 65:0;O2	0.0028482	2.5454
210	PC O-52:13	0.0028763	2.5412
211	Cer 61:9;O3	0.0028794	2.5407
212	TG 58:2	0.0029735	2.5267
213	HexCer 31:2;O2	0.0030727	2.5125
214	PE 20:2	0.0031248	2.5052
215	Itaconic acid	0.0031339	2.5039
216	Cer 27:1;O2	0.0031361	2.5036
217	PS 14:1	0.0032074	2.4938
218	CAR 27:1	0.0032929	2.4824
219	SHexCer 40:9;O2	0.0033657	2.4729
220	PC O-29:3	0.003403	2.4681
221	PE 44:12	0.0034441	2.4629
222	HBMP 48:11	0.0035764	2.4466
223	PC 25:4	0.0035997	2.4437
224	(S)-4',7-Dihydroxy-3',8-diprenylflavanone	0.0036081	2.4427
225	PC O-20:0	0.0036548	2.4371
226	DGDG 31:2	0.0037493	2.426
227	PE 33:9	0.0037676	2.4239

228	Cer 55:3;O4	0.0037877	2.4216
229	NAE 4:0	0.003823	2.4176
230	Cer 60:8;O3	0.0038539	2.4141
231	Epigallocatechin gallate	0.003986	2.3995
232	PI 32:7	0.0039871	2.3993
233	NAGly 18:1;O2	0.0040825	2.3891
234	DG 61:3	0.0042346	2.3732
235	SM 55:14;O3	0.00426	2.3706
236	PC 43:9	0.0042674	2.3698
237	PC O-30:4	0.0042685	2.3697
238	SM 47:6;O3	0.0043004	2.3665
239	TG 39:2	0.0043074	2.3658
240	SL 45:4;O2	0.0043079	2.3657
241	SHexCer 41:3;O3	0.0043349	2.363
242	SL 47:2;O	0.0043557	2.3609
243	Cer 40:7;O4	0.0043861	2.3579
244	HexCer 44:2;O2	0.0044022	2.3563
245	HBMP 40:3.1	0.0044395	2.3527
246	SL 30:2;O2	0.0044874	2.348
247	7-Methylguanine	0.0044991	2.3469
248	SE 28:2.6	0.0045449	2.3425
249	SHexCer 33:8;O3	0.0045882	2.3384
250	SL 33:5;O2	0.0046007	2.3372
251	Inosine triphosphate	0.0046951	2.3284
252	TG 52:2	0.004711	2.3269
253	TG 59:3	0.0047302	2.3251
254	3-Man2GlcNAc	0.005184	2.2853
255	Brassicasterol	0.0051855	2.2852
256	N-Benzyl dimethyl stearyl ammonium	0.0051867	2.2851
257	DG 58:0	0.0053817	2.2691
258	NAOrn 40:12;O2	0.005408	2.267
259	DG 61:9	0.0056104	2.251
260	DG 36:4	0.0056135	2.2508
261	Cer 20:0;O3	0.0057846	2.2377
262	beta-Alanine	0.0058138	2.2355
263	SM 29:6;O2	0.0058228	2.2349
264	DG O-41:0	0.005853	2.2326
265	PC 42:8	0.005884	2.2303
266	Cer 46:7;O3	0.0060931	2.2152
267	AHexCer 39:2;O2	0.0062088	2.207
268	TG 47:8;O4	0.0062492	2.2042
269	N-Acetylneuraminate	0.0063122	2.1998
270	PS 42:10	0.0064464	2.1907
271	3-Hydroxykynurenine	0.0067146	2.173
272	PG 51:9	0.006826	2.1658
273	SHexCer 34:3;O3	0.0069142	2.1603

274	Berberine	0.0069988	2.155
275	AHexCer 50:1;O3.1	0.0071659	2.1447
276	DG 26:0	0.007393	2.1312
277	3-Hydroxybutyric acid	0.007452	2.1277
278	UDP-Galactose disodium salt	0.0075013	2.1249
279	Hex2Cer 32:7;O2	0.0075644	2.1212
280	PC 44:10	0.0078127	2.1072
281	PI 20:2	0.0078634	2.1044
282	Oxidized glutathione	0.0079826	2.1033
283	TG 48:9;O4.1	0.0080011	2.0969
284	BMP 24:1	0.0080572	2.0938
285	PC O-32:6	0.0082531	2.0834
286	Cer 56:4;O4.2	0.0083028	2.0808
287	LPC 38:4	0.0083213	2.0798
288	NAGly 22:6	0.0083336	2.0792
289	1,2-Dioleoyl-sn-glycero-3-phosphocholine	0.0087614	2.0574
290	Indoleacetic acid	0.0091211	2.04
291	SL 29:1;O2	0.0092228	2.0351
292	Cer 65:0;O3	0.0092797	2.0325
293	Cer 23:3;O2.1	0.0093201	2.0306
294	SL 55:8;O	0.010072	1.9969
295	AHexCer 38:2;O3	0.010282	1.9879
296	Cer 67:9;O2	0.010373	1.9841
297	HexCer 55:0;O2	0.010473	1.9799
298	DG 39:7	0.010719	1.9698
299	SM 28:5;O2	0.010727	1.9695
300	NAGly 40:10;O2	0.010742	1.9689
301	NAGly 43:7;O2	0.010795	1.9668
302	Stigmasterol	0.010849	1.9646
303	Cer 64:5;O4	0.010863	1.964
304	SHexCer 38:7;O2	0.011296	1.9471
305	TG 52:12	0.011438	1.9416
306	SHexCer 37:6;O2	0.011811	1.9277
307	Tryptophan	0.011992	1.9211
308	SL 54:7;O2	0.012344	1.9085
309	BMP 27:4	0.012672	1.8972
310	TG 58:5	0.012716	1.8957
311	Cer 53:8;O2	0.012795	1.8929
312	TG 46:7;O4	0.013421	1.8722
313	SL 36:8;O2	0.013542	1.8683
314	N-Fructosyl isoleucylglutamate	0.013934	1.8559
315	Cer 68:4;O2	0.013988	1.8542
316	Uridine diphosphategalactose	0.014143	1.8494
317	SL 37:9;O2	0.014314	1.8442
318	SL 51:4;O	0.014405	1.8415
319	SHexCer 26:1;O3	0.014442	1.8404

320	NAOrn 46:12;O2	0.014555	1.837
321	Isolithocholic acid	0.014899	1.8268
322	Cer 64:0;O3	0.014963	1.825
323	TG 60:14	0.014988	1.8243
324	TG 51:7	0.015039	1.8228
325	rac-B-tocopherol	0.015316	1.8149
326	S-Adenosylmethionine	0.015383	1.813
327	1-Stearoylglycerophosphocholine	0.015454	1.811
328	AHexCer 53:3;O3	0.015807	1.8011
329	TG 61:15	0.015871	1.7994
330	SHexCer 39:1;O3	0.015928	1.7979
331	CAR 20:0	0.016028	1.7951
332	Kynurenine	0.016135	1.7922
333	PC 31:2	0.016483	1.783
334	Palmatine	0.016605	1.7798
335	Valine	0.01712	1.7665
336	Cer 47:2;O4	0.017187	1.7648
337	N-Stearoyl tyrosine	0.017205	1.7643
338	Aspartylphenylalanine	0.017227	1.7638
339	CAR 28:2	0.017645	1.7534
340	SM 44:8;O2	0.0178	1.7496
341	SM 47:12;O3	0.018272	1.7382
342	PE O-40:11	0.018305	1.7374
343	PI 27:5	0.018327	1.7369

Table S3: Significantly altered Metabolites by Fold Change Analysis.

Sr. No.	Metabolite Name	Fold Change	log2(FC)
1	N-Acetylhistidine	15.653	3.9684
2	Isopentenyladenine-9-N-glucoside	0.083484	-3.5824
3	N-Glycolylneuraminic acid	0.10738	-3.2192
4	Glycerophosphocholine	0.15214	-2.7165
5	SL 44:5;O2	6.2685	2.6481
6	Leu-Leu-Tyr	0.17613	-2.5053
7	TG 25:0;O2	0.1778	-2.4917
8	L-Cystathionine	0.18843	-2.4079
9	N1-Acetylspermine	5.1344	2.3602
10	PI 12:0	0.20535	-2.2839
11	SL 28:0;O2	0.2132	-2.2297
12	Tolmetin	0.23829	-2.0692
13	TG 25:4	3.9858	1.9949
14	Quinolinic Acid	0.25555	-1.9683
15	DG 29:3	0.25505	-1.9712
16	PI 13:1	0.26218	-1.9314
17	DG 53:14	3.685	1.8816
18	SM 25:2;O2	3.6804	1.8799
19	Etodolac	3.4799	1.799
20	UDP-galactose	0.29734	-1.7498
21	PC 17:1	3.3208	1.7315
22	SE 24:1;O4.3	3.2948	1.7202
23	Z-Gly-Pro	0.30882	-1.6952
24	Adenylsuccinic acid	3.1251	1.6439
25	DG 28:2	0.32147	-1.6372
26	PS 35:9	3.1092	1.6365
27	Thiamine monophosphate	3.0172	1.5932
28	5-Methylcytosine	2.9739	1.5724
29	Cer 33:7;O4	2.9632	1.5671
30	TG 26:1;O2	2.9225	1.5472
31	SHexCer 29:4;O2	0.34371	-1.5408
32	Cer 27:1;O4	2.9055	1.5388
33	Allocholic acid	0.34853	-1.5207
34	SE 28:2.9	0.34956	-1.5164
35	HexCer 29:0;O4	2.8593	1.5157
36	TG 55:15	2.8466	1.5092
37	VAE 21:3	2.8012	1.4861
38	VAE 17:4	0.35774	-1.483
39	TG 29:4;O	0.35999	-1.474
40	HBMP 49:12	2.7199	1.4436
41	SL 36:3;O2.1	2.6499	1.4059
42	SL 34:0;O	2.6094	1.3837
43	Biliverdin	2.6037	1.3806

44	PI 26:4.1	2.5961	1.3764
45	6-Hydroxynicotinic acid	0.38838	-1.3645
46	gamma-Taraxastane-3,20-diol	0.39053	-1.3565
47	SM 44:8;O2.1	0.39791	-1.3295
48	PI 34:9	2.5107	1.3281
49	SE 24:1;O4.4	0.39905	-1.3254
50	PI 44:6	0.40093	-1.3186
51	SL 52:5;O	0.40548	-1.3023
52	SM 41:6;O3	0.41029	-1.2853
53	Cer 46:1;O4	0.41228	-1.2783
54	L-Threonine	0.41636	-1.2641
55	PI 35:7	0.41937	-1.2537
56	TG 34:7	0.41968	-1.2526
57	Vindoline	2.3601	1.2389
58	VAE 14:1	2.33	1.2203
59	NAGly 50:1;O2	0.43098	-1.2143
60	Uridine-5'-diphosphate sodium salt	0.43166	-1.212
61	PC 36:7	2.288	1.1941
62	Cyclopamine	0.43832	-1.1899
63	VAE 16:3.1	0.43872	-1.1886
64	HexCer 31:2;O4	2.2645	1.1792
65	DG 27:1	0.44724	-1.1609
66	AHexCer 42:5;O2	0.4533	-1.1415
67	AHexCer 43:0;O2	0.45479	-1.1367
68	PI 32:1	2.192	1.1322
69	SL 26:0;O	0.45779	-1.1272
70	TG 40:9	2.1769	1.1223
71	PE 33:9	2.1745	1.1207
72	VAE 18:0	0.46253	-1.1124
73	AHexCer 40:3;O3	0.46462	-1.1059
74	AHexCer 51:2;O3	2.1284	1.0898
75	Sanguinarine	2.1247	1.0873
76	Aminoadipic acid	0.47586	-1.0714
77	VAE 13:0	0.47701	-1.0679
78	PI 33:8	2.0858	1.0606
79	NAGly 18:1;O2	2.0793	1.0561
80	3-Hydroxybutyric acid	2.0738	1.0523
81	TG 28:1	0.4839	-1.0472
82	SL 28:2;O	0.49441	-1.0162
83	PI 43:5	2.0083	1.006
84	NAOrn 28:1	0.4995	-1.0015
85	Sucralose	0.50017	-0.99952
86	Cer 32:0;O3	1.996	0.99712
87	L-beta-homoleucine-HCl	0.50276	-0.99205
88	SL 52:5;O2	0.50336	-0.99033
89	SE 28:2.8	0.50921	-0.97366

90	Glycocholic acid	0.51351	-0.96153
91	LPC 18:1	0.51416	-0.95971
92	Ethylmalonic acid	0.51734	-0.95081
93	SHexCer 26:1;O2	0.51809	-0.94872
94	SL 55:8;O2	1.9103	0.93381
95	SHexCer 47:3;O2	1.9087	0.93257
96	DG 31:5	1.9076	0.93177
97	SHexCer 35:4;O2	0.52505	-0.92946
98	NAGly 42:12;O2	0.52699	-0.92416
99	N-Palmitoyl-D-sphingosine	1.8913	0.91939
100	1,2-Dipalmitoyl-rac-glycerol	0.52893	-0.91886
101	Cytarabine	1.888	0.91684
102	Cotinine	0.53046	-0.91469
103	PS 28:2	0.53067	-0.91412
104	Histidine	1.8828	0.91288
105	PI 31:0	1.8824	0.91259
106	SM 55:1;O2	1.8801	0.9108
107	SM 36:1;O3	0.53194	-0.91066
108	PC O-34:8	0.53208	-0.91029
109	DG 60:15	1.8777	0.90898
110	PC O-43:4	1.8735	0.90575
111	HexCer 41:0;O2	0.53591	-0.89993
112	Cer 40:1;O4	1.8595	0.89493
113	TG 62:12	1.859	0.89451
114	Cer 34:8;O4	1.8551	0.89153
115	SM 45:10;O3	1.854	0.89067
116	Acetyl-CoA	0.54203	-0.88355
117	Oxyacanthine	0.5428	-0.88152
118	Glucose 6-phosphate	0.54563	-0.87401
119	Cer 52:0;O3	0.54714	-0.87002
120	SL 39:6;O2	1.8257	0.86848
121	TG 50:0	1.8155	0.8604
122	DGDG 38:9	0.55185	-0.85766
123	AHexCer 39:2;O2	1.7966	0.84527
124	Dethiobiotin	1.7959	0.84467
125	S-Adenosylmethionine	1.7954	0.84434
126	SM 35:7;O2.1	0.55928	-0.83836
127	Ergosterol	0.56051	-0.8352
128	Hex2Cer 29:4;O2	0.56295	-0.82892
129	SL 43:4;O2	0.56893	-0.81368
130	DG 61:3	0.56963	-0.8119
131	Cer 60:2;O	1.7437	0.80212
132	3-Man2GlcNAc	1.7403	0.79933
133	Indole-3-acetyl-L-tryptophan	0.5755	-0.7971
134	NAOrn 40:12;O2	1.7339	0.79403
135	UDP-Galactose disodium salt	1.7303	0.79099

136	HexCer 30:1;O2	0.57829	-0.79014
137	SL 31:3;O2	0.57833	-0.79003
138	Cer 34:7;O4.1	0.57871	-0.7891
139	Argininosuccinic acid	1.7254	0.78696
140	Cer 35:2;O2	1.7232	0.78508
141	N-Acetylneuraminate	1.7219	0.78399
142	N-Benzyl dimethyl stearyl ammonium	1.7215	0.78366
143	Glutamic acid	1.719	0.78153
144	Cer 50:4;O4	0.58266	-0.77926
145	PI 44:3	1.7157	0.77878
146	DG 58:0	1.7107	0.77462
147	m-Coumaric acid	1.7092	0.77332
148	Isolithocholic acid	1.7068	0.7713
149	PC 25:4	1.7006	0.76607
150	Hex3Cer 29:1;O2	1.7002	0.76566
151	PI 25:3	0.58842	-0.76509
152	PI 37:9	0.58897	-0.76374
153	Cer 63:4;O4	0.58957	-0.76225
154	Cer 59:1;O3	1.6935	0.75997
155	SHexCer 31:6;O2	0.59247	-0.75519
156	Cer 26:0;O4	1.6855	0.75315
157	DG 48:3	1.685	0.75275
158	PE 20:2	0.59423	-0.75092
159	Cer 47:8;O3	0.59527	-0.74838
160	NAGly 22:6	1.6783	0.74703
161	TG 50:0;O	1.6759	0.74494
162	SM 23:0;O2	0.59761	-0.74272
163	Thyroxine	1.6695	0.73944
164	PC O-20:0	0.59926	-0.73873
165	NAOrn 15:0;O	1.6631	0.73387
166	PS 14:1	1.6602	0.73132
167	Cer 44:5;O4	0.60253	-0.7309
168	SM 38:3;O3	0.60255	-0.73086
169	HexCer 38:9;O2	0.60261	-0.7307
170	PE O-54:12	0.60276	-0.73035
171	Purine	1.6556	0.72734
172	TG 58:2	1.6555	0.72725
173	NAGly 40:4;O2	0.60409	-0.72717
174	SHexCer 32:1;O2	0.60454	-0.72609
175	Cer 56:4;O4.2	1.653	0.72507
176	SPB 24:0;O2	0.60924	-0.71491
177	7-Methylguanine	1.6411	0.71469
178	SHexCer 38:0;O3	0.61183	-0.7088
179	NAGly 44:8;O2	0.61228	-0.70774
180	AHexCer 41:4;O2	0.61279	-0.70654
181	SHexCer 40:9;O2	1.6298	0.7047

182	Cer 32:6;O4	0.61414	-0.70335
183	PC O-30:4	1.6264	0.70171
184	SL 51:4;O	1.6245	0.70004
185	DG 26:0	0.6157	-0.6997
186	AHexCer 48:11;O3	1.6196	0.69564
187	Cer 60:8;O3	1.6188	0.69493
188	Cer 46:0;O4	1.6138	0.69047
189	DGDG 31:2	1.6118	0.68869
190	PC 35:1	0.62194	-0.68515
191	Cer 27:1;O2	0.62218	-0.6846
192	Cer 40:7;O4	1.6055	0.68301
193	SM 47:12;O3	1.5985	0.67671
194	SM 55:14;O3	1.5981	0.67633
195	HexCer 31:2;O2	1.5965	0.67494
196	Adenine	0.62727	-0.67284
197	Indoleacetic acid	1.5888	0.66792
198	HexCer 44:2;O2	1.5883	0.66749
199	TG 47:8;O4	0.63099	-0.6643
200	Cer 49:4;O4	0.63177	-0.66254
201	Inosine triphosphate	1.5818	0.66161
202	SL 49:2;O2	0.63227	-0.6614
203	Cer 55:3;O4	1.5812	0.66104
204	Lysine	0.63263	-0.66056
205	SM 44:3;O2	0.63313	-0.65943
206	Homocarnosine	1.5778	0.65789
207	L-Asparagine	0.6341	-0.65722
208	SM 47:6;O3	1.5761	0.65636
209	SL 38:5;O2	0.63488	-0.65545
210	1-Methyladenosine	0.6363	-0.65222
211	SHexCer 33:8;O3	1.5672	0.64816
212	SL 30:2;O2	1.5663	0.64738
213	Cer 53:8;O2	1.5644	0.64561
214	Urea	0.63976	-0.64441
215	AHexCer 48:5;O2	1.5625	0.64385
216	CAR 27:1	0.64136	-0.64079
217	TG 60:14	1.5588	0.64046
218	SHexCer 36:5;O3	0.64222	-0.63887
219	3,5-Diiodo-tyrosine	0.64307	-0.63695
220	SL 52:7;O	0.64469	-0.63332
221	3',5'-Cyclic AMP	0.64488	-0.63289
222	Berberine	0.6449	-0.63285
223	AHexCer 43:6;O2	0.6458	-0.63084
224	PE 44:12	0.64586	-0.63071
225	TG 59:0.1	0.64643	-0.62943
226	TG 27:0	1.5456	0.62815
227	BMP 24:1	1.5425	0.6253

228	SE 24:1;O4	1.5424	0.62522
229	PG 51:9	1.5402	0.62316
230	HBMP 37:0	0.64931	-0.62302
231	DG 61:9	1.5354	0.61857
232	AHexCer 40:3;O2	0.65136	-0.61847
233	NAGly 53:4;O2	0.65412	-0.61238
234	PS 42:11	0.65493	-0.61058
235	Guanidinopropionic acid	1.5253	0.60905
236	TG 59:3	1.5239	0.6078
237	PC 42:8	1.5231	0.60705
238	SL 54:7;O.1	0.65698	-0.60608
239	PG(16:0/16:0)	0.6576	-0.60471
240	SHexCer 41:3;O3	0.65782	-0.60423
241	HexCer 29:0;O2	0.66089	-0.59751
242	TG 51:7	1.5128	0.59717
243	VAE 26:7	0.66152	-0.59614
244	gamma-Tocotrienol	1.5069	0.59155
245	Creatinine	1.5033	0.58817

Table S4: Significantly altered Metabolites by P-Value and Fold Change Analysis.

Sr. No.	Metabolite Name	Fold Change	log2(FC)	p value	-log 10 p
1	SL 28:0;O2	0.2132	-2.2297	2.63E-12	11.579
2	Aminoadipic acid	0.47586	-1.0714	5.97E-09	8.224
3	Tolmetin	0.23829	-2.0692	6.53E-09	8.1853
4	6-Hydroxynicotinic acid	0.38838	-1.3645	1.51E-08	7.8219
5	Glycerophosphocholine	0.15214	-2.7165	6.11E-08	7.2137
6	N-Glycolylneuraminic acid	0.10738	-3.2192	2.42E-07	6.6162
7	L-Cystathionine	0.18843	-2.4079	4.49E-07	6.3482
8	Quinolinic Acid	0.25555	-1.9683	4.87E-07	6.3123
9	Indole-3-acetyl-L-tryptophan	0.5755	-0.7971	5.44E-07	6.2645
10	N-Acetylhistidine	15.653	3.9684	8.95E-07	6.0481
11	Isopentenyladenine-9-N-glucoside	0.083484	-3.5824	1.29E-06	5.8907
12	Leu-Leu-Tyr	0.17613	-2.5053	1.61E-06	5.7934
13	VAE 17:4	0.35774	-1.483	2.44E-06	5.6129
14	PI 12:0	0.20535	-2.2839	6.82E-06	5.1659
15	TG 25:0;O2	0.1778	-2.4917	6.89E-06	5.1616
16	Allocholic acid	0.34853	-1.5207	7.52E-06	5.1238
17	SE 28:2.9	0.34956	-1.5164	8.84E-06	5.0537
18	UDP-galactose	0.29734	-1.7498	1.27E-05	4.896
19	VAE 21:3	2.8012	1.4861	1.54E-05	4.8119
20	N1-Acetylspermine	5.1344	2.3602	1.66E-05	4.7806
21	TG 55:15	2.8466	1.5092	2.19E-05	4.6588
22	DG 28:2	0.32147	-1.6372	2.36E-05	4.6265
23	SHexCer 31:6;O2	0.59247	-0.75519	2.40E-05	4.6194
24	DG 53:14	3.685	1.8816	2.80E-05	4.5528
25	gamma-Taraxastane-3,20-diol	0.39053	-1.3565	3.00E-05	4.5232
26	DG 29:3	0.25505	-1.9712	3.30E-05	4.4809
27	PC 17:1	3.3208	1.7315	3.31E-05	4.4797
28	5-Methylcytosine	2.9739	1.5724	3.40E-05	4.469
29	SHexCer 29:4;O2	0.34371	-1.5408	3.44E-05	4.4636
30	TG 26:1;O2	2.9225	1.5472	3.81E-05	4.4187
31	SL 44:5;O2	6.2685	2.6481	4.16E-05	4.3807
32	Cer 33:7;O4	2.9632	1.5671	4.19E-05	4.3775
33	TG 25:4	3.9858	1.9949	4.28E-05	4.3686
34	Uridine-5'-diphosphate sodium salt	0.43166	-1.212	4.49E-05	4.3478
35	L-beta-homoleucine-HCl	0.50276	-0.99205	4.54E-05	4.3427
36	TG 29:4;O	0.35999	-1.474	4.65E-05	4.3323
37	PI 26:4.1	2.5961	1.3764	5.12E-05	4.2907
38	Cyclopamine	0.43832	-1.1899	5.31E-05	4.2748
39	SM 44:8;O2.1	0.39791	-1.3295	6.08E-05	4.2158
40	TG 34:7	0.41968	-1.2526	6.52E-05	4.1857
41	Glycocholic acid	0.51351	-0.96153	6.58E-05	4.1818
42	Ethylmalonic acid	0.51734	-0.95081	7.34E-05	4.1341
43	PI 13:1	0.26218	-1.9314	7.78E-05	4.109
44	Cer 27:1;O4	2.9055	1.5388	7.99E-05	4.0974

45	SL 36:3;O2.1	2.6499	1.4059	8.87E-05	4.0523
46	Cer 46:1;O4	0.41228	-1.2783	9.05E-05	4.0434
47	Acetyl-CoA	0.54203	-0.88355	9.50E-05	4.0222
48	Oxyacanthine	0.5428	-0.88152	9.92E-05	4.0035
49	AHexCer 40:3;O3	0.46462	-1.1059	0.00011257	3.9486
50	L-Asparagine	0.6341	-0.65722	0.00011373	3.9441
51	PI 33:8	2.0858	1.0606	0.00011415	3.9425
52	SM 25:2;O2	3.6804	1.8799	0.00011971	3.9219
53	Thiamine monophosphate	3.0172	1.5932	0.0001239	3.9069
54	PI 32:1	2.192	1.1322	0.00012687	3.8966
55	SL 26:0;O	0.45779	-1.1272	0.00014034	3.8528
56	AHexCer 43:0;O2	0.45479	-1.1367	0.00014109	3.8505
57	PI 35:7	0.41937	-1.2537	0.00014143	3.8495
58	VAE 14:1	2.33	1.2203	0.00014268	3.8456
59	AHexCer 42:5;O2	0.4533	-1.1415	0.00015327	3.8145
60	1,2-Dipalmitoyl-rac-glycerol	0.52893	-0.91886	0.00015661	3.8052
61	1-Methyladenosine	0.6363	-0.65222	0.00015664	3.8051
62	VAE 16:3.1	0.43872	-1.1886	0.00016753	3.7759
63	Ergosterol	0.56051	-0.8352	0.00017245	3.7633
64	Sucralose	0.50017	-0.99952	0.00017612	3.7542
65	Cotinine	0.53046	-0.91469	0.0001783	3.7489
66	PI 44:6	0.40093	-1.3186	0.00018555	3.7315
67	SE 24:1;O4.4	0.39905	-1.3254	0.00018636	3.7297
68	DG 27:1	0.44724	-1.1609	0.00019471	3.7106
69	NAGly 50:1;O2	0.43098	-1.2143	0.00019626	3.7072
70	PI 43:5	2.0083	1.006	0.00019896	3.7012
71	Z-Gly-Pro	0.30882	-1.6952	0.00021068	3.6764
72	Adenylsuccinic acid	3.1251	1.6439	0.00022736	3.6433
73	AHexCer 51:2;O3	2.1284	1.0898	0.00022808	3.6419
74	HexCer 29:0;O4	2.8593	1.5157	0.0002331	3.6325
75	Cer 44:5;O4	0.60253	-0.7309	0.00025113	3.6001
76	NAOrn 28:1	0.4995	-1.0015	0.00026098	3.5834
77	Lysine	0.63263	-0.66056	0.00027818	3.5557
78	Glucose 6-phosphate	0.54563	-0.87401	0.00028828	3.5402
79	SL 28:2;O	0.49441	-1.0162	0.0002931	3.533
80	VAE 13:0	0.47701	-1.0679	0.00029627	3.5283
81	TG 28:1	0.4839	-1.0472	0.00031299	3.5045
82	SM 36:1;O3	0.53194	-0.91066	0.00031374	3.5034
83	L-Threonine	0.41636	-1.2641	0.00034567	3.4613
84	PI 34:9	2.5107	1.3281	0.00037127	3.4303
85	Vindoline	2.3601	1.2389	0.00038497	3.4146
86	SL 39:6;O2	1.8257	0.86848	0.00040457	3.393
87	Cer 52:0;O3	0.54714	-0.87002	0.00043664	3.3599
88	Biliverdin	2.6037	1.3806	0.00044098	3.3556
89	PS 28:2	0.53067	-0.91412	0.00046595	3.3317
90	SM 55:1;O2	1.8801	0.9108	0.00046709	3.3306

91	SM 35:7;O2.1	0.55928	-0.83836	0.00047293	3.3252
92	SL 34:0;O	2.6094	1.3837	0.00048923	3.3105
93	TG 27:0	1.5456	0.62815	0.00048988	3.3099
94	SHexCer 35:4;O2	0.52505	-0.92946	0.00049456	3.3058
95	TG 62:12	1.859	0.89451	0.00050163	3.2996
96	SL 52:5;O	0.40548	-1.3023	0.00050236	3.299
97	SM 41:6;O3	0.41029	-1.2853	0.00051319	3.2897
98	Cer 40:1;O4	1.8595	0.89493	0.00052312	3.2814
99	Hex2Cer 29:4;O2	0.56295	-0.82892	0.00052929	3.2763
100	DG 60:15	1.8777	0.90898	0.0005524	3.2577
101	PS 35:9	3.1092	1.6365	0.00055879	3.2528
102	HBMP 49:12	2.7199	1.4436	0.00056259	3.2498
103	VAE 18:0	0.46253	-1.1124	0.00059393	3.2263
104	SL 43:4;O2	0.56893	-0.81368	0.00060082	3.2213
105	PC 36:7	2.288	1.1941	0.00061812	3.2089
106	3',5'-Cyclic AMP	0.64488	-0.63289	0.00066795	3.1753
107	N-Palmitoyl-D-sphingosine	1.8913	0.91939	0.00067922	3.168
108	TG 40:9	2.1769	1.1223	0.00068127	3.1667
109	Cer 50:4;O4	0.58266	-0.77926	0.00069246	3.1596
110	PG(16:0/16:0)	0.6576	-0.60471	0.00070146	3.154
111	NAGly 42:12;O2	0.52699	-0.92416	0.00070432	3.1522
112	HexCer 30:1;O2	0.57829	-0.79014	0.00071855	3.1435
113	SM 38:3;O3	0.60255	-0.73086	0.00072748	3.1382
114	Cer 47:8;O3	0.59527	-0.74838	0.00073892	3.1314
115	PI 25:3	0.58842	-0.76509	0.00076831	3.1145
116	PE O-54:12	0.60276	-0.73035	0.00077995	3.1079
117	DGDG 38:9	0.55185	-0.85766	0.00078822	3.1034
118	Sanguinarine	2.1247	1.0873	0.00080743	3.0929
119	TG 50:0;O	1.6759	0.74494	0.00081327	3.0898
120	SM 45:10;O3	1.854	0.89067	0.0008874	3.0519
121	PI 44:3	1.7157	0.77878	0.00090799	3.0419
122	Cer 32:0;O3	1.996	0.99712	0.00091803	3.0371
123	AHexCer 48:11;O3	1.6196	0.69564	0.00093118	3.031
124	SHexCer 36:5;O3	0.64222	-0.63887	0.00093855	3.0275
125	PS 42:11	0.65493	-0.61058	0.00093984	3.0269
126	Adenine	0.62727	-0.67284	0.00095747	3.0189
127	NAGly 40:4;O2	0.60409	-0.72717	0.00095999	3.0177
128	Cer 60:2;O	1.7437	0.80212	0.0009791	3.0092
129	SHexCer 32:1;O2	0.60454	-0.72609	0.0010818	2.9658
130	Cer 34:7;O4.1	0.57871	-0.7891	0.0011039	2.9571
131	m-Coumaric acid	1.7092	0.77332	0.0011129	2.9535
132	SPB 24:0;O2	0.60924	-0.71491	0.0011219	2.95
133	PC O-34:8	0.53208	-0.91029	0.0011247	2.9489
134	Thyroxine	1.6695	0.73944	0.0011722	2.931
135	LPC 18:1	0.51416	-0.95971	0.0011751	2.9299
136	SM 23:0;O2	0.59761	-0.74272	0.0012109	2.9169

137	Argininosuccinic acid	1.7254	0.78696	0.001212	2.9165
138	SL 31:3;O2	0.57833	-0.79003	0.0012131	2.9161
139	DG 31:5	1.9076	0.93177	0.0012283	2.9107
140	SL 55:8;O2	1.9103	0.93381	0.0012323	2.9093
141	PC 35:1	0.62194	-0.68515	0.0012516	2.9025
142	Cer 46:0;O4	1.6138	0.69047	0.0012918	2.8888
143	Histidine	1.8828	0.91288	0.0013071	2.8837
144	SHexCer 26:1;O2	0.51809	-0.94872	0.0013083	2.8833
145	HexCer 31:2;O4	2.2645	1.1792	0.001317	2.8804
146	SHexCer 47:3;O2	1.9087	0.93257	0.0013681	2.8639
147	SL 54:7;O.1	0.65698	-0.60608	0.001376	2.8614
148	Cer 26:0;O4	1.6855	0.75315	0.0013902	2.8569
149	SL 38:5;O2	0.63488	-0.65545	0.001401	2.8536
150	Purine	1.6556	0.72734	0.0014151	2.8492
151	Cer 63:4;O4	0.58957	-0.76225	0.0014203	2.8476
152	VAE 26:7	0.66152	-0.59614	0.0014318	2.8441
153	HexCer 38:9;O2	0.60261	-0.7307	0.0014723	2.832
154	Urea	0.63976	-0.64441	0.0014783	2.8302
155	AHexCer 43:6;O2	0.6458	-0.63084	0.0014914	2.8264
156	SL 49:2;O2	0.63227	-0.6614	0.001492	2.8262
157	PI 37:9	0.58897	-0.76374	0.0014984	2.8244
158	NAGly 44:8;O2	0.61228	-0.70774	0.0015043	2.8227
159	PC O-43:4	1.8735	0.90575	0.0015308	2.8151
160	3,5-Diiodo-tyrosine	0.64307	-0.63695	0.0015359	2.8136
161	DG 48:3	1.685	0.75275	0.0015429	2.8117
162	Guanidinopropionic acid	1.5253	0.60905	0.0015849	2.8
163	AHexCer 48:5;O2	1.5625	0.64385	0.0016142	2.7921
164	Cer 49:4;O4	0.63177	-0.66254	0.0017203	2.7644
165	TG 59:0.1	0.64643	-0.62943	0.0017585	2.7548
166	SHexCer 38:0;O3	0.61183	-0.7088	0.0018119	2.7419
167	HexCer 41:0;O2	0.53591	-0.89993	0.0018389	2.7354
168	TG 50:0	1.8155	0.8604	0.001858	2.731
169	NAGly 53:4;O2	0.65412	-0.61238	0.001861	2.7303
170	Cer 35:2;O2	1.7232	0.78508	0.0018915	2.7232
171	SL 52:5;O2	0.50336	-0.99033	0.0018954	2.7223
172	SM 44:3;O2	0.63313	-0.65943	0.0019093	2.7191
173	HBMP 37:0	0.64931	-0.62302	0.0019187	2.717
174	HexCer 29:0;O2	0.66089	-0.59751	0.0019468	2.7107
175	AHexCer 40:3;O2	0.65136	-0.61847	0.0020239	2.6938
176	Glutamic acid	1.719	0.78153	0.0020879	2.6803
177	Hex3Cer 29:1;O2	1.7002	0.76566	0.0022644	2.645
178	Cer 59:1;O3	1.6935	0.75997	0.0022739	2.6432
179	SL 52:7;O	0.64469	-0.63332	0.0023089	2.6366
180	Dethiobiotin	1.7959	0.84467	0.0023277	2.6331
181	Cer 34:8;O4	1.8551	0.89153	0.0023298	2.6327
182	AHexCer 41:4;O2	0.61279	-0.70654	0.0023527	2.6284

183	SE 28:2.8	0.50921	-0.97366	0.0025344	2.5961
184	PI 31:0	1.8824	0.91259	0.0025531	2.5929
185	SE 24:1;O4	1.5424	0.62522	0.0025656	2.5908
186	Homocarnosine	1.5778	0.65789	0.002666	2.5741
187	Cer 32:6;O4	0.61414	-0.70335	0.0028167	2.5503
188	TG 58:2	1.6555	0.72725	0.0029735	2.5267
189	HexCer 31:2;O2	1.5965	0.67494	0.0030727	2.5125
190	PE 20:2	0.59423	-0.75092	0.0031248	2.5052
191	Cer 27:1;O2	0.62218	-0.6846	0.0031361	2.5036
192	PS 14:1	1.6602	0.73132	0.0032074	2.4938
193	CAR 27:1	0.64136	-0.64079	0.0032929	2.4824
194	SHexCer 40:9;O2	1.6298	0.7047	0.0033657	2.4729
195	PE 44:12	0.64586	-0.63071	0.0034441	2.4629
196	PC 25:4	1.7006	0.76607	0.0035997	2.4437
197	PC O-20:0	0.59926	-0.73873	0.0036548	2.4371
198	DGDG 31:2	1.6118	0.68869	0.0037493	2.426
199	PE 33:9	2.1745	1.1207	0.0037676	2.4239
200	Cer 55:3;O4	1.5812	0.66104	0.0037877	2.4216
201	Cer 60:8;O3	1.6188	0.69493	0.0038539	2.4141
202	NAGly 18:1;O2	2.0793	1.0561	0.0040825	2.3891
203	DG 61:3	0.56963	-0.8119	0.0042346	2.3732
204	SM 55:14;O3	1.5981	0.67633	0.00426	2.3706
205	PC O-30:4	1.6264	0.70171	0.0042685	2.3697
206	SM 47:6;O3	1.5761	0.65636	0.0043004	2.3665
207	SHexCer 41:3;O3	0.65782	-0.60423	0.0043349	2.363
208	Cer 40:7;O4	1.6055	0.68301	0.0043861	2.3579
209	HexCer 44:2;O2	1.5883	0.66749	0.0044022	2.3563
210	SL 30:2;O2	1.5663	0.64738	0.0044874	2.348
211	7-Methylguanine	1.6411	0.71469	0.0044991	2.3469
212	SHexCer 33:8;O3	1.5672	0.64816	0.0045882	2.3384
213	Inosine triphosphate	1.5818	0.66161	0.0046951	2.3284
214	TG 59:3	1.5239	0.6078	0.0047302	2.3251
215	3-Man2GlcNAc	1.7403	0.79933	0.005184	2.2853
216	N-Benzyl dimethyl stearyl ammonium	1.7215	0.78366	0.0051867	2.2851
217	DG 58:0	1.7107	0.77462	0.0053817	2.2691
218	NAOrn 40:12;O2	1.7339	0.79403	0.005408	2.267
219	DG 61:9	1.5354	0.61857	0.0056104	2.251
220	Cytarabine	1.888	0.91684	0.0058138	2.2355
221	PC 42:8	1.5231	0.60705	0.005884	2.2303
222	AHexCer 39:2;O2	1.7966	0.84527	0.0062088	2.207
223	TG 47:8;O4	0.63099	-0.6643	0.0062492	2.2042
224	N-Acetylneuraminate	1.7219	0.78399	0.0063122	2.1998
225	PG 51:9	1.5402	0.62316	0.006826	2.1658
226	Berberine	0.6449	-0.63285	0.0069988	2.155
227	DG 26:0	0.6157	-0.6997	0.007393	2.1312
228	3-Hydroxybutyric acid	2.0738	1.0523	0.007452	2.1277

229	UDP-Galactose disodium salt	1.7303	0.79099	0.0075013	2.1249
230	BMP 24:1	1.5425	0.6253	0.0080572	2.0938
231	Cer 56:4;O4.2	1.653	0.72507	0.0083028	2.0808
232	NAGly 22:6	1.6783	0.74703	0.0083336	2.0792
233	Indoleacetic acid	1.5888	0.66792	0.0091211	2.04
234	Cer 53:8;O2	1.5644	0.64561	0.012795	1.8929
235	SL 51:4;O	1.6245	0.70004	0.014405	1.8415
236	Isolithocholic acid	1.7068	0.7713	0.014899	1.8268
237	TG 60:14	1.5588	0.64046	0.014988	1.8243
238	TG 51:7	1.5128	0.59717	0.015039	1.8228
239	S-Adenosylmethionine	1.7954	0.84434	0.015383	1.813
240	SM 47:12;O3	1.5985	0.67671	0.018272	1.7382
241	gamma-Tocotrienol	1.5069	0.59155	0.020398	1.6904
242	Creatinine	1.5033	0.58817	0.026559	1.5758
243	NAOrn 15:0;O	1.6631	0.73387	0.033802	1.4711

Table S5: Enriched Diseases identified as significant (P-value was less than 0.05).

Sr. No.	Disease	Total metabolites	Expected	Hits	P-Value
1	Uremia	92	0.125	7	3.09E-11
2	Colorectal cancer	54	0.0731	5	9.56E-09
3	Dimethylglycine Dehydrogenase Deficiency	8	0.0108	3	1.24E-07
4	Schizophrenia	101	0.137	5	2.29E-07
5	Alzheimer's disease	59	0.0799	4	1.17E-06
6	Alpha-aminoadipic and alpha-ketoadipic aciduria	2	0.00271	2	1.77E-06
7	Pancreatic cancer	21	0.0284	3	2.92E-06
8	Kidney disease	28	0.0379	3	7.15E-06
9	Sitosterolemia	5	0.00677	2	1.77E-05
10	Bartter Syndrome, Type 4B, Neonatal, With Sensorineural Deafness	8	0.0108	2	4.93E-05
11	Stomach cancer	9	0.0122	2	6.34E-05
12	Bartter Syndrome, Type 2, Antenatal	10	0.0135	2	7.91E-05
13	Ovarian cancer	10	0.0135	2	7.91E-05
14	Primary hypomagnesemia	12	0.0162	2	0.000116
15	Canavan disease	15	0.0203	2	0.000184
16	Adrenal hyperplasia, congenital, due to 3-beta-hydroxysteroid dehydrogenase 2 deficiency	18	0.0244	2	0.000267
17	Sepsis	23	0.0311	2	0.00044
18	Late-onset preeclampsia	40	0.0542	2	0.00134
19	Early preeclampsia	45	0.0609	2	0.00169
20	Amyotrophic lateral sclerosis	2	0.00271	1	0.00271
21	Autism	2	0.00271	1	0.00271
22	Brown-Vialetto-Van Laere Syndrome 1	2	0.00271	1	0.00271
23	Cystathioninuria	2	0.00271	1	0.00271
24	Folate deficiency	2	0.00271	1	0.00271
25	Galactosemia type 1	2	0.00271	1	0.00271
26	Headache	2	0.00271	1	0.00271
27	Leukemia	2	0.00271	1	0.00271
28	Long-chain-3-hydroxyacyl CoA dehydrogenase deficiency	2	0.00271	1	0.00271
29	Neurodegenerative disease	2	0.00271	1	0.00271
30	Wilson's disease	2	0.00271	1	0.00271
31	Pregnancy	766	1.04	5	0.00336
32	Adenosine kinase deficiency	3	0.00406	1	0.00406
33	Argininosuccinic aciduria	3	0.00406	1	0.00406
34	Hyperoxalemia	3	0.00406	1	0.00406
35	Hypoparathyroidism-retardation-dysmorphism syndrome	3	0.00406	1	0.00406
36	Hypothyroidism	3	0.00406	1	0.00406
37	Kidney cancer	3	0.00406	1	0.00406
38	Lysinuric protein intolerance	3	0.00406	1	0.00406
39	Short bowel syndrome	3	0.00406	1	0.00406

40	2,4-dienoyl-CoA reductase deficiency	4	0.00542	1	0.00541
41	Familial partial lipodystrophy	4	0.00542	1	0.00541
42	Histidinemia	4	0.00542	1	0.00541
43	L-2-Hydroxyglutaric aciduria	4	0.00542	1	0.00541
44	Leptin Deficiency or Dysfunction	4	0.00542	1	0.00541
45	Lipodystrophy, Congenital Generalized	4	0.00542	1	0.00541
46	Partial lipodystrophy	4	0.00542	1	0.00541
47	Phosphoribosylpyrophosphate Synthetase Superactivity	4	0.00542	1	0.00541
48	Pseudohypoaldosteronism, type I, autosomal dominant	4	0.00542	1	0.00541
49	Refractory localization-related epilepsy	4	0.00542	1	0.00541
50	Vitamin B12 deficiency	4	0.00542	1	0.00541
51	Biliary atresia	5	0.00677	1	0.00675
52	Cerebral creatine deficiency syndrome 2	5	0.00677	1	0.00675
53	Hypermethioninemia	5	0.00677	1	0.00675
54	Lesch-Nyhan syndrome	5	0.00677	1	0.00675
55	Pyruvate carboxylase deficiency	5	0.00677	1	0.00675
56	Short-chain L-3-hydroxyacyl-CoA dehydrogenase deficiency	5	0.00677	1	0.00675
57	Thymidine treatment	5	0.00677	1	0.00675
58	Carnitine palmitoyltransferase I deficiency	6	0.00812	1	0.0081
59	Cystic fibrosis	6	0.00812	1	0.0081
60	Proprotein Convertase 1/3 Deficiency	6	0.00812	1	0.0081
61	Pyruvate dehydrogenase phosphatase deficiency	6	0.00812	1	0.0081
62	Smoking	6	0.00812	1	0.0081
63	2-Ketoglutarate dehydrogenase complex deficiency	7	0.00948	1	0.00944
64	Anorexia nervosa	7	0.00948	1	0.00944
65	Citrullinemia type II, neonatal-onset	7	0.00948	1	0.00944
66	Hepatocellular carcinoma	7	0.00948	1	0.00944
67	Long-chain Fatty Acids, Defect in Transport of	7	0.00948	1	0.00944
68	Nicotinamide Adenine Dinucleotide Deficiency	8	0.0108	1	0.0108
69	3-Hydroxyacyl-CoA dehydrogenase deficiency	9	0.0122	1	0.0121
70	Bartter Syndrome, Type 4A, Neonatal, with Sensorineural Deafness	9	0.0122	1	0.0121
71	21-Hydroxylase deficiency	11	0.0149	1	0.0148
72	3-Hydroxy-3-methylglutaryl-CoA lyase deficiency	11	0.0149	1	0.0148
73	Chronic renal failure	11	0.0149	1	0.0148
74	3-Hydroxy-3-Methylglutaryl-CoA Synthase Deficiency	12	0.0162	1	0.0161
75	Celiac disease	12	0.0162	1	0.0161
76	Lipoyltransferase 1 Deficiency	12	0.0162	1	0.0161
77	Fumarase deficiency	13	0.0176	1	0.0175
78	Perillyl alcohol administration for cancer treatment	13	0.0176	1	0.0175
79	Diabetes mellitus type 2	21	0.0284	1	0.0281

80	Isovaleric acidemia	23	0.0311	1	0.0307
81	Cirrhosis	30	0.0406	1	0.0399
82	Obesity	745	1.01	2	0.267
